# Preliminary molecular characterization of the human pathogen *Angiostrongylus cantonensis*

**DOI:** 10.1186/1471-2199-10-97

**Published:** 2009-10-25

**Authors:** Hualiang He, Mei Cheng, Xiao Yang, Jinxiu Meng, Ai He, Xiaoying Zheng, Zhuoya Li, Pengjuan Guo, Zhihua Pan, Ximei Zhan

**Affiliations:** 1 Department of Parasitology, Zhongshan School of Medicine, Sun Yat-sen University, 74 Zhongshan, 2nd Road, Guangzhou, 510080, PR China; 2 Key Laboratory for Tropical Diseases Control, Ministry of Education, Sun Yat-sen University, Guangzhou, 510080, PR China; 3 Research Center of Medical Sciences, Guangdong General Hospital. Guangdong Academy of Medical Sciences. 96 Dongchuan Road, Weilun Bldg., Guangzhou, 510080, PR China; 4 Current address : College of Agriculture and Landscape Architecture, Zhongkai University of Agriculture and Engineering, Guangzhou, 510225, PR China

## Abstract

**Background:**

Human angiostrongyliasis is an emerging food-borne public health problem, with the number of cases increasing worldwide, especially in mainland China. *Angiostrongylus cantonensis *is the causative agent of this severe disease. However, little is known about the genetics and basic biology of *A. cantonensis*.

**Results:**

A cDNA library of *A. cantonensis *fourth-stage larvae was constructed, and ~1,200 clones were sequenced. Bioinformatic analyses revealed 378 cDNA clusters, 54.2% of which matched known genes at a cutoff expectation value of 10^-20^. Of these 378 unique cDNAs, 168 contained open reading frames encoding proteins containing an average of 238 amino acids. Characterization of the functions of these encoded proteins by Gene Ontology analysis showed enrichment in proteins with binding and catalytic activity. The observed pattern of enzymes involved in protein metabolism, lipid metabolism and glycolysis may reflect the central nervous system habitat of this pathogen. Four proteins were tested for their immunogenicity using enzyme-linked immunosorbent assays and histopathological examinations. The specificity of each of the four proteins was superior to that of crude somatic and excretory/secretory antigens of larvae, although their sensitivity was relatively low. We further showed that mice immunized with recombinant cystatin, a product of one of the four cDNA candidate genes, were partially protected from *A. cantonensis *infection.

**Conclusion:**

The data presented here substantially expand the available genetic information about the human pathogen *A. cantonensis*, and should be a significant resource for angiostrongyliasis researchers. As such, this work serves as a starting point for molecular approaches for diagnosing and controlling human angiostrongyliasis.

## Background

*Angiostrongylus cantonensis *was first discovered in the pulmonary arteries and hearts of domestic rats in Guangzhou (Canton), China, by Chen in 1935 [1]. This metastrongyloid nematode is now well recognized as the primary cause of human eosinophilic meningoencephalitis (EME) in many parts of the Indo-Pacific region [2,3]. Rats, the permissive or definitive host, acquire this nematode by ingesting the third-stage (L3) larvae. Humans, as accidental hosts, become infected by eating raw or improperly cooked freshwater snails, the intermediate host of this nematode, or paratenic hosts such as monitor lizards [4], shrimp [5], frogs [6], fish and slugs [7]. The parasite larvae ultimately reach the central nervous system (CNS) or occasionally migrate to the eye chamber. The three main clinical manifestations of human angiostrongyliasis are eosinophilic meningitis (EoM), eosinophilic encephalitis (EoE) and ocular angiostrongyliasis [2,8].

Nomura and Lin reported the first human infection with *A. cantonensis *in Taiwan in 1945. Following that, cases were reported in many countries and regions, primarily Australia [9,10], the southwestern Pacific [11,12], southern and southeast Asia, Africa [13], the Caribbean [14] and southeastern USA [15]. Reports of all forms of human angiostrongyliasis are increasing due to frequent international travel [11,16-18]. The first human case in mainland China was reported in 1984, and only four cases were reported between 1984 and 1994. However, more than 300 cases of human angiostrongyliasis were documented from 1994 to 2006 [3,19,20]. A large outbreak involving 141 patients occurred in 2006 in Beijing, in northern China [21]. Another outbreak involving 33 confirmed cases occurred in 2008 in Yunnan province in southwestern China. Moreover, owing to the past and projected effects of global climate change [22,23], there are vast habitats worldwide, including mainland China, with environmental conditions potentially suitable for intermediate hosts of *A. cantonensis *[24,25]. Human angiostrongyliasis is becoming a new public health problem in mainland China; however, clinicians remain unfamiliar with the disease.

There has been much investigation into the serological diagnosis [26,27] and treatment [28] of human angiostrongyliasis in past decades, but molecular characterization of the causative agent of angiostrongyliasis, *A. cantonensis*, has received less attention. *A. cantonensis*, has only 13 documented gene sequences, including sequences for 2 transposons [29], 4 noncoding RNA genes [30,31], and 7 protein-coding genes [32,33]. A total of 1,276 EST sequences have also been documented. In 2004, Peng H.J. submitted 50 adult worm ESTs (, Accession Number [GenBank: CV826669~CV826718]). And in 2005, Tang, P. and Wang, L.C. submitted 1,226 EST sequences from fifth stage larvae obtained from the brains of laboratory-infected Sprague Dawley rats (, Accession Number [GenBank: ND190143~ND191368]). The biology of this nematode is still far from clear. Furthermore, despite the fact that human angiostrongyliasis has become a new public health problem [34], genomic or EST sequencing of *A. cantonensis *are not included in international research plans for the foreseeable future. In this study, we cloned and analyzed full-length cDNA sequences from this nematode, identifying 378 cDNA clusters. By applying bioinformatic and immunogenicity analyses, we have improved our understanding of the basic biology of *A. cantonensis*.

## Results

### Comparative analysis of sequenced cDNAs

**Figure 1:**
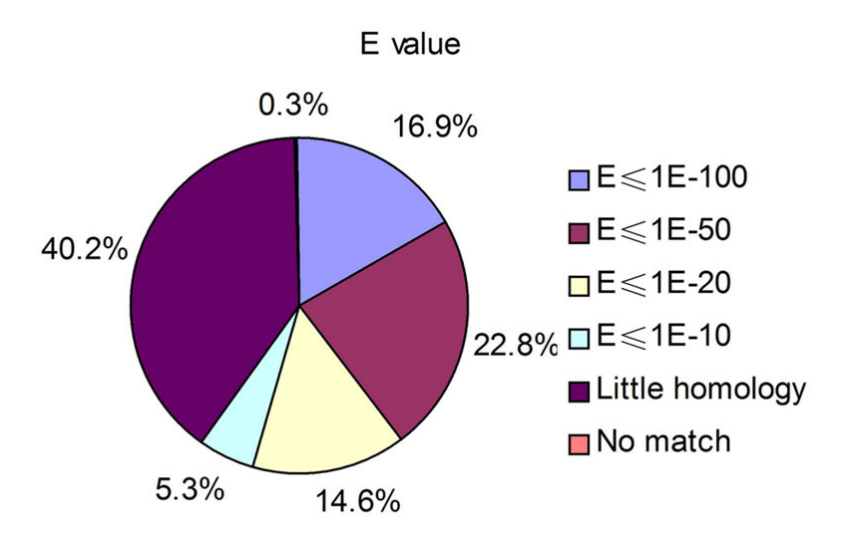
**Comparative analysis of the 378 *A. cantonensis *cDNA sequences**. 'Little homology' indicates cDNA sequences in which the highest E value from homology analysis is larger than 1E^-10 ^or other sequences for which identity < 25% or homology length < 30 amino acids. "No match" indicates absence of homology to known genes or EST sequences.

As an initial step towards sequencing the transcriptome of this nematode, we constructed a full-length cDNA library from the tissues of fourth-stage (L4) larvae obtained from mouse brains. First, we sequenced an approximate 600-bp 5' terminal region from ~1,200 cDNA clones. After excluding any repetitive DNA, rDNA and ambiguous sequences, about 1,000 clones remained. These 1,000 sequences were used to interrogate the public databases by BlastX and were assigned to 378 cDNA clusters. Next, we selected a single clone from each cDNA cluster and sequenced the complete input cDNA fragment. Of these 378 cDNAs, 205 (54.2%) and 225 (59.5%) matched known genes at cutoff expectation values (E) of 10^-20 ^and 10^-10^, respectively (identity ≥ 25%, homology length ≥ 30 amino acids; Figure 1). About 40% of cDNAs showed little homology to known genes in the searched databases. In addition, a holistic comparative analysis was performed among these 378 cDNA clusters and genetic resources for hosts of *A. cantonensis *and for *Caenorhabditis elegans*. Of these 378 sequences, at least 124 (32.8%), 123 (32.5%), 124 (32.8%), and 189 (50.0%) were orthologous with human, mouse, rat and *C. elegans*, respectively (E ≤ 10^-20^).

Prior to submitting our cDNA sequences, 1,276 EST sequences from *A. cantonensis *had been deposited in the GenBank database. Of these, 1,226 were from L5 larvae and 50 were from adult worms recovered from rats. We analyzed our 378 cDNA clusters from L4 larvae recovered from mouse for correlations with these 1,276 EST sequences. We found that 50 of 378 cDNA clusters covered 190 individual EST sequences (50 EST clusters) of the 1,226 EST sequences from L5 larvae. Especially, we found that 3 cDNAs cluters of COLlagen family members had covered 68 ESTs, more than one third of above 190 individual EST sequencess. And 8 cDNA clusters covered 11 of 50 individual EST sequences (8 EST clusters) from adult worms. In addition, four cDNA clusters were common among L4 larvae, L5 larvae and adult worm cDNA libraries, implying the constitutive expression profile. These four cDNAs were transcripts of myosin light chain family member 3, putative collagen protein 140, putative stress-activated protein kinase JNK-1, and heat shock protein family member 3.

### Biological characterization of 168 full-length cDNAs

**Figure 2:**
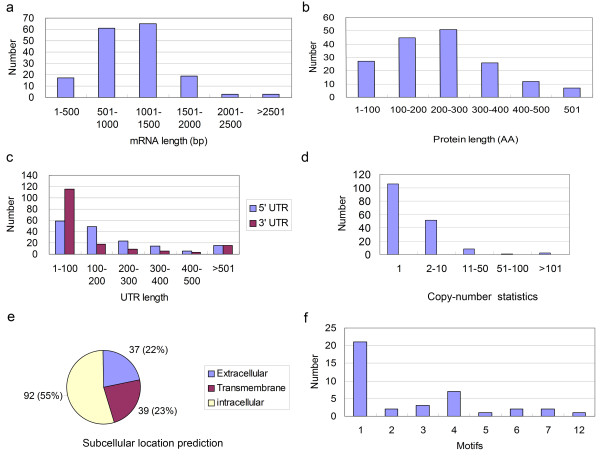
**Biological characterization of 168 full-length cDNAs of *A. cantonensis***. (a) Length distribution of 168 full-length cDNAs (in bp). (b) Length distribution of proteins (in amino acids; AA) deduced from predicted ORFs of the 168 full-length cDNAs in a. (c) Length-distribution of UTR regions (in bp) calculated from UTR length relative to entire cDNA length, expressed as a percentage. (d) Copy-number statistics for each cDNA cluster derived from counts of cDNA copies in each cDNA cluster isolated in the first set of 1200 clones. (e) Distribution of the predicted subcellular locations of the putative protein products of full-length cDNAs. (f) Number distribution of predicted transmembrane motifs of proteins deduced from ORFs as in a.

Among these 378 cDNAs, only 168 were found to contain a complete open reading frame (ORF; [see Additional File 1]). These 168 full-length cDNAs had an average length of 1,080 bp. There were 124 (73.8%) cDNAs with 501-1,500 bp, 17 (10.0%) smaller than 500 bp, and 27 (16.1%) larger than 1,500 bp (Figure 2a). The corresponding 168 proteins had an average length of 238 amino acids (Figure 2b); the 5'-untranslated regions (UTRs) averaged 225 bp and 3'-UTRs averaged 149 bp in length. The protein with the largest molecular weight was a putative "PAP/25A-associated domain containing" protein consisting of 694 amino acids, whereas the smallest protein was a putative transcription factor 37 amino acids in length. An analysis of the 3'-UTR length distribution of the 168 cDNAs showed that 59 cDNAs contained 3'-UTRs shorter than 100 bp. In addition, approximately 45% of clones had 3'-UTRs that were at least half the length of the corresponding cDNA (Figure 2c). Although these cDNAs contained an ORF, some cDNA sequences did not contain full 5'-UTRs; thus, the length distribution of 5'-UTR are not described here.

The copy numbers for each cDNA cluster in this first set of clones were statistically analyzed by counting the redundant cDNA copies in each cluster, yielding an average for the 168 cDNA clusters of 3.68 copies. Of the 168 cDNA clusters, 106 (63.1%) were present in only a single copy (Figure 2d), indicating a relatively low level of expression. Interestingly, there were 136 copies of transcripts for collagen-140, the largest number for any transcript in this first round of sequencing. Such a relatively high level of transcript for a collagen protein family member is possibly related to the third or fourth molting of larvae after migrating into the mouse brain. Such statistics about clone abundance of individual cDNA clusters provide some important insights into the dominantly expressed genes of L4 larvae in the mouse brain.

Excretory/secretory proteins and surface membrane proteins of parasitic helminths are known to be good candidates for the diagnosis of helminthosis. We thus attempted to identify cDNAs that encode secretory signal peptides or transmembrane domains. The putative protein products of these 168 cDNAs were screened using the analytical tools SignalP and TMHMM. This analysis showed that 58 (34.5%) of these proteins, including proteins such as putative collagen family members, putative proteases and putative lipid-binding proteins, contain a signal peptide or signal anchor (Figure 2e; [see Additional File 2]). Several proteins also contain one or more transmembrane domains (Figure 2f; [see Additional File 3]). Some proteins in this group, containing up to four or seven transmembrane domains, likely belong to membrane-bound receptor families involved in signal transduction.

### cDNA-encoded protein function and larval metabolism

**Figure 3:**
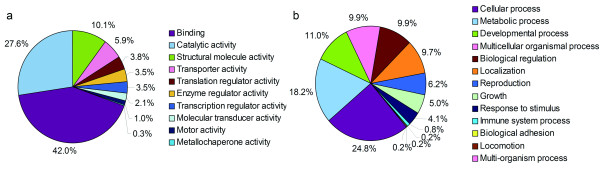
**Predicted functions of *A. cantonensis *cDNA products based on Gene Ontology**. Distribution of major categories of "molecular function" (a) and "biological process" (b), based on the predicted functional domains of putative cDNA protein products.

To predict the function of cDNA-encoded proteins, we independently classified the predicted translations of these cDNAs into different functional categories based on Gene Ontology (GO). Of these 378 cDNAs, only 185 were successfully assigned to the 10 main "molecular function" categories of GO. Most cDNA clusters belonged to the categories "binding" (n = 121) and "catalytic activity" (n = 81), which are not mutually exclusive (Figure 3a, and [see Additional File 4]). The 121 cDNAs predicted to have binding functions were further subcategorized into the primary terms "protein binding" and "ATP binding", which accounted for 44 (36.4%) and 22 (18.1%), respectively, of the total. The 81 cDNAs predicted to encode proteins with catalytic activity could be further defined by 90 terms. However, unlike the proteins with predicted binding function, there were few predominant subcategories for those predicted to possess catalytic activity. The term "GTPase activity" was the most common, but applied to only seven cDNAs. The putative functions of the 378 cDNA-encoded proteins were also analyzed based on the GO "biological process" hierarchy (Figure 3b, and [see Additional File 4]). We found a number of cDNAs that may be associated with larval development and/or positive regulation of growth. Such a gene distribution might reflect the basic need for larval development from L3 to L4 and from L4 to L5 in the mouse brain.

The infectious third-stage larvae of *A. cantonensis *usually secrete proteolytic enzymes after infecting the host. The functions served by different protease families include degradation of host hemoglobin [35-37], larval penetration and migration [38-40], and immune evasion [41-45]. Of the 90 functional terms identified under the category of "catalytic activity", more than 10% are proteases, including serine, cysteine and aspartic proteases (Table 1). The copy number of cysteine and aspartic protease family members in this first set of clones ranged from 1 to more than 10 copies. The total number of cDNA copies belonging to the aspartic protease family was approximately equal to that of the cysteine protease family, but was five times larger than the number of cDNA copies corresponding to the serine protease family. However, none of the cDNAs was found to be homologous to metalloproteases, one of the four main protease families. In addition to proteases, *A. cantonensis *expresses enzymes involved in different metabolic pathways [see Additional File 5]. About 20 enzymes identified here were associated with protein metabolism and 10 were involved in energy metabolism. However, only two enzymes involved in glycolysis were found, namely fructose-bisphosphate aldolase 2 (Aldolase CE-2) and lactic acid dehydrogenase (LDH). In summary, the distribution of cDNA-encoded functional domains in the first set of 1,200 clones suggests that transcripts for enzymes involved in protein metabolism and energy metabolism are more predominant during larval development in the mouse brain than those related to the glycolytic pathway.

**Table 1:** cDNA clones predicted to encode protease family proteins

**Clone No.**	**N**	**Homologous gene [organism]**	**Protease family**	**Functions**
0009D10	1	Serine protease pcp-1 precursor 1 [*C. elegans*]	Serine protease	Facilitates penetration and migration [38-40]; Immune evasion [41,43]; Lysosomal protection
0005E12	3	Cathepsin A [*Monodelphis domestica*]		

00012B11	1	Cysteine proteinase [*Haemonchus contortus*]		
0007D03	1	Cathepsin B-like cysteine protease 2 [*Parelaphostrongylus tenuis*]		
0014F02	2	Cathepsin B-like cysteine protease 1 [*P. tenuis*]	Cysteine protease	Degradation of hemoglobin and serum albumin [36]; Iron acquisition; Larva molting; Degradation of phagocytosed material; Immune evasion [44,45]
0005F12	4	Cathepsin L 1 [*Dictyocaulus viviparus*]		
00012F09	10	CathePsin Z family member (cpz-1) [*C. elegans*]		

15G12	1	Aspartyl protease family member (asp-1) [*C. elegans*]		[[Bibr B42]]
0006G04	2	Aspartyl protease protein 6 [*C. elegans*]	Aspartyl protease	Digestion of hemoglobin serum protein [35,37]; Intestinal digestion and tissue degradation; Initiates a catabolic pathway; Immune evasion [42]
00010A09	3	Aspartyl protease family member (asp-2) [*C. elegans*]		
16B10	14	Cathepsin D-like aspartic protease [*Ancylostoma ceylanicum*]		

N indicates total number of cDNAs in the corresponding cDNA cluster.

It is becoming clear that parasitic nematodes have evolved highly specialized mechanisms to modify the host tissue environment to their benefit. In this context, the lipid metabolic pathway could be particularly important because L4 and L5 larvae of *A. cantonensis *are surrounded by the luxuriant cephalin in the brain of host. In this study, eight of the enzyme-associated genes identified, including fatty acid desaturase and acid sphingomyelinase, are involved in lipid metabolism. In addition, other non-enzyme-associated genes related to lipid metabolism were also found, including putative fatty acid and retinol-binding protein (FRbp), ADIPOR-like receptor and lipid binding protein (Lbp) family members. FRbp may facilitate the uptake, transport and distribution of fatty acids and retinols to specific target tissues [46]. The ADIPOR-like receptor mediates fatty-acid oxidation through activation of AMP kinase and PPAR-alpha [47]. Such information could indicate that *A. cantonensis *obtains lipids from the host to synthesize sterols and fatty acids. In brief, the observed pattern of genes involved in protein metabolism, lipid metabolism and glycolysis may reflect the central nervous system habitat of this pathogen.

### Immune responses and diagnostic potential of recombinant protein candidates

To identify antigens that might be valuable for diagnosing human angiostrongyliasis, we selected several representative proteins from different cellular location for further study. Four recombinant proteins were successfully purified in the initial attempt. These four proteins were cystatin (possibly extracellular), cathepsin D-like aspartic protease (possibly extracellular), intermediate filaments (IFs; cellular structural component) and lactic acid dehydrogenase (LDH; cytoplasmic). Conserved features of these four gene products were shown [see Additional File 6]. These recombinantly expressed proteins were then used for immunogenicity analysis. Immunoblotting results were analyzed using four groups of serum sample (Table 2, and [see Additional File 7]). The ability to recognize proven human angiostrongyliasis (sensitivity) was highest for crude somatic antigen and excretory/secretory (ES) antigen (100%), intermediate for cystatin and aspartic protease (75%) and lowest for IFs and LDH (50%). None could recognize all sera from clinically suspected angiostrongyliasis groups, although the ES antigen was the most responsive. The corresponding specificities were 92.3% and 80.8% for crude somatic antigen and excretory/secretory (ES) antigen, respectively, and 100% for cystatin, aspartic protease, IFs and LDH. No sera from the healthy control group or other parasitic diseases groups reacted with these four candidate proteins. In summary, the specificity of the two extracellular proteins was superior to that of the two intracellular proteins. In addition, the specificity of all four recombinant proteins was superior to that of crude somatic antigen and ES antigen, but the opposite was true with respect to sensitivity.

**Table 2:** Summary of IgG antibodies against different antigens of *A. cantonensis *demonstrated by immunoblotting

**Group**	**Number of sera tested**	**Number of sera recognized by candidate antigens**					
		
		**Crude somatic antigen**	**ES**	**Aspartic protease**	**Cystatin**	**IFs**	**LDH**
Proven human angiostrongyliasis	4	4	4	3	3	2	2
Suspected human angiostrongyliasis	11	6	8	2	3	2	0
Other parasitic diseases	22	2	5	0	0	0	0
Healthy controls	4	0	0	0	0	0	0

ES, excretory/secretory antigen; aspartic protease (Clone NO. 16B10); cystatin (NO. 00011F08); IFs, intermediate filaments (NO. 00010H11); LDH, lactic acid dehydrogenase (NO. 0008A11).

### Immunogenicity and vaccine potential of recombinant protein candidates

To investigate the immunogenicity of candidate proteins, we first immunized mice in four experimental groups by subcutaneous injection with recombinant cystatin, aspartic protease, IFs or LDH, and then detected the IgG level in the mouse by indirect ELISA. We found that IgG against the corresponding recombinant protein increased rapidly after day 7 and peaked on day 14 in all four experimental groups. A control group injected with PBS showed no such response. Thus, all of these four recombinant proteins were capable of inducing an immune response in mice.

After immunizing mice with these recombinant proteins, we challenged experimental and control groups with L3 larvae, and evaluated mouse survival. By day 21 post-challenge, more than 50% of mice had died in the control group and in the experimental groups immunized with recombinant aspartic protease, IFs or LDH. However, 90% of mice survived in the experimental group immunized with cystatin. To confirm the vaccine potential of recombinant cystatin, we examined changes in worm burden in the brains of surviving mice by pathological analysis. We found that there was a significant difference in the average worm burden between the cystatin and control groups (P < 0.01). In mice immunized with cystatin, the worm burden was reduced by 52.3% (Table 3), as shown in brain sections from mice [see Additional File 8]. A trend toward lower worm burden changes was also found in the other three experimental groups, but the difference between experimental and control groups did not reach statistical significance. In summary, although specific antibodies against these four recombinant proteins could be induced, only the group immunized with cystatin acquired partial protection against *A. cantonensis*.

**Table 3:** Immune-protective effects of recombinant cystatin

**Group**	**Number of mice**	**Cystatin immunization**	**Number of surviving mice (21 days)**	**Average number of larvae recovered (x ± SD)**	**Reduction in number of larvae**
A	20	No (negative control)	8	27.7 ± 8.45	/
B	20	Yes	18	13.2 ± 4.52*	52.3%

*Significantly different compared to control group (P < 0.01). After immunization, each mouse in two groups was challenged with 40 infective larvae (L3) of *A. cantonensis*. The challenge experiment and protection analysis were performed once. Each experimental group was composed of two parallel groups, each containing 10 mice.

## Discussion

In recent decades, relatively little attention has been paid to the nematode *A. cantonensis*, primarily because there have been few cases of human angiostrongyliasis. However, vast habitats with environmental conditions suitable for intermediate hosts of *A. cantonensis *exist in many places in mainland China [24,25]. Thus, continuing to ignore this disease may no longer be an option. In this study, we reported our findings on the genetics of *A. cantonensis*, identifying 378 cDNA sequences derived from L4 larvae isolated from mouse brains. These 378 cDNAs and their putative protein products were then subjected to further *in silico *analyses.

### Host-parasite interplay

Self-healing may be rapid in some cases of human angiostrongyliasis. We made a similar observation in mice: although some mice in experimental groups died within several days of being infected, others partially eliminated larvae from their bodies and survived. Whether a co-adaptation between this nematode and its non-permissive host occurred is not yet clear, but such observations are suggestive of interplay between *A. cantonensis *and its host.

Earlier studies on parasitic nematodes have shown that proteases are key factors for host-parasite interactions. Because the larvae of *A. cantonensis *do not possess a buccal stylet, mucosal penetration is probably facilitated by tissue-degrading proteases. In this study, three of the four main protease families were identified, namely serine, cysteine and aspartic proteases. However, no transcripts of the fourth protease family, metalloproteases, were identified in this first set of clones, indicating that metalloproteases are expressed at low levels. A previous study demonstrated that serine proteases and metalloproteases secreted by this nematode were key to larval penetration of the mouse intestine [40]. The protease profile demonstrated here, in particular, the absence of metalloproteases, likely reflects differences in intestinal and CNS parasitic modes. Once larvae have penetrated the mouse intestine and entered the CNS, they probably no longer need to excrete high levels of metalloprotease. In fact, continued expression of certain metalloproteases in the brain could be detrimental to parasite survival. To resist infections, the host usually secretes several factors that directly or indirectly eliminate challenging parasites. Studies on blood-brain barrier (BBB) dysfunction in patients with EOM caused by *A. cantonensis *have shown that entry of leukocytes into the CNS is dependent on the expression of matrix metalloproteinases (MMPs) [48,49]. Thus, the absence of metalloproteinase expression in L4 *A. cantonensis *larvae may contribute to host-parasite interplay by blunting the host immune response.

We also identified transcripts that encode other possible contributors to host-parasite interplay, including cystatin, a cysteine protease inhibitor, and FRbp, which is involved in lipid metabolism. Secretion of cystatin by larvae is associated with inhibition of the activity of immune killer cells of the host [50]. Bm-CPI-2, a homolog of cystatin secreted by the filarial parasite *Brugia malayi*, inhibits class II MHC-restricted antigen processing [51]. The product of the FRbp gene is probably also involved in host-parasite interplay. A protein homologous to FRbp that is secreted by adult *Ancylostoma caninum *hookworms increases the infective abilities of larvae by reducing the amount of retinol available for repair of host tissue damage [46]. The expression patterns of the genes discussed above could serve a starting point for the study of host-parasite interplay in the mouse CNS.

Permissive hosts, such as the rat, acquire this nematode by ingesting L3 larvae. The larvae enter the bloodstream and reach the CNS, where they molt twice to become adult worms in 2 weeks. The adult worms migrate to the pulmonary arteries, where they develop to sexual maturity and lay eggs [3]. However, in non-permissive hosts (such as mice and humans), the larvae are incapable of developing into adult worms and usually inhabit the CNS. It has been reported that nematode development depends on (or responds to) endocrine and immune signals of the host [52-54]. In the current study, several putative membrane receptors were found, including the gonadotropin-releasing hormone receptor (GnRHR), which regulates early development of the testis and ovary [55], and the somatostatin receptor. We found that several conserved regions within the intracellular or extracellular loop of GnRHR were homologous between *A. cantonensis *and hosts (human, mouse and rat), consistent with the idea that this nematode may be responsive to host hormonal signals. Differences in parasite development and behavior may depend on such receptors, through receipt of different signals from different hosts. Thus, developing a clearer understanding of the receptor types expressed by *A. cantonensis *could be important to understanding different parasitic behaviors in different hosts. Going forward, our research on host-parasite interplay will focus on protease families and putative receptors.

### Diagnosis and treatment of angiostrongyliasis

Several effective antigens, identifiable as immunoreactive bands, have been successfully used to diagnose human angiostrongyliasis [26,27,56-58], but their amino acid sequences are not known. The absence of such information could be a significant limitation to large-scale production of engineered antigens. In this study, the individual recombinant proteins selected as antigens were not sufficiently sensitive to diagnose human angiostrongyliasis, and sensitivity was not improved using mixed antigens with different combinations of these four recombinant proteins. Thus, at this stage, the sensitivities of the candidate proteins did not meet the criteria of a diagnostic kit. However, ES antigens and extracellular proteins (aspartic protease, cystatin) seemed to more readily recognize positive sera than did proteins lacking excretory/secretory properties. Thus, future efforts will focus on engineering proteins from this cDNA library with excretory/secretory properties.

Although most cases of human angiostrongyliasis are mild and self-limiting, death can occur in severe cases without prompt and proper treatment [3,59,60]. Anthelmintic drugs, such as albendazole and mebendazole, are usually not recommended for treatment of *A. cantonensis *infections owing to the possibility of exacerbating neurological symptoms [28,61]. Illuminating the basic biological behavior of larvae could provide clues for a more effective treatment strategy. Based on Gene Ontology, we found that the putative products of a number of cDNAs were involved in lipid metabolism and signal transduction pathways. More knowledge of relevant metabolic and signal transduction pathways will provide valuable information that could aid the identification potential drug targets for the future treatment of human angiostrongyliasis. The continued maintenance of *A. cantonensis *in a larval stage in the CNS accounts for the insidious quality of human angiostrongyliasis. Determining the mechanisms underlying the developmental behavior of *A. cantonensis *in different hosts may make it possible to induce larvae to migrate into pulmonary arteries of humans before administering a drug treatment program, thereby avoiding acute CNS tissue damage.

## Conclusion

Although far from representing a comprehensive genome-wide gene expression study, the 378 cDNAs identified here substantially increase the available genetic information about *A. cantonensis*. This study provides data that can be expected to aid angiostrongyliasis researchers in their selection of genes for future studies. The bioinformatic analysis described here establishes a framework for exploring the basic biology and biomedical implications of this dangerous nematode. Larval metabolic pathways and host-parasite interactions are important to understanding the molecular basis of parasite behavior in different hosts. Our future studies will focus on a comprehensive analysis of protein function and identification of more effective diagnostic antigens. We also note the need to make a concerted effort to improve the control of human angiostrongyliasis, which is an increasing public health problem in mainland China.

## Methods

### Preparation of parasite larvae and experimental animal materials

L3 larvae of *A. cantonensis *were obtained from its intermediate host, the wild giant African snail, *Achatina fulica*, captured in Guangzhou, China. The larvae within tissues were recovered by a previously reported method [62,63]. BALB/c mice (male, 6-week-old) were purchased from the animal center laboratory at Sun Yet-sen University, Guangzhou, China. Mice were maintained in a 12-h light/dark cycle for more than 1 week prior to infection with 40 infective larvae per mouse by oral inoculation. Twenty-one days later, mice were sacrificed by cervical dislocation. All procedures involving animals and their care described here were approved by the Institutional Animal Care and Use Committee of Sun Yet-sen University and were performed in accordance with institutional guidelines for animal experiments.

### cDNA library construction, sequencing and bioinformatic analysis

Previous studies have characterized larval development in the non-permissive mouse host. Larvae can migrate into the brain of a mouse within two days after infection. After 10 days, most larvae remain in the 3rd stage, but a few develop into 4th-stage larvae. After 10 to 21 days, most larvae develop into 4th stage, and begin to develop into 5th stage from 21 to 30 days[64]. However, mouse usually shown acute immune response in the brain because of some dead L4 or L5 larvae being cleared. Given this developmental sequence, L4 larvae parasiting in the brain within 21 days appeared to be a representative stage to use for learning about the basic biology and life cycle of this parasitic nematode in mice. Then, about 200 L4 larvae of A. cantonensis were collected from the brains of a mouse, immersed in TRIzol Reagent (Gibco BRL) and frozen immediately. mRNAs were collected using Oligotex mRNA Kits (QIAGEN) from total RNAs of L4 larval tissue. cDNA was synthesized from total RNA by reverse transcription using SMARTTM cDNA Library Construction Kit (CLONTECH). We combined the following reagents in a sterile 0.5-ml microcentrifuge tube (1-3 ul RNA sample, 1 ul SMART IV Oligonucleotide, 1 ul CDS III/3' PCR Primer) and incubate the tube at 72°C for 2 min and then cool the tube on ice for 2 min. We then add the following to each reaction tube to10.0 ul total volume (2.0 ul 5× First-Strand Buffer, 1.0 ul DTT (20 mM), 1.0 ul dNTP Mix (10 mM),1.0 ul PowerScript Reverse Transcriptase). And incubate the tube at 42°C for 1 hr and then place the tube on ice to terminate first-strand synthesis. After that, cDNA was purified using Chroma Spin-400 columns (CLONTECH). cDNA libraries were prepared by directional cloning of purified cDNA into the pBluescript II SK vector (Addgene), according to the manufacturer's instructions, and screened on LB medium (Ap^r^-IPTG/x-gal). In the first round, approximately 1,200 clones were selected randomly and were subjected to DNA sequencing. Bioinformatic analyses of cDNA sequences were carried out using the basic local alignment search-tool family of programs (e.g., BLAST, ); SignalP 3.0 server, ; TMHMM 2.0 server, ). ORF predictions were based on results of BlastX and ORF finder . Homology to any given well-defined gene was determined from the highest E value of homology analysis by interrogating cloned cDNA against public databases. Predictions of putative cDNA product function were made by reference to the Gene Ontology database .

### Preparation of native somatic antigen, ES antigens and recombinant proteins

Crude somatic antigen of *A. cantonensis *was prepared by chopping recovered L4 larvae and homogenizing on ice for 15 minutes. After storing at 4°C overnight, homogenates were centrifuged at 15,700 × g for 15 minutes and the supernatant (crude somatic antigen) was collected. ES antigen was prepared by incubating the recovered L4 larvae in Waymouth's medium MB 752/1 (GIBCO) at 37°C in a 5% CO_2 _atmosphere according to method of Hata [65]. Medium was collected on the third day of incubation and concentrated using a membrane concentrator with a 10-kDa molecular weight exclusion size (Millipore).

For subcloning cDNA into the pET32a (+) expression vector (Novagen), DNA restriction enzyme recognition sites were introduced at the 5'- and 3'-ends of the candidate cDNA by inclusion in forward and reverse primers. The following primers were used to amplify cDNAs encoding the indicated proteins (restriction sites were shown in bold and italic): cathepsin D-like aspartic protease, 5'-GTC***GGTACC***ATGAGAGGAGAAT-3' (forward primer, containing *Kpn*I site) and 5'-GCA***GAGCTC***TCAGTCAAATACGT-3' (reverse primer, containing *Sac*I site); cystatin, 5'-CT***GAATTC***ATGGTCGGAGGTCGT-3' (forward primer, containing *EcoR*I site) and 5'-CG***CTCGAG***TTACAGCTCTTCATC-3' (reverse primer, containing *Xho*I site); intermediate filaments (IFs), 5'-GC***GGATCC***CTAGTCAAATTGTCGAATATTG-3' (forward primer, containing *BamH*I site) and 5'-GC***GTCGAC***TTACGTAGCGCTTTGACTC-3' (reverse primer, containing *Sal*I site); lactic acid dehydrogenase (LDH), 5'-CG***GAATTC***ATGAACTGCGAGACTGC-3' (forward primer, containing *EcoR*I site) and 5'-CC***CTCGAG***TCACAACTGAAGCTTATTCT-3' (reverse primer, containing *Xho*I site). With the exception of the cDNA for LDH, none of these cDNAs contained completely ORFs.

Recombinant proteins were expressed in vitro as described by Lv [66]. Briefly, after transforming *Escherichia coli *BL21 (DE3) with the appropriate expression construct, expression of His-tagged candidate proteins was induced by adding isopropyl-1-thio-β-Dgalactopyranoside (IPTG) at a final concentration of 0.4 mM. Soluble protein was purified and thrombin-cleaved under native conditions by Ni-NTA chromatography following the manufacturer's instructions (Novagen). The eluted protein fractions were analyzed by sodium dodecyl sulfate polyacrylamide gel electrophoresis (SDS-PAGE) on 15.0% gels, and protein concentration was determined using a DCTM protein assay kit (Bio-Rad). The purified recombinant protein was confirmed by mass spectrometry using an ABI 4700 Proteomics Analyzer TOF/TOF (Applied Biosystems, USA).

### Protein immunogenicity assessment, immunization schedule and challenge infection

All serum samples from four proven and 11 clinically suspected cases of human angiostrongyliasis were acquired from the Centers for Disease Control and Prevention (CDC) in several cities in China. Serum samples from healthy controls and individuals infected with other parasitic diseases had been previously collected and preserved in our laboratory. Blood samples from mice infected with *A. cantonensis *were collected from Balb/c mouse infected with 40 larvae. Serum samples collected before infection (day 0) served as controls. All sera were separated by centrifuging blood samples at 9,300 × g for 20 minutes in a refrigerated centrifuge and stored at -20°C until ready for use. IgG fractions were affinity purified over a HiTrap™ Protein G HP column (GE Healthcare/Amersham Biosciences) and quantified. Pre-immune mouse serum was processed similarly to provide reagents for negative controls.

For each recombinant protein, 20 mice were randomly divided into two groups of ten mice each. Mice in the immune-challenge group were injected subcutaneously with 50 μg recombinant protein dissolved in phosphate buffered saline (PBS) with complete Freund's adjuvant (Sigma); 20 mice receiving no antigen injection served as a challenge-control group. The mice in experimental groups were boosted subcutaneously twice with the same amount of antigen with incomplete Freund's adjuvant (Sigma) at 2-week intervals. Mice in the adjuvant-treated control group were subjected to the same immunization schedule as the immune-challenge group, but received PBS in place of recombinant protein. One week after the third immunization, mice in both immune-challenge and control groups were challenged with 40 ± 1 infective larvae (L3) of *A. cantonensis*. After 21 days, mice were sacrificed and brains were immediately transferred to PBS for statistical analysis of worm burden or were fixed in 10% formalin for histopathological examination. After fixation, brains were embedded in paraffin, sliced into 4-5-mm-thick coronal sections and stained with hematoxylin and eosin. The stained sections were then examined under a light microscope. Worm burden reduction was calculated as ([M-N]/M) × 100%, where M is the average worm burden in the control group, and N is the average worm burden in the immunized group.

### Analysis of specific antibody responses by indirect ELISA

IgG antibodies against *A. cantonensis *in human serum were detected by ELISA, as previously described [67]. Briefly, each well in a Maxisorp 96-well microtiter plates was coated overnight at 4°C with 10 μg/100 μl of recombinant protein or crude somatic antigen and ES antigen in carbonate-bicarbonate buffer. Wells were subsequently blocked for 2 hours at 37°C with 3% BSA in PBS (pH 7.2) containing 0.05% Tween-20 (PBST). Human serum samples were serially diluted and added to each well. To detect antibodies in serum samples, 100 μl of horseradish peroxidase-conjugated goat anti-human IgG secondary antibodies (Santa Cruz Biotechnology, Inc.), diluted 1:3,000, was added and incubated for 45 minutes at 37°C. After washing plates three times with PBST, 100 μl peroxidase substrate (3,3',5,5'-tetramethylbenzidine) was added to each well. Plates were then incubated at 37°C for 10 minutes and 50 μl H_2_SO_4 _(0.2 M) was added to stop the reaction. The plates were read at wavelengths of 450/620 nm using an automated plate reader (BioRad, Hercules, CA, USA). IgG antibodies against recombinant protein in mouse serum were measured by the same method using goat anti-mouse IgG as a secondary antibody.

A value corresponding to the mean optical density (O.D.) from ELISA assays of control subjects plus three standard deviations (SD) was calculated and used as the cut-off level to define a positive sample. Sensitivities and specificities of antigens were analyzed as described by Intapan [68], using the following relationships: Sensitivity = number of true positives/(number of true positives + number of false negatives); Specificity = number of true negatives/(number of true negatives + number of false positives). The reliability of immunodiagnosis for human angiostrongyliasis was evaluated according to the procedures of Gjorup [69]. Consistent data were obtained from all tests, indicating the absence of day-to-day variation.

### Statistical analysis

Data were expressed as the mean ± standard SD. P-values < 0.05, determined by Student's *t*-test, were considered significant.

### WebIn (EMBL WWW Submission System) accession numbers

Of the 378 cDNA sequences identified here, 240 have been submitted to the European Bioinformatics Institute (EBI)  under the EMBL accession numbers, [EMBL: FM207661~EMBL: FM207900].

## Abbreviations

L3: third-stage larva; L4: fourth-stage larva; CNS: central nervous system; EME: eosinophilic meningoencephalitis; EoM: eosinophilic meningitis; EoE: eosinophilic encephalitis; ORF: open reading frame; UTR: untranslated region; BBB: blood-brain barrier; ES: excretory/secretory; ELISA: enzyme-linked immunosorbent assay; FRbp: fatty acid and retinol-binding protein; GnRHR: gonadotropin-releasing hormone receptor; IFs: intermediate filaments; LDH: lactic acid dehydrogenase. SD: standard deviations; PBS: phosphate-buffered saline.

## Authors' contributions

HH contributed to the conception and design of the project, bioinformatics analysis and interpretation of the data, and drafted the manuscript. CM also carried out molecular studies and drafted the manuscript. YX conducted RNA isolation and cDNA library development from larval tissue. MJ contributed to cDNA library construction, candidate protein purification and interpretation of the data. PZ and GP purified candidate proteins and performed mouse immunization experiments. HA, ZX and LZ contributed to the data analysis and discussion. ZX contributed to the conception and design of the project and drafted the manuscript. All authors read and approved the final manuscript.

## Supplementary Material

Additional file 1 **Description of 168 full-length cDNAs of *A. cantonensis***. The data provided represent the statistical analysis of transcript structure, function prediction and other biological characterization of 168 full-length cDNAs. *, indicates cDNA clusters could be found among other two different cDNA librarys constructed by other researcheres. &, indicates this full-length cDNA was previously submitted by other researcheres. Click here for file

Additional file 2 **Putative secretory proteins by SignalP program**. The data provided represent the statistical analysis of signal peptide or signal anchor of putative proteins products of 168 full-length cDNAs. *, cDNA contain both transmembrane domain and signal peptide or signal anchor. Click here for file

Additional file 3 **Putative transmembrane proteins by TMHMM program**. The data provided represent the statistical analysis of transmembrane domain of putative proteins products of 168 full-length cDNAs. *, cDNA contain both transmembrane domain and signal peptide or signal anchor. Click here for file

Additional file 4 **Gene ontology (GO) classification of cDNAs**. The data provided represent the statistical analysis of Gene ontology (GO) classification of 378 cDNAs, including molecular function categories, biological process categories and cellular component categories. Click here for file

Additional file 5 **cDNA with catalytic activity was involved in metabolism**. The data provided represent the statistical analysis of catalytic activity of putative protein productes of cDNA involved in metabolism. N indicates copies of cDNA in the first batch sequencing. * indicates more than two items base on Gene ontology, and the other terms could be found in table S4. \ indicates there was no item description for cDNA based on Gene ontology. Some cDNAs factually could not be predicted, but with some others owing to not full-length of cDNA sequence. ?, Not sure what metabolism pathway related. Click here for file

Additional file 6 **Sequence alignment and conserved feature or sites of four gene products**. The data provided represent the analysis of sequence alignment and conserved feature or sites of four gene products, including Aspartic Protease, cystatin, Intermediate filaments (IFs) and Lactic acid dehydrogenase (LDH). Click here for file

Additional file 7 **Sensitivities and specificities of crude antigen, ES antigen and four candidate recombinant proteins**. The data provided represent the diagnostic analysis of four recombinant proteins. Data were derived from table 2 in text. Sensitivities and specificities analysis were referred to Intapan. TP: True Positive; TN: True Negative; FP: False Positive; FN: False Negative. Sensitivity = No. of TP/(No. of TP+No. of FN);Specificity = No. of TN/(No. of TN+No. of FP). Click here for file

Additional file 8 **Pathological changes in the brains of mice experimentally infected with *A. cantonensis***. The data provided represent the vaccine potential analysis of recombinant cystatin. Pathological changes in the brains of mouse experimentally infected with A. cantonensis (haematoxylin and eosin staining; 100×, day 21). (a) Healthy group; (b) Group vaccinated with protein of cystatin. These two groups were not infected larvae; (c) Group only vaccinated with Freund's adjuvant; (d) vaccinated with cystatin. These two groups were infected with L3 larvae after vaccinated. Red arrows signal cutting plane of larvae which were surrounded by eosinophils, inflammatory and glial cells. Click here for file
