# Supplementary material for: Preliminary molecular characterization of the human pathogen Angiostrongylus cantonensis
Source: BMC Mol Biol. 2009 Oct 25;10:97. doi: 10.1186/1471-2199-10-97 (PMC2774698; doi:10.1186/1471-2199-10-97)
Supplement: Additional file 1 — Description of 168 full-length cDNAs of A. cantonensis. The data provided represent the statistical analysis of transcript structure, function prediction and other biological characterization of 168 full-length cDNAs. *, indicates cDNA clusters could be found among other two different cDNA librarys constructed by other researcheres. &, indicates this full-length cDNA was previously submitted by other researcheres. [file 1471-2199-10-97-S1.PDF]

Additional file 1. Description of 168 full-length cDNAs of *A. cantonensis*

Clong number	Accession number	Copy	mRNA length (bp)	5'UTR length （bp）	ORF Star site	ORF Stop site	3'UTR length （bp）	Proteinlength (aa)	Description of homologous gene (with the highest E value in result of BlastX)	EST deposits relate to full-length cDNA
00010A03	FM207743	1	1254	77	78	893	361	272	Hypothetical protein Y48E1B.7 [*Caenorhabditis elegans*]	
00010A05	Unsubmitted	5	713	54	55	480	233	142	Calponin protein 3 [*Caenorhabditis elegans*]	DN190801 (Accession nunmber)
00010B04	Unsubmitted	11	500	13	14	460	40	149	immunodominant hypodermal antigen Ac16 [*Ancylostoma caninum*]	
00010B08	Unsubmitted	14	879	390	391	810	69	140	NADH dehydrogenase subunit 1 [*Ancylostoma duodenale*]	DN190405
00010B11	FM207693	13	1102	37	38	928	174	297	ADP/ATP translocator [*Trichostrongylus vitrinus*]	DN190620, DN190912, DN190908, DN191205
00010C01	FM207694	3	703	120	121	570	133	150	15 kDa selenoprotein	

									precursor, putative [*Brugia malayi*]	
00010D02	Unsubmitted	3	1025	665	666	1013	12	116	NADH dehydrogenase subunit 2 [*Ancylostoma duodenale*]	
00010D03	Unsubmitted	2	585	42	43	450	135	136	pterin-4-alpha-carbinolamine dehydratase [*Diaphorina citri*]	
00010D08	FM207695	119	941	8	9	869	72	287	Nematode cuticle collagen N-terminal domain containing protein [*Brugia malayi*]	DN191093, DN190817, DN191119, DN190603, DN191130, DN190589, DN191171, DN190517, DN191042, DN190984, DN191062, DN190212, DN190956, DN190476, DN191180, DN190168, DN190175,
00010D12	FM207744	1	674	59	60	602	72	181	Hypothetical protein	

									CBG05818 [*Caenorhabditis briggsae*]	
00010E03	FM207757	2	409	77	78	314	95	79	HMG box family protein [*Tetrahymena thermophila* SB210]	
00010E04	FM207758	5	474	42	43	342	132	100	hypothetical protein Tc00.1047053505193.50 [*Trypanosoma cruzi strain CL Brener*]	
00010E08	Unsubmitted	3	978	63	64	516	462	151	Myosin Light Chain family member (mlc-3) [*Caenorhabditis elegans*]	DN190819, CV826717
00010E10	FM207696	2	1199	41	42	917	282	292	Hypothetical protein CBG03270 [*Caenorhabditis briggsae*]	
00010E11	FM207697	2	386	26	27	215	171	63	CG13211-PA, putative [*Brugia malayi*]	
00010F02	FM207698	10	784	81	82	726	58	215	Hypothetical protein CBG13426 [*Caenorhabditis briggsae*]	DN191198, DN190977, DN191035,
00010F05	Unsubmitted	1	1217	63	64	1080	137	339	hypothetical protein [*Equus caballus*]	

00010F07	Unsubmitted	2	968	58	59	877	91	273	Prion-like-(Q/N-rich)-domain-bearing protein family member (pqn-48)[*Caenorhabditis elegans*]	
00010F09	FM207699	2	1226	82	83	952	274	290	hypothetical protein Bm1_24650 [*Brugia malayi*]	
00010G05	Unsubmitted	31	1573	837	838	1206	367	123	cytochrome oxidase subunit 1 [*Cooperia oncophora*]	
00010G08	FM207759	11	1102	316	317	436	666	40	Hypothetical protein	CV826696, CV826692, CV826678, CV826702, CV826672,
00010G09	Unsubmitted	5	1617	74	75	1466	151	464	elongation factor 1 alpha [*Dictyocaulus viviparus*]	DN190950
00010G12	FM207700	16	998	46	47	928	70	294	COLlagen family member (col-3) [*Caenorhabditis elegans*]	DN190909
00010H06	FM207701	2	1090	16	17	904	186	296	COLlagen family member (col-176) [*Caenorhabditis*	

									elegans]	
00010H07	FM207760	1	706	101	102	395	311	98	Possible species-specific gene of *Angiostrongylus cantonensis*	
00010H09	FM207702	1	821	39	40	249	572	70	Possible species-specific gene of *Angiostrongylus cantonensis*	
00010H10	FM207703	2	1028	23	24	590	438	189	Hypothetical protein CBG19427 [*Caenorhabditis briggsae*]	DN190189, DN190379
00011A04	Unsubmitted	4	420	11	12	350	70	113	Possible species-specific gene of *Angiostrongylus cantonensis*	
00011A09	Unsubmitted	6	984	57	58	918	66	287	putative collage protein 140 [*Angiostrongylus cantonensis*]	DN191232
00011A10	FM207859	1	819	47	48	722	97	225	Possible species-specific gene of *Angiostrongylus cantonensis*	
00011B03	FM207704	4	540	284	285	395	145	37	cytochrome P450 like_TBP [*Nicotiana*	

									tabacum]	
00011B07	FM207705	6	472	25	26	313	159	96	Possible species-specific gene of *Angiostrongylus cantonensis*	
00011B11	FM207706	15	1092	73	74	1060	32	329	COLlagen family member (col-65) [*Caenorhabditis elegans*]	DN190586, DN190656, DN191157, DN191155, DN190914, DN190832, DN191095, DN190687, DN190447, DN191089, DN190332, DN191010, DN190271, DN190339, DN190937, DN190350, DN190988, DN190523, DN191326, DN190896, DN190356,

										DN191249, DN190971, DN190246, DN191174, DN190179, DN190191,
00011C01	Unsubmitted	2	551	14	15	482	69	156	CalPoNin family member (cpn-4) [*Caenorhabditis elegans*]	
00011C06	FM207707	1	989	512	513	848	141	112	TCP1-chaperonin cofactor A [*Gallus gallus*]	
00011D06	Unsubmitted	6	386	16	17	343	43	109	Possible species-specific gene of *Angiostrongylus cantonensis*	
00011D09	Unsubmitted	1	693	20	21	650	43	210	Probable elongation factor 1-beta/1-delta 1 (EF-1-beta/delta 1) [*Caenorhabditis elegans*]	
00011D10	Unsubmitted	2	1132	84	85	957	175	291	PREDICTED: similar to epsilon subunit of coatomer protein complex isoform 2	

									[*Canis familiaris*]
00011F11	Unsubmitted	1	1322	303	304	957	365	218	CAP-z protein family member (cap-2) [*Caenorhabditis elegans*]
00011H03	FM207710	1	1003	297	298	687	316	130	Hypothetical protein Y62E10A [*Caenorhabditis elegans*]
00011H08	FM207711	1	1948	47	48	1175	773	376	Prion-like-(Q/N-rich)-domain-bearing protein family member (pqn-51) [*Caenorhabditis elegans*]
00011H09	Unsubmitted	1	671	15	16	543	128	176	Hypothetical protein CBG13426 [*Caenorhabditis briggsae*]
00012A04	Unsubmitted	2	1613	17	18	1523	87	502	Rho-GTPase-activating, putative [*Brugia malayi*]
00012A07	FM207858	1	1753	1134	1135	1584	182	150	Hypothetical protein W04G3.7 [*Caenorhabditis elegans*]

00012B07	FM207712	3	671	66	67	537	134	157	Heat Shock Protein family member (hsp-16.2) [Caenorhabditis elegans]	
00012B11	Unsubmitted	2	1179	31	32	1138	41	368	cysteine proteinase [Haemonchus contortus]	
00012C01	FM207745	4	305	25	26	214	91	63	DNA-binding response regulator CreB [Pseudomonas entomophila L48]	
00012C03	Unsubmitted	8	1742	17	18	1496	246	493	protein disulfide isomerase [*Ancylostoma caninum*]	
00012C04	FM207713	1	1285	61	62	889	396	276	X-box Binding Protein homolog family member (xbp-1) [*Caenorhabditis elegans*]	
00012D11	FM207860	5	982	49	50	685	297	212	Possible species-specific gene of *Angiostrongylus cantonensis*	DN190310
00012F02	FM207714	1	1436	17	18	1352	84	445	Hypothetical protein ZK1128.1	

									[*Caenorhabditis elegans*]	
00012F06	FM207715	2	1758	875	876	1373	385	166	Hypothetical protein C23H4.8 [*Caenorhabditis elegans*]	
00012F09	Unsubmitted	10	965	13	14	895	70	294	CathePsin Z family member (cpz-1) [*Caenorhabditis elegans*]	
00012F12	Unsubmitted	1	2236	154	155	1462	774	436	ADIPOR-like receptor C43G2.1 [*Caenorhabditis elegans*]	
00012G02	FM207716	2	1059	49	50	904	155	285	Thyroglobulin type-1 repeat family protein [*Brugia malayi*]	
00012G06	Unsubmitted	2	920	56	57	743	177	229	NEFA-interacting nuclear protein NIP30 [*Brugia malayi*]	
00012H06	FM207717	1	759	23	24	470	289	149	Heat Shock Protein family member (hsp-16.1) [*Caenorhabditis elegans*]	
0005A02	Unsubmitted	2	778	60	61	600	178	180	fatty acid and	DN190643,

									retinol-binding protein 1 [*Ancylostoma duodenale*]	DN190779, DN191343, DN191251, DN190746, DN190497,
0005A12	FM207673	4	524	16	17	430	94	138	Hypothetical protein F09F9.3 [*Caenorhabditis elegans*]	
0005B06	FM207780	1	1457	124	125	1429	28	435	26S proteasome regulatory chain 4, putative [*Brugia malayi*]	
0005E02	FM207674	1	1288	327	328	1251	37	308	transcription factor yin yang 2 [*Homo sapiens*]	
0005F12	Unsubmitted	4	1253	19	20	1084	169	355	cathepsin L 1 [*Dictyocaulus viviparus*]	
0005G11	FM207675	1	1170	76	77	907	263	277	Hypothetical protein B0564.11 [*Caenorhabditis elegans*]	
0005H08	FM207676	2	563	23	24	512	51	163	Lipid Binding Protein family member (lbp-1) [*Caenorhabditis elegans*]	DN190796

0005H10	Unsubmitted	1	676	49	50	595	81	182	Ribosomal protein L14p/L23e containing protein [*Brugia malayi*]
0006A10	Unsubmitted	2	1034	94	95	994	40	300	putative collage protein 140 [*Angiostrongylus cantonensis*]
0006C03	Unsubmitted	1	1422	101	102	1364	58	421	GAMMA-BBH [*Angiostrongylus cantonensis*]
0006C06	FM207677	1	1270	252	253	1134	136	294	MFP2 [*Ascaris suum*]
0006D06	Unsubmitted	1	3133	1389	1390	2925	208	512	ABC transporter family protein [*Brugia malayi*]
0006E02	FM207678	1	1138	6	7	735	403	243	conserved hypothetical protein [*Brugia malayi*]
0006E12	Unsubmitted	1	1714	358	359	1315	399	319	PTH2_CAEEL Probable peptidyl-tRNA hydrolase 2 (PTH 2) [*Caenorhabditis briggsae*]
0006F02	FM207756	1	514	52	53	274	240	74	putative organic solvent tolerance protein [*Myxococcus xanthus DK 1622*]
0006G05	FM207741	1	827	10	11	175	652	55	Hypothetical protein C09D4.1b

									[*Caenorhabditis elegans*]	
0006G07	Unsubmitted	2	986	626	627	824	162	66	Possible species-specific gene of *Angiostrongylus cantonensis*	
0006H06	FM207679	1	1289	104	105	1274	15	390	YQN2_CAEEL Uncharacterized GTP-binding protein E02H1.2 [*Caenorhabditis elegans*]	
0006H08	FM207680	1	399	8	9	344	55	112	Ribosomal Protein, Large subunit family member (rpl-34) [*Caenorhabditis elegans*] elegans]	
0007B01	FM207681	1	1725	114	115	1614	111	500	Pre-mRNA splicing protein prp5, putative [*Brugia malayi*]	DN190437
0007B02	FM207682	1	1212	53	54	1040	172	329	Growth hormone regulated TBC protein 1 [*Strongylocentrotus purpuratus*]	
0007B06	Unsubmitted	2	1196	87	88	939	257	284	tropomyosin	

									[*Heligmosomoides polygyrus*]	
0007B10	FM207683	2	742	38	39	632	110	198	Ribosomal protein 19 [*Caenorhabditis elegans*]	
0007B12	Unsubmitted	1	1335	7	8	1279	56	424	secreted-protein 1 precursor [*Ancylostoma ceylanicum*]	DN190305
0007C01	Unsubmitted	2	790	101	102	608	182	169	DeoxyUTPase family member (dut-1) [*Caenorhabditis elegans*]	
0007C05	FM207684	1	2315	123	124	1992	323	623	Hsp90 protein [*Brugia malayi*]	
0007C06	Unsubmitted	1	599	57	58	420	179	121	Vacuolar H ATPase family member (vha-9) [*Caenorhabditis elegans*]	DN190772
0007D03	Unsubmitted	1	1102	19	20	1051	51	344	cathepsin B-like cysteine protease 2 [*Parelaphostrongylus tenuis*]	
0007D04	Unsubmitted	1	1494	688	689	1339	155	217	saccharopine dehydrogenase (putative) [*Gallus gallus*]	

0007E11	FM207685	2	1014	26	27	839	175	271	Possible species-specific gene of *Angiostrongylus cantonensis*
0007F05	Unsubmitted	1	968	82	83	955	13	291	Methyltransferase-like protein, putative [*Brugia malayi*]
0007G01	Unsubmitted	1	1898	19	20	1174	724	385	Acid sphingomyelinase protein 3, isoform a [*Caenorhabditis elegans*]
0007G03	FM207686	1	1087	15	16	882	205	289	COLlagen family member (col-124) [*Caenorhabditis elegans*]
0007G04	FM207687	1	824	74	75	695	129	207	Ribosomal Protein, Large subunit family member (rpl-13) [*Caenorhabditis elegans*]
0008A11	Unsubmitted	1	1209	141	142	1149	60	338	Lactate DeHydrogenase family member (ldh-1) [Caenorhabditis elegans]
0008B08	FM207742	1	1546	69	70	645	901	192	similar to somatostatin receptor [*Nasonia*

									vitripennis]	
0008C01	FM207688	1	1318	88	89	790	528	234	Hypothetical protein CBG13393 [*Caenorhabditis briggsae*]	
0008C10	Unsubmitted	1	1438	61	62	1219	219	386	ZDHHC14 protein [*Homo sapiens*]	
0008D02	FM207689	1	1076	30	31	777	299	249	Mediator protein 6 [*Caenorhabditis elegans*]	
0009C09	FM207690	1	1217	323	324	1049	168	242	Hypothetical protein CBG09770 [*Caenorhabditis briggsae*]	
0009D08	FM207691	1	798	375	376	666	132	97	Hypothetical protein CBG12575 [*Caenorhabditis briggsae*]	
0009E02	Unsubmitted	1	1193	18	19	834	359	272	2 (Zwei) IG-domain protein family member (zig-1) [*Caenorhabditis elegans*]	DN190155
0009F10	FM207692	1	777	26	27	749	28	241	major allergen [*Brugia malayi*]	
0013A11	FM207718	2	876	11	12	854	22	281	Hypothetical protein CBG00644	

									[*Caenorhabditis briggsae*]	
0013A12	Unsubmitted	1	1517	25	26	1477	40	484	major allergen [*Brugia malayi*]	
0013C05	FM207861	1	890	513	514	726	164	71	Possible species-specific gene of *Angiostrongylus cantonensis*	
0013C10	FM207720	3	989	9	10	903	86	298	COLlagen family member (col-176) [*Caenorhabditis elegans*]	DN190678, DN190895, DN190893, DN191339, DN191333, DN191215, DN190645, DN191256, DN191101, DN191308, DN191283, DN191288, DN191273, DN190360, DN191322, DN191216, DN190722, DN191247,

										DN191290, DN190512, DN190974, DN190741, DN190757, DN190485,
0013D05	FM207719	1	1896	56	57	959	937	301	GnHR receptor homolog, putative [*Brugia malayi*]	
0013D06	FM207862	1	3499	1217	1218	1481	2018	88	Possible species-specific gene of *Angiostrongylus cantonensis*	
0013D11	Unsubmitted	1	957	85	86	568	389	161	Hypothetical protein Y37D8A.16 [*Caenorhabditis elegans*]	DN190143
0013F05	FM207863	1	733	500	501	689	44	63	sin3b [*Culex pipiens quinquefasciatus*]	
0013G04	FM207721	1	1067	21	22	897	170	292	Conserved Cystein/Glycine domain protein family member (ccg-1) [*Caenorhabditis elegans*]	
0013G05	FM207864	1	478	71	72	326	152	85	Possible species-specific gene of	DN190858, DN190664,

									Angiostrongylus cantonensis	DN190705, DN190243, DN190884, DN190335, DN190247, DN191091,
0013G06	FM207746	1	1804	63	64	1656	148	531	phage tail tape measure protein, TP901 family [*Paenibacillus larvae subsp. larvae BRL-230010*]	
0013H08	FM207722	2	835	31	32	655	180	208	Possible species-specific gene of *Angiostrongylus cantonensis*	
0014A02	FM207723	1	690	47	48	491	199	148	RNA polymerase ii (b) subunit protein 8 (RPB8) [*Caenorhabditis briggsae*]	
0014A08	FM207724	1	942	95	96	914	28	273	MAD-Like family member (mdl-1) [*Caenorhabditis elegans*]	
0014B10 &	DQ384535	136	1097	18	19	891	206	291	putative collagen protein 140[*Angiostrongylus*	DN190444, DN190307, DN190806,

									cantonensis]	DN19112, DN191115, CV826711, DN191011, DN190630, CV826691
0014D02	FM207725	1	1442	109	110	1177	265	356	C23H3.2a [*Caenorhabditis elegans*]	
0014D10	FM207865	1	955	93	94	627	328	178	Possible species-specific gene of *Angiostrongylus cantonensis*	
0014F02	Unsubmitted	2	1121	25	26	1066	55	347	cathepsin B-like cysteine protease 1 [*Parelaphostrongylus tenuis*]	
0014F09	Unsubmitted	2	671	221	222	491	180	90	Ornithine decarboxylase antizyme (ODC-Az) [*Pristionchus pacificus*]	
0014H04	Unsubmitted	1	1108	26	27	1070	38	348	N(4)-(beta-N-acetylglucosaminyl)-L-asparaginase precursor [*Caenorhabditis elegans*]	

0014H06	FM207726	1	327	11	12	257	70	82	BolA-like family protein [*Arabidopsis thaliana*]
001A06	Unsubmitted	1	1337	50	51	1154	183	368	FATty acid desaturase family member (fat-2) [*Caenorhabditis elegans*]
001A08	FM207661	4	681	27	28	474	207	149	Heat Shock Protein family member (hsp-16.1) [*Caenorhabditis elegans*]
001D12	FM207662	1	2048	48	49	1617	431	523	Synaptic vesicle 2-related protein (SV2-related protein) [*Xenopus laevis*]
001E02	FM207663	1	845	76	77	760	85	228	Hypothetical protein Y39B6A.9 [*Caenorhabditis elegans*]
001E04	Unsubmitted	1	1667	142	143	1540	127	466	Possible species-specific gene of *Angiostrongylus cantonensis*
001E07	FM207749	1	484	46	47	436	48	130	IQ calmodulin-binding motif domain protein

									[*Neosartorya fischeri NRRL 181*]
001E09	Unsubmitted	1	1067	183	184	819	248	212	Hypothetical protein F38B6.3 [*Caenorhabditis elegans*]
001H04	FM207750	1	1449	198	199	396	1053	66	Possible species-specific gene of *Angiostrongylus cantonensis*
001H12	Unsubmitted	1	1615	540	541	1146	469	202	CG30390-PA, putative [*Brugia malayi*]
002A08	FM207751	3	578	58	59	373	205	105	hypothetical protein BC1G09466 [*Botryotinia fuckeliana*]
002B12	Unsubmitted	1	1568	26	27	1241	327	405	S-adenosylmethionine synthetase [*Caenorhabditis elegans*]
002C05	Unsubmitted	1	605	295	296	598	7	101	ATPase subunit 6 [*Necator americanus*]
002C06	Unsubmitted	1	1276	6	7	1104	172	366	Fructose-bisphosphate aldolase 2 (Aldolase CE-2) (CE2) [*Caenorhabditis elegans*]

002C07	FM207664	2	1176	10	11	1090	86	360	Hypothetical protein C50F4.1 [*Caenorhabditis elegans*]	
002C10	Unsubmitted	1	1116	37	38	541	575	168	C-type LECtin family member (clec-1) [*Caenorhabditis elegans*]	
002D02	FM207665	1	1740	46	47	1156	584	370	Hypothetical protein CBG04238 [*Caenorhabditis briggsae*]	
002D11	FM207752	1	406	41	42	272	134	77	predicted protein [*Nematostella vectensis*]	
002E05	FM207730	1	1049	93	94	885	164	264	myelin transcription factor 1-like [*Bos taurus*]	DN190904
002F11	FM207731	1	2574	149	150	2231	343	694	PAP/25A associated domain containing protein [*Brugia malayi*]	
002H06	FM207753	1	702	406	407	610	92	68	Possible species-specific gene of *Angiostrongylus cantonensis*	
002H10	FM207734	1	990	11	12	710	280	233	AGAP005369-PA	

									[*Anopheles gambiae str. PEST*]	
003B04	FM207666	1	1139	23	24	842	297	273	TolA protein, putative [*Brugia malayi*]	
003B05	FM207667	1	458	76	77	271	187	65	hypothetical protein CBG09983 [*Caenorhabditis briggsae*]	DN190446
003B08	Unsubmitted	1	659	439	440	649	10	70	AMine oXidase family member (amx-3) [*Caenorhabditis elegans*]	
003B11	FM207736	1	483	18	19	432	51	138	Hypothetical protein CBG13683 [*Caenorhabditis briggsae*]	
003E02	FM207738	1	1429	698	699	1382	47	228	Hypothetical protein CBG09694 [*Caenorhabditis briggsae*]	
003F06	FM207754	1	528	8	9	422	106	138	Possible species-specific gene of *Angiostrongylus cantonensis*	
003G10	FM207732	1	1371	29	30	947	424	306	PQ loop repeat family protein [*Brugia malayi*]	DN190587

003H11	FM207668	1	1126	117	118	1059	67	314	COLlagen family member col-84 [*Caenorhabditis elegans*]
004A01	FM207669	1	819	28	29	757	62	243	Ribosomal Protein, Large subunit family member (rpl-7) [*Caenorhabditis briggsae*]
004E05	FM207670	1	566	46	47	496	70	150	Prion-like-(Q/N-rich)-domain-bearing protein family member (pqn-68) [*Caenorhabditis briggsae*]
004E10	FM207671	1	1054	497	498	800	254	101	Hypothetical protein F52A8.6a [*Caenorhabditis elegans*]
004E12	Unsubmitted	1	1459	38	39	1349	110	437	HomoGentisate Oxidase family member (hgo-1) [*Caenorhabditis elegans*]
004G07	Unsubmitted	2	720	213	214	603	117	130	NADH Ubiquinone Oxidoreductase family member (nuo-3) [*Caenorhabditis

									elegans]
004H11	FM207672	1	927	213	214	690	237	159	Unidentified Vitellogenin-linked Transcript family member (uvt-2) [*Caenorhabditis briggsae*]
15A07	FM207747	1	1060	44	45	818	242	258	unknown protein [*Caenorhabditis elegans*]
15D10	FM207866	2	400	111	112	378	22	89	Possible species-specific gene of *Angiostrongylus cantonensis*
15E08	Unsubmitted	1	1241	8	9	1193	48	395	4-HydroxyPhenylpyruvate Dioxygenase (HPD) family member (hpd-1) [*Caenorhabditis elegans*]
15E12	FM207867	1	263	3	4	183	80	60	Possible species-specific gene of *Angiostrongylus cantonensis*
15G10	Unsubmitted	1	1420	170	171	1109	311	313	ribokinase [*Desulfatibacillum alkenivorans AK-01*]

15G12	Unsubmitted	1	1433	62	63	1247	186	395	ASpartyl Protease family member (asp-1) [*Caenorhabditis elegans*]
15H03	FM207868	1	1055	243	244	900	155	219	Possible species-specific gene of *Angiostrongylus cantonensis*
16B08	FM207748	1	1345	1044	1045	1257	88	71	Hypothetical protein CBG14815 [*Caenorhabditis briggsae*]
16B10	Unsubmitted	14	1446	37	38	1378	68	446	cathepsin D-like aspartic protease[Ancylostoma ceylanicum]
16C04	FM207727	1	904	204	205	807	97	201	Hypothetical protein Y105C5A.8 [*Caenorhabditis elegans*]
16D08	FM207869	1	1368	520	521	682	686	54	Possible species-specific gene of *Angiostrongylus cantonensis*
16E03	FM207728	1	1633	123	124	855	778	244	NSF attachment protein, putative

16H04	FM207729	1	1077	18	19	486	591	156	[*Brugia malayi*] Hypothetical protein R05C11.4 [*Caenorhabditis elegans*]

*, indicates cDNA clusters could be found among other two different cDNA librarys constructed by other researcheres. &, indicates this full-length cDNA was previously submitted by other researcheres.
