# Supplementary material for: Preliminary molecular characterization of the human pathogen Angiostrongylus cantonensis
Source: BMC Mol Biol. 2009 Oct 25;10:97. doi: 10.1186/1471-2199-10-97 (PMC2774698; doi:10.1186/1471-2199-10-97)
Supplement: Additional file 2 — Putative secretory proteins by SignalP program. The data provided represent the statistical analysis of signal peptide or signal anchor of putative proteins products of 168 full-length cDNAs. *, cDNA contain both transmembrane domain and signal peptide or signal anchor. [file 1471-2199-10-97-S2.PDF]

Additional file 2. Putative secretory proteins by SignalP program

Clone number	Accession number	Homology gene	Length of protein (AA)	SignalP-neutral networks		SignalP-hidden Markov model		
				Mean S	Cleavage site	Prediction	Signal peptide probability	Max cleavage site probability
00010B04	Unsubmitted	immunodominant hypodermal antigen Ac16 [*Ancylostoma caninum*]	149	1-19 0.886	19 and 20: VEG-QS	Signal peptide	1.000	0.913 between pos. 19 and 20
00010C01	FM207694	15 kDa selenoprotein precursor, putative [*Brugia malayi*]	150	1-15 0.768	15 and 16: SYA-EI	Signal peptide	0.975	0.974 between pos. 15 and 16
00010D02*	Unsubmitted	NADH dehydrogenase subunit 2 [*Ancylostoma duodenale*]	116	1-29 0.823	29 and 30: NEG-LV	Signal anchor	0.995	0.001 between pos. 20 and 21
00010D03	Unsubmitted	Putative pterin-4-alpha-carbinolamine dehydratase [*Diaphorina citri*]	136	1-19 0.801	19 and 20: SHG-ID	Signal peptide	0.995	0.963 between pos. 19 and 20
00010D08*	FM207695	Nematode cuticle collagen	287	1-35 0.451	35 and 36: IIA-IP	Signal anchor	0.944	0.007 between pos. 35 and 36

		N-terminal domain containing protein [*Brugia malayi*]						
00010E04	FM207758	hypothetical protein Tc00.1047053505193.50 [*Trypanosoma cruzi*]	100	1-20 0.963	20 and 21: TDG-QY	Signal peptide	1.000	0.938 between pos. 20 and 21
00010E11	FM207697	CG13211-PA, putative [*Brugia malayi*]	63	1-33 0.411	33 and 34: GFC-SY	Signal anchor	0.948	0.007 between pos. 33 and 34
00010F02	FM207698	Hypothetical protein CBG13426 [*Caenorhabditis briggsae*]	215	1-16 0.979	16 and 17: VIA-AP	Signal peptide	1.000	0.713 between pos. 16 and 17
00010F07	Unsubmitted	Prion-like-(Q/N-rich)-do main-bearing protein family member (pqn-48) [*Caenorhabditis briggsae*]	273	1-19 0.678	19 and 20: TLA-IH	Signal peptide	0.869	0.857 between pos. 19 and 20
00010G12*	FM207700	COLlagen family member (col-3) [*Caenorhabditis elegans*]	294	1-29 0.652	29 and 30: VLS-VC	Signal anchor	0.915	0.043 between pos. 33 and 34
00010H06*	FM207701	COLlagen family member (col-176) [*Caenorhabditis*	296	1-22 0.844	22 and 23: SAC-IV	Signal peptide	0.557	0.252 between pos. 22 and 23

		elegans]						
00010H09*	FM207702	Hypothetical protein T07A9.15 [*Caenorhabditis elegans*]	70	1-33 0.741	33 and 34: RTA-GM	Signal anchor	0.627	0.114 between pos. 28 and 29
00011A04	Unsubmitted	major surface glycoprotein [*Pneumocystis carinii f. sp. carinii*]	113	1-23 0.949	23 and 24: TLG-WS	Signal peptide	0.998	0.687 between pos. 23 and 24
00011A09*	Unsubmitted	putative collagen 140 [*Angiostrongylus cantonensis*]	287	1-18 0.972	18 and 19: VAC-LV	Signal peptide	0.648	0.283 between pos. 18 and 19
00011B03	FM207704	cytochrome P450 like_TBP [*Nicotiana tabacum*]	225	1-25 0.699	25 and 26: QNG-RT	Signal peptide	0.723	0.297 between pos. 19 and 20
00011B07	FM207705	Hypothetical protein CBG03916 [*Caenorhabditis briggsae*]	96	1-15 0.750	15 and 16: VYA-AP	Signal peptide	0.998	0.964 between pos. 15 and 16
00011B11*	FM207706	COLlagen family member (col-65) [*Caenorhabditis elegans*]	329	1-28 0.785	28 and 29: AYL-VN	Signal anchor	0.691	0.147 between pos. 21 and 22

00011D06	Unsubmitted	Pyruvate:ferredoxin oxidoreductase, delta subunit [*Methanopyrus kandleri AV19*]	109	1-23 0.829	23 and 24: TFG-MP	Signal peptide	0.986	0.911 between pos. 23 and 24
00011H03	FM207710	Hypothetical protein Y62E10A.11 [Caenorhabditis elegans]	130	1-21 0.137	17 and 18: TCC-TT	Signal anchor	0.582	0.049 between pos. 21 and 22
00012B11	Unsubmitted	cysteine proteinase [Haemonchus contortus]	368	1-23 0.514	23 and 24: CAA-AT	Signal peptide	0.978	0.388 between pos. 61 and 62
00012C01	FM207745	DNA-binding response regulator CreB [*Pseudomonas entomophila L48*]	63	1-21 0.943	21 and 22: TVA-NP	Signal peptide	1.000	0.968 between pos. 21 and 22
00012C03	Unsubmitted	protein disulfide isomerase [*Ancylostoma caninum*]	493	1-16 0.937	16 and 17: GFA-AD	Signal peptide	1.000	0.901 between pos. 16 and 17
00012D11	FM207860	hypothetical protein 003-32 [*Oikopleura dioica*]	212	1-19 0.853	19 and 20: TNC-MN	Signal peptide	0.946	0.815 between pos. 19 and 20
00012F09	Unsubmitted	CathePsin Z family member (cpz-1)	294	1-17 0.933	17 and 18: VSA-AN	Signal peptide	0.996	0.760 between pos. 17 and 18

		[*Caenorhabditis elegans*]						
00012G02	FM207716	Thyroglobulin type-1 repeat family protein [*Brugia malayi*]	285	1-18 0.976	18 and 19: TLA-SS	Signal peptide	0.999	0.457 between pos. 20 and 21
0005A02	Unsubmitted	Fatty acid and retinol-binding protein 1 [*Ancylostoma duodenale*]	180	1-17 0.945	17 and 18: ASA-AP	Signal peptide	0.999	0.885 between pos. 17 and 18
0005A12	FM207673	Hypothetical protein F09F9.3 [*Caenorhabditis elegans*]	138	1-19 0.931	19 and 20: ISA-SP	Signal peptide	0.998	0.826 between pos. 19 and 20
0005F12	Unsubmitted	cathepsin L 1 [*Dictyocaulus viviparus*]	355	1-17 0.887	17 and 18: TWT-NF	Signal peptide	0.998	0.975 between pos. 17 and 18
0005H08	Unsubmitted	Lipid Binding Protein family member (lbp-1) [*Caenorhabditis elegans*]	163	1-18 0.816	18 and 19: IHA-KE	Signal peptide	0.997	0.931 between pos. 18 and 19
0006A10*	Unsubmitted	putative collage protein 140 [*Angiostrongylus cantonensis*]	300	1-33 0.758	33 and 34: TIG-AV	Signal anchor	0.908	0.055 between pos. 33 and 34
0006G05*	FM207741	Hypothetical protein	55	1-24 0.842	24 and 25: GLT-AL	Signal peptide	0.748	0.321 between pos. 25 and 26

		C09D4.1b [*Caenorhabditis elegans*]						
0006G07*	Unsubmitted	PREDICTED: similar to PRED65 [*Pan troglodytes*]	66	1-21 0.830	21 and 22: VLA-YV	Signal peptide	0.772	0.294 between pos. 26 and 27
0007B12	Unsubmitted	secreted-protein 1 precursor [*Ancylostoma ceylanicum*]	424	1-19 0.924	19 and 20: VVA-DD	Signal peptide	0.994	0.952 between pos. 19 and 20
0007D03	Unsubmitted	cathepsin B-like cysteine protease 2 [*Parelaphostrongylus tenuis*]	344	1-17 0.936	17 and 18: VSA-VP	Signal peptide	1.000	0.976 between pos. 17 and 18
0007G01	Unsubmitted	Acid SphingoMyelinase family member (asm-3) [*Caenorhabditis elegans*]	385	1-17 0.952	17 and 18: ALA-SH	Signal peptide	1.000	0.906 between pos. 17 and 18
0007G03*	FM207686	COLlagen family member (col-124) [*Caenorhabditis elegans*]	289	1-30 0.621	30 and 31: CAT-LP	Signal anchor	0.976	0.008 between pos. 30 and 31
0008B08*	FM207742	PREDICTED: similar to somatostatin receptor,	192	1-23 0.816	23 and 24: LLC-SV	Signal anchor	0.367	0.051 between pos. 35 and 36

		putative [*Nasonia vitripennis*]						
0008C01*	FM207688	Hypothetical protein CBG13393 [*Caenorhabditis briggsae*]	234	1-25 0.721	25 and 26: SHG-FT	Signal peptide	0.969	0.685 between pos. 25 and 26
0009E02	Unsubmitted	2 (Zwei) IG-domain protein family member (zig-1) [*Caenorhabditis elegans*]	272	1-17 0.976	17 and 18: VSA-LT	Signal peptide	1.000	0.758 between pos. 17 and 18
0009F10	FM207692	Hypothetical protein ZC412.3 [*Caenorhabditis elegans*]	241	1-15 0.776	15 and 16: ALA-CE	Signal peptide	0.968	0.900 between pos. 15 and 16
0013A12	Unsubmitted	Hypothetical protein ZC412.3 [*Caenorhabditis elegans*]	484	1-15 0.848	15 and 16: ALA-CI	Signal peptide	0.914	0.797 between pos. 15 and 16
0013C05*	FM207861	UHRF2 protein [Ciona intestinalis]	71	1-27 0.831	27 and 28: NKA-LY	Signal peptide	0.943	0.243 between pos. 27 and 28
0013C10*	FM207720	COLlagen family member (col-176) [*Caenorhabditis elegans*]	298	1-28 0.732	28 and 29: VPS-LY	Signal anchor	0.737	0.169 between pos. 22 and 23

0013D11*	Unsubmitted	Hypothetical protein Y37D8A.16 [*Caenorhabditis elegans*]	161	1-35 0.875	35 and 36: VTA-YI	Signal peptide	0.597	0.582 between pos. 35 and 36
0013F05	FM207863	sin3b [*Culex pipiens quinquefasciatus*]	63	1-16 0.804	16 and 17: CRC-CC	Signal anchor	0.951	0.008 between pos. 49 and 50
0013G04	FM207721	Conserved Cystein/Glycine domain protein family member (ccg-1) [*Caenorhabditis elegans*]	292	1-19 0.952	19 and 20: VEG-AA	Signal peptide	1.000	0.609 between pos. 19 and 20
0013G06*	FM207746	Phage tail tape measure protein, TP901 family [*Paenibacillus larvae subsp. larvae BRL-230010*]	531	1-24 0.717	24 and 25: VCS-ME	Signal peptide	0.912	0.441 between pos. 24 and 25
0014B10	Unsubmitted	Putative collagen 140 [*Angiostrongylus cantonensis*]	291	1-33 0.583	33 and 34: VPQ-DH	Signal peptide	0.765	0.495 between pos. 33 and 34
0014F02	Unsubmitted	cathepsin B-like cysteine protease 1 [*Parelaphostrongylus*	346	1-16 0.857	16 and 17: VSA-AS	Signal peptide	1.000	0.838 between pos. 16 and 17

		tenuis]						
001E02	FM207663	Hypothetical protein Y39B6A.9 [*Caenorhabditis elegans*]	228	1-17 0.922	17 and 18: TSS-GY	Signal peptide	0.999	0.506 between pos. 18 and 19
001E07	FM207749	IQ calmodulin-binding motif domain protein [*Neosartorya fischeri NRRL 181*]	130	1-18 0.682	18 and 19: AFA-AK	Signal peptide	0.964	0.923 between pos. 18 and 19
001E09	Unsubmitted	Hypothetical protein F38B6.3 [*Caenorhabditis elegans*]	212	1-61 0.202	61 and 62: VIA-II	Signal anchor	1.000	0.000 between pos. 61 and 62
002C10	Unsubmitted	C-type LECtin family member (clec-1) [*Caenorhabditis elegans*]	168	1-18 0.911	18 and 19: AYA-AS	Signal peptide	1.000	0.904 between pos. 18 and 19
002H10	FM207734	AGAP005369-PA [*Anopheles gambiae str. PEST*]	233	1-17 0.966	17 and 18: ALS-GL	Signal peptide	0.999	0.635 between pos. 17 and 18
003B08	Unsubmitted	Hypothetical protein F55C5.6 [*Caenorhabditis elegans*]	70	1-21 0.547	21 and 22: SAA-AV	Signal peptide	0.766	0.481 between pos. 21 and 22

003G10*	FM207732	PQ loop repeat family protein [*Brugia malayi*]	306	1-58<br>0.157	/	Signal anchor	0.758	0.001 between pos. 15 and 16
003H11*	FM207668	COLlagen family member (col-84) [*Caenorhabditis elegans*]	314	1-27<br>0.719	27 and 28: IGQ-IY	Signal peptide	0.929	0.666 between pos. 26 and 27
15G12	Unsubmitted	ASpartyl Protease family member (asp-1) [*Caenorhabditis elegans*]	395	1-16<br>0.854	16 and 17: VYS-KT	Signal peptide	0.997	0.933 between pos. 16 and 17
16B10	Unsubmitted	Cathepsin D-like aspartic protease [*Ancylostoma ceylanicum*]	446	1-31<br>0.566	31 and 32: CAC-AG	Signal peptide	0.991	0.189 between pos. 31 and 32
16H04	FM207729	Hypothetical protein R05C11.4 [*Caenorhabditis elegans*]	156	1-31<br>0.714	31 and 32: AGG-LP	Signal peptide	0.987	0.600 between pos. 21 and 22

*, cDNA contain both transmembrane domain and signal peptide or signal anchor
