# Supplementary material for: Preliminary molecular characterization of the human pathogen Angiostrongylus cantonensis
Source: BMC Mol Biol. 2009 Oct 25;10:97. doi: 10.1186/1471-2199-10-97 (PMC2774698; doi:10.1186/1471-2199-10-97)
Supplement: Additional file 3 — Putative transmembrane proteins by TMHMM program. The data provided represent the statistical analysis of transmembrane domain of putative proteins products of 168 full-length cDNAs. *, cDNA contain both transmembrane domain and signal peptide or signal anchor. [file 1471-2199-10-97-S3.PDF]

Additional file 3. Putative transmembrane proteins by TMHMM program

Clone number	Accession number	Description of homology gene	Length of protein (AA)	SignalP	Number of Predicted TMH	Position of TM helix
00010B08	Unsubmitted	NADH dehydrogenase subunit 1 [*Ancylostoma duodenale*]	140	/	4	12-34; 39-58; 79-101; 105-124
00010B11	FM207693	ADP/ATP translocator [*Trichostrongylus vitrinus*]	297	/	3	114-136; 177-196; 211-230
00010D02*	Unsubmitted	NADH dehydrogenase subunit 2 [*Ancylostoma duodenale*]	116	Signal anchor	4	4-22; 29-51; 66-88; 95-114
00010D08*	FM207695	Nematode cuticle collagen N-terminal domain containing protein [*Brugia malayi*]	287	Signal anchor	1	17-39
00010E11	FM207697	CG13211-PA, putative [*Brugia malayi*]	63	Signal anchor	1	19-41
00010G05	Unsubmitted	cytochrome c oxidase subunit I	123	/	3	32-54; 64-86; 99-121

		[*Cooperia oncophora*]				
00010G12*	FM207700	COLlagen family member (col-3) [*Caenorhabditis elegans*]	294	Signal anchor	1	20-42
00010H06*	Unsubmitted	COLlagen family member (col-176) [*Caenorhabditis elegans*]	296	Signal peptide	1	07-29
00010H09*	Unsubmitted	Hypothetical protein T07A9.15 [*Caenorhabditis elegans*]	70	Signal anchor	1	13-35
00011A09*	Unsubmitted	putative collagen 140 [*Angiostrongylus cantonensis*]	287	Signal peptide	1	04-26
00011B11*	FM207706	Hypothetical protein CBG06230 [*Caenorhabditis briggsae*]	329	Signal anchor	1	07-29
00012F06	FM207715	Hypothetical protein C23H4.8 [*Caenorhabditis elegans*]	166	/	1	106-128
00012F12	Unsubmitted	ADIPOR-like receptor C43G2.1, putative [*Brugia malayi*]	436	/	7	194-216; 229-251; 266-286; 293-312; 322-344; 356-375; 390-412
0006A10*	Unsubmitted	Cuticle collagen C09G5.5, putative [*Brugia malayi*]	300	Signal anchor	1	12-34

0006G05*	FM207741	Hypothetical protein C09D4.1b [*Caenorhabditis elegans*]	55	Signal peptide	1	04-26
0006G07*	Unsubmitted	PREDICTED: similar to PRED65 [*Pan troglodytes*]	66	Signal peptide	1	20-42
0007D04	Unsubmitted	Hypothetical protein CBG17873 [*Caenorhabditis briggsae*]	217	/	1	63-85
0007G03*	FM207686	Hypothetical protein T05A1.2 [*Caenorhabditis elegans*]	289	Signal anchor	1	13-35
0008B08*	FM207742	PREDICTED: similar to somatostatin receptor, putative [*Nasonia vitripennis*]	192	Signal anchor	4	15-37; 50-72; 99-121; 150-167
0008C01*	FM207688	Hypothetical protein CBG13393 [*Caenorhabditis briggsae*]	234	Signal peptide	7	4-21; 34-56; 76-98; 111-133; 138-160; 173-195; 205-227
0008C10	Unsubmitted	ZDHHC14 protein [*Homo sapiens*]	386	/	4	86-108; 118-135; 243-265; 280-302
0009D08	FM207691	Hypothetical protein CBG12575 [*Caenorhabditis briggsae*]	97	/	1	13-32

0009E02	Unsubmitted	2 (Zwei) IG-domain protein family member (zig-1) [*Caenorhabditis elegans*]	272	Signal peptide	1	232-254
0013A11	FM207718	Hypothetical protein CBG00644 [*Caenorhabditis briggsae*]	281	/	3	20-42; 73-90; 103-125
0013C05*	FM207861	UHRF2 protein [*Ciona intestinalis*]	71	Signal peptide	2	9-31; 36-58
0013C10*	FM207720	Hypothetical protein CBG01972 [*Caenorhabditis briggsae*]	298	Signal anchor	1	07-29
0013D05	FM207719	GnHR receptor homolog, putative *[Brugia malayi]*	301	/	5	10-32; 45-63; 97-119; 160-182; 195-214
0013D11*	Unsubmitted	Hypothetical protein Y37D8A.16 [*Caenorhabditis elegans*]	161	Signal peptide	4	7-29; 44-66; 79-101; 116-138
0013G06*	FM207746	phage tail tape measure protein, TP901 family [*Paenibacillus larvae subsp. larvae BRL-230010*]	531	Signal peptide	1	503-525
0014D02	FM207725	Hypothetical protein C23H3.2a [*Caenorhabditis elegans*]	356	/	4	71-93; 155-177; 190-212; 278-300

001A06	Unsubmitted	FATty acid desaturase family member (fat-2) [*Caenorhabditis elegans*]	368	/	4	37-59; 64-86; 200-222; 227-245
001D12	FM207662	Synaptic vesicle 2-related protein [*Xenopus laevis*]<br>ZK637.1 [*Caenorhabditis elegans*]	523	/	12	89-111; 121-143; 150-172; 177-199; 212-234; 239-261; 325-347; 378-400; 405-422; 426-448; 461-483; 493-512
001E09	Unsubmitted	Hypothetical protein F38B6.3 [*Caenorhabditis elegans*]	212	Signal anchor	1	46-68
001H04	FM207750	Large exoprotein<br>[*Roseovarius sp. TM1035*]	66	/	1	21-43
002C05	Unsubmitted	ATPase subunit 6<br>[*Necator americanus*]	101	/	2	38-60; 70-92
002D02	FM207665	Hypothetical protein CBG04238 *[Caenorhabditis briggsae*]	370	/	6	67-89; 102-121; 126-148; 234-251; 266-288; 309-331
003E02	FM207738	Hypothetical protein CBG09694 [*Caenorhabditis briggsae*]	228	/	1	199-221

003G10*	FM207732	PQ loop repeat family protein [*Brugia malayi*]	306	Signal anchor	6	39-61; 76-95; 102-121; 185-207; 227-249; 259-281
003H11*	FM207668	Hypothetical protein K12D12.3 [*Caenorhabditis elegans*]	314	Signal peptide	1	07-29

*, cDNA contain both transmembrane domain and signal peptide or signal anchor
