# Supplementary material for: Preliminary molecular characterization of the human pathogen Angiostrongylus cantonensis
Source: BMC Mol Biol. 2009 Oct 25;10:97. doi: 10.1186/1471-2199-10-97 (PMC2774698; doi:10.1186/1471-2199-10-97)
Supplement: Additional file 4 — Gene ontology (GO) classification of cDNAs. The data provided represent the statistical analysis of Gene ontology (GO) classification of 378 cDNAs, including molecular function categories, biological process categories and cellular component categories. [file 1471-2199-10-97-S4.PDF]

Additional file 4. Gene ontology (GO) classification of *A. cantonensis* cDNAs.

- **Additional file 4-1 Term distribution of molecular function categories**
- **Additional file 4-2 Term distribution of biological process categories**
- **Additional file 4-3 Term distribution of cellular component categories**
- **Additional file 4-4 GO classification of 378 gene clusters based on molecular function**
- **Additional file 4-5 GO classification of 378 gene clusters based on biological process**
- **Additional file 4-6 GO classification of 378 gene clusters based on cellular component**

Additional file 4-1. Distribution of molecular function categories

Definition of main category term	Main category ID	Count	Distribution
binding	GO:0005488	121	41.9%
catalytic activity	GO:0003824	81	28.0%
structural molecule activity	GO:0005198	29	10.0%
transporter activity	GO:0005215	17	5.9%
translation regulator activity	GO:0045182	11	3.8%
enzyme regulator activity	GO:0030234	10	3.5%
transcription regulator activity	GO:0030528	10	3.5%
molecular transducer activity	GO:0060089	6	2.1%
motor activity	GO:0003774	3	1.0%
metallochaperone activity	GO:0016530	1	0.3%

Additional file 4-2. Distribution of biological process categories

Definition of main category term	Main category ID	Count	Distribution
cellular process	GO:0009987	128	24.8%
metabolic process	GO:0008152	94	18.2%
developmental process	GO:0032502	57	11.0%
multicellular organismal process	GO:0032501	51	9.9%
biological regulation	GO:0065007	51	9.9%
localization	GO:0051179	50	9.7%
reproduction	GO:0000003	32	6.2%
growth	GO:0040007	26	5.0%
response to stimulus	GO:0050896	21	4.1%
immune system process	GO:0002376	4	0.8%
biological adhesion	GO:0022610	1	0.2%
locomotion	GO:0040011	1	0.2%
multi-organism process	GO:0051704	1	0.2%

Additional file 4-3. Distribution of cellular component categories

Definition of main category term	Main category ID	Count	Distribution
cell	GO:0005623	155	51.8%
organelle	GO:0043226	90	30.1%
macromolecular complex	GO:0032991	33	11.0%
extracellular region	GO:0005576	12	4.0%
membrane-enclosed lumen	GO:0031974	4	1.3%
synapse	GO:0045202	3	1.0%
envelope	GO:0031975	2	0.7%

Additional file 4-4. GO classification of 378 gene clusters based on molecular function

Clone NO.	Subcategory ID.	Definition of Subcategory term	Main category
00010A08	GO:0003743	translation initiation factor activity	GO:0045182 and GO:0005488
	GO:0005525	GTP binding	GO:0005488
00010A09	GO:0004194	pepsin A activity	GO:0003824
	GO:0005515	protein binding	GO:0005488
00010A12	GO:0003723	RNA binding	GO:0005488
	GO:0003735	structural constituent of ribosome	GO:0005198
00010B02	GO:0004707	MAP kinase activity	GO:0003824
	GO:0005524	ATP binding	GO:0005488
00010B03	GO:0004674	protein serine and threonine kinase activity	GO:0003824
	GO:0004712	protein threonine and tyrosine kinase activity	GO:0003824
	GO:0005515	protein binding	GO:0005488
	GO:0005524	ATP binding	GO:0005488
00010B05	GO:0004504	peptidylglycine monooxygenase activity	GO:0003824
	GO:0004598	peptidylamidoglycolate lyase activity	GO:0003824
	GO:0005507	copper ion binding	GO:0005488
	GO:0008270	zinc ion binding	GO:0005488
00010B06	GO:0051015	actin filament binding	GO:0005488
00010B08	GO:0008137	NADH dehydrogenase (ubiquinone) activity	GO:0003824
00010B11	GO:0005215	transporter activity	GO:0005215
00010B11	GO:0005488	binding	GO:0005488
00010B12	GO:0004129	cytochrome-c oxidase activity	GO:0003824

	GO:0005507	copper ion binding	GO:0005488
	GO:0020037	heme binding	GO:0005488
00010C01	GO:0005515	protein binding	GO:0005488
	GO:0008430	selenium binding	GO:0005488
00010C11	GO:0005506	iron ion binding	GO:0005488
	GO:0016491	oxidoreductase activity	GO:0003824
00010D02	GO:0008137	NADH dehydrogenase (ubiquinone) activity	GO:0003824
00010D03	GO:0008124	4-alpha-hydroxytetrahydrobiopterin dehydratase activity	GO:0003824
00010D06	GO:0005515	protein binding	GO:0005488
	GO:0016651	oxidoreductase activity, acting on NADH and NADPH	GO:0003824
00010D11	GO:0005089	Rho guanyl-nucleotide exchange factor activity	GO:0030234
	GO:0008017	microtubule binding	GO:0005488
	GO:0008270	zinc ion binding	GO:0005488
	GO:0017048	Rho GTPase binding	GO:0005488
	GO:0019992	diacylglycerol binding	GO:0005488
	GO:0030676	Rac guanyl-nucleotide exchange factor activity	GO:0030234
	GO:0048365	Rac GTPase binding	GO:0005488
00010D12	GO:0042302	structural constituent of cuticle	GO:0005198
00010E06	GO:0003682	chromatin binding	GO:0005488
	GO:0005515	protein binding	GO:0005488
	GO:0005524	ATP binding	GO:0005488
	GO:0008094	DNA-dependent ATPase activity	GO:0003824
00010E06	GO:0008270	zinc ion binding	GO:0005488
00010E08	GO:0003774	motor activity	GO:0003774
	GO:0005509	calcium ion binding	GO:0005488

00010E12	GO:0015078	hydrogen ion transporter activity	GO:0005215
	GO:0015175	neutral amino acid transporter activity	GO:0005215
	GO:0015293	symporter activity	GO:0005215
00010F01	GO:0004322	ferroxidase activity	GO:0003824
	GO:0008199	ferric iron binding	GO:0005488
00010F04	GO:0003743	translation initiation factor activity	GO:0045182 and GO:0005488
	GO:0003924	GTPase activity	GO:0003824
	GO:0005525	GTP binding	GO:0005488
	GO:0043024	ribosomal small subunit binding	GO:0005488
00010F05	GO:0005515	protein binding	GO:0005488
00010F07	GO:0016491	oxidoreductase activity	GO:0003824
00010F09	GO:0005515	protein binding	GO:0005488
	GO:0008270	zinc ion binding	GO:0005488
00010G05	GO:0004129	cytochrome-c oxidase activity	GO:0003824
	GO:0005506	iron ion binding	GO:0005488
	GO:0005507	copper ion binding	GO:0005488
	GO:0020037	heme binding	GO:0005488
00010G09	GO:0003746	translation elongation factor activity	GO:0045182 and GO:0005488
	GO:0003924	GTPase activity	GO:0003824
	GO:0005525	GTP binding	GO:0005488
00010G12	GO:0042302	structural constituent of cuticle	GO:0005198
00010H06	GO:0042302	structural constituent of cuticle	GO:0005198
00010H12	GO:0003774	motor activity	GO:0003774
	GO:0003779	actin binding	GO:0005488
	GO:0005516	calmodulin binding	GO:0005488

	GO:0005524	ATP binding	GO:0005488
00011A09	GO:0042302	structural constituent of cuticle	GO:0005198
00011B11	GO:0042302	structural constituent of cuticle	GO:0005198
00011C01	GO:0003779	actin binding	GO:0005488
	GO:0005516	calmodulin binding	GO:0005488
00011C06	GO:0051082	unfolded protein binding	GO:0005488
00011D09	GO:0003746	translation elongation factor activity	GO:0045182 and GO:0005488
00011D10	GO:0005198	structural molecule activity	GO:0005198
	GO:0005515	protein binding	GO:0005488
	GO:0008565	protein transporter activity	GO:0005215
00011E02	GO:0017089	glycolipid transporter activity	GO:0005215
00011F11	GO:0051015	actin filament binding	GO:0005488
00011H03	GO:0005085	guanyl-nucleotide exchange factor activity	GO:0030234
	GO:0005515	protein binding	GO:0005488
	GO:0008270	zinc ion binding	GO:0005488
00011H07	GO:0008470	isovaleryl-CoA dehydrogenase activity	GO:0003824
	GO:0050660	FAD binding	GO:0005488
00011H08	GO:0003677	DNA binding	GO:0005488
	GO:0003702	RNA polymerase II transcription factor activity	GO:0005488 and GO:0030528
	GO:0003713	transcription coactivator activity	GO:0005488 and GO:0030528
00011H10	GO:0003746	translation elongation factor activity	GO:0045182 and GO:0005488
	GO:0005515	protein binding	GO:0005488
00012A04	GO:0005096	GTPase activator activity	GO:0030234
00012A05	GO:0003746	translation elongation factor activity	GO:0045182 and GO:0005488
	GO:0003924	GTPase activity	GO:0003824

	GO:0005525	GTP binding	GO:0005488
00012A06	GO:0005515	protein binding	GO:0005488
	GO:0008565	protein transporter activity	GO:0005215
00012B06	GO:0005515	protein binding	GO:0005488
	GO:0005525	GTP binding	GO:0005488
00012C03	GO:0003756	protein disulfide isomerase activity	GO:0003824
	GO:0003810	protein-glutamine gamma-glutamyltransferase activity	GO:0003824
00012C04	GO:0003700	transcription factor activity	GO:0005488 and GO:0030528
	GO:0046983	protein dimerization activity	GO:0005488
00012C06	GO:0005524	ATP binding	GO:0005488
	GO:0042802	protein self binding	GO:0005488
	GO:0051082	unfolded protein binding	GO:0005488
00012C11	GO:0005529	sugar binding	GO:0005488
00012D12	GO:0003677	DNA binding	GO:0005488
00012E06	GO:0005524	ATP binding	GO:0005488
	GO:0051082	unfolded protein binding	GO:0005488
00012E12	GO:0005261	cation channel activity	GO:0005215
00012F02	GO:0008168	methyltransferase activity	GO:0003824
00012F04	GO:0003677	DNA binding	GO:0005488
	GO:0008270	zinc ion binding	GO:0005488
00012F09	GO:0004197	cysteine-type endopeptidase activity	GO:0003824
00012F12	GO:0004872	receptor activity	GO:0060089
	GO:0042562	hormone binding	GO:0005488
00012G02	GO:0005509	calcium ion binding	GO:0005488
00012G03	GO:0016491	oxidoreductase activity	GO:0003824

00012G04	GO:0004674	protein serine and threonine kinase activity	GO:0003824
	GO:0005516	calmodulin binding	GO:0005488
	GO:0005524	ATP binding	GO:0005488
	GO:0008307	structural constituent of muscle	GO:0005198
	GO:0017022	myosin binding	GO:0005488
	GO:0042802	protein self binding	GO:0005488
00012G05	GO:0003735	structural constituent of ribosome	GO:0005198
	GO:0005515	protein binding	GO:0005488
	GO:0019843	rRNA binding	GO:0005488
00012G09	GO:0004129	cytochrome-c oxidase activity	GO:0003824 and G0:0005215
00012H04	GO:0005524	ATP binding	GO:0005488
00012H05	GO:0005086	ARF guanyl-nucleotide exchange factor activity	GO:0030234
	GO:0005515	protein binding	GO:0005488
0005A02	GO:0008289	lipid binding	GO:0005488
0005A09	GO:0005515	protein binding	GO:0005488
0005A11	GO:0008233	peptidase activity	GO:0003824
0005B06	GO:0005515	protein binding	GO:0005488
	GO:0005524	ATP binding	GO:0005488
	GO:0017111	nucleoside-triphosphatase activity	GO:0003824
0005C03	GO:0000287	magnesium ion binding	GO:0005488
	GO:0004550	nucleoside-diphosphate kinase activity	GO:0003824
	GO:0005524	ATP binding	GO:0005488
0005C09	GO:0003883	CTP synthase activity	GO:0003824
0005E02	GO:0003676	nucleic acid binding	GO:0005488
	GO:0008270	zinc ion binding	GO:0005488

0005E11	GO:0005198	structural molecule activity	GO:0005198
0005F12	GO:0004185	Carboxypeptidase activity	GO:0003824
	GO:0004217	cathepsin L activity	GO:0003824
0005G12	GO:0008137	NADH dehydrogenase (ubiquinone) activity	GO:0003824
0005H08	GO:0005215	transporter activity	GO:0005215
	GO:0005515	protein binding	GO:0005488
	GO:0008289	lipid binding	GO:0005488
0005H10	GO:0003735	structural constituent of ribosome	GO:0005198
0005H11	GO:0003700	transcription factor activity	GO:0005488 and GO:0030528
0006A10	GO:0042302	structural constituent of cuticle	GO:0005198
0006C03	GO:0005506	iron ion binding	GO:0005488
	GO:0008336	gamma-butyrobetaine dioxygenase activity	GO:0003824
	GO:0016702	oxidoreductase activity, acting on single donors with incorporation of molecular oxygen, incorporation of two atoms of oxygen	GO:0003824
0006D06	GO:0005524	ATP binding	GO:0005488
	GO:0016887	ATPase activity	GO:0003824
0006E12	GO:0004045	aminoacyl-tRNA hydrolase activity	GO:0003824
0006G04	GO:0004194	pepsin A activity	GO:0003824
	GO:0005515	protein binding	GO:0005488
0006G05	GO:0005215	transporter activity	GO:0005215
0006H06	GO:0003723	RNA binding	GO:0005488
	GO:0005525	GTP binding	GO:0005488
0006H08	GO:0003735	structural constituent of ribosome	GO:0005198
0007B01	GO:0005515	protein binding	GO:0005488
0007B02	GO:0005097	Rab GTPase activator activity	GO:0030234

0007B06	GO:0003779	actin binding	GO:0005488
0007B08	GO:0016787	hydrolase activity	GO:0003824
0007B10	GO:0003735	structural constituent of ribosome	GO:0005198
0007C01	GO:0000287	magnesium ion binding	GO:0005488
	GO:0004170	dUTP diphosphatase activity	GO:0003824
0007C04	GO:0003743	translation initiation factor activity	GO:0045182 and GO:0005488
0007C05	GO:0005524	ATP binding	GO:0005488
	GO:0051082	unfolded protein binding	GO:0005488
0007C06	GO:0046872	metal ion binding	GO:0005488
	GO:0046933	hydrogen-transporting ATP synthase activity, rotational mechanism	GO:0005215
0007D03	GO:0004197	cysteine-type endopeptidase activity	GO:0003824
0007D04	GO:0047131	saccharopine dehydrogenase (NAD+, L-glutamate-forming) activity	GO:0003824
0007F05	GO:0005515	protein binding	GO:0005488
	GO:0008168	methyltransferase activity	GO:0003824
0007G01	GO:0004767	sphingomyelin phosphodiesterase activity	GO:0003824
	GO:0016798	hydrolase activity, acting on glycosyl bonds	GO:0003824
0007G03	GO:0042302	structural constituent of cuticle	GO:0005198
0007G04	GO:0003735	structural constituent of ribosome	GO:0005198
0007H10	GO:0004576	oligosaccharyl transferase activity	GO:0003824
0008A07	GO:0003723	RNA binding	GO:0005488
0008A07	GO:0016439	tRNA-pseudouridine synthase activity	GO:0003824
0008B12	GO:0004871	signal transducer activity	GO:0060089
0008C06	GO:0008137	NADH dehydrogenase (ubiquinone) activity	GO:0003824
0008C10	GO:0008270	zinc ion binding	GO:0005488
	GO:0008415	acyltransferase activity	GO:0003824

0008D02	GO:0030528	transcription regulator activity	GO:0030528
0008H03	GO:0042302	structural constituent of cuticle	GO:0005198
	GO:0004182	carboxypeptidase A activity	GO:0003824
	GO:0008270	zinc ion binding	GO:0005488
	GO:0050425	carboxypeptidase B activity	GO:0030234
0009A05	GO:0005102	receptor binding	GO:0005488
	GO:0005515	protein binding	GO:0005488
0009A08	GO:0004842	ubiquitin-protein ligase activity	GO:0003824
	GO:0005515	protein binding	GO:0005488
	GO:0008270	zinc ion binding	GO:0005488
0009C09	GO:0005515	protein binding	GO:0005488
0009C10	GO:0016491	oxidoreductase activity	GO:0003824
0009D02	GO:0003777	microtubule motor activity	GO:0003774
	GO:0005524	ATP binding	GO:0005488
	GO:0016887	ATPase activity	GO:0003824
0009D10	GO:0004252	serine-type endopeptidase activity	GO:0003824
0009E02	GO:0005021	vascular endothelial growth factor receptor activity	GO:0060089 and GO:0003824
	GO:0005089	Rho guanyl-nucleotide exchange factor activity	GO:0030234
	GO:0005524	ATP binding	GO:0005488
0009E05	GO:0004674	protein serine and threonine kinase activity	GO:0003824
	GO:0005524	ATP binding	GO:0005488
0009G01	GO:0005515	protein binding	GO:0005488
0009G02	GO:0004105	choline-phosphate cytidylyltransferase activity	GO:0003824
0013A11	GO:0003824	catalytic activity	GO:0003824
0013C10	GO:0042302	structural constituent of cuticle	GO:0005198

0013C11	GO:0004198	calpain activity	GO:0003824
0013D04	GO:0005524	ATP binding	GO:0005488
	GO:0051082	unfolded protein binding	GO:0005488
0013D05	GO:0004968	gonadotropin-releasing hormone receptor activity	GO:0060089
0013E03	GO:0005515	protein binding	GO:0005488
0013F06	GO:0005515	protein binding	GO:0005488
0013F08	GO:0003677	DNA binding	GO:0005488
	GO:0004386	helicase activity	GO:0003824
	GO:0005524	ATP binding	GO:0005488
	GO:0016787	hydrolase activity	GO:0003824
0014A02	GO:0003899	DNA-directed RNA polymerase activity	GO:0003824
	GO:0005515	protein binding	GO:0005488
0014A08	GO:0003677	DNA binding	GO:0005488
	GO:0005515	protein binding	GO:0005488
	GO:0030528	transcription regulator activity	GO:0030528
0014B10	GO:0042302	structural constituent of cuticle	GO:0005198
0014C05	GO:0003746	translation elongation factor activity	GO:0045182 and GO:0005488
	GO:0003924	GTPase activity	GO:0003824
	GO:0005525	GTP binding	GO:0005488
0014E04	GO:0003723	RNA binding	GO:0005488
0014F02	GO:0004197	cysteine-type endopeptidase activity	GO:0003824
0014F09	GO:0008073	ornithine decarboxylase inhibitor activity	GO:0030234
0014G11	GO:0003676	nucleic acid binding	GO:0005488
	GO:0004527	exonuclease activity	GO:0003824
	GO:0030145	manganese ion binding	GO:0005488

0014H02	GO:0003700	transcription factor activity	GO:0005488 and GO:0030528
	GO:0046983	protein dimerization activity	GO:0005488
0014H04	GO:0003948	N4-(beta-N-acetylglucosaminyl)-L-asparaginase activity	GO:0003824
0014H05	GO:0005515	protein binding	GO:0005488
0014H06	GO:0005515	protein binding	GO:0005488
0014H09	GO:0003723	RNA binding	GO:0005488
	GO:0003743	translation initiation factor activity	GO:0045182 and GO:0005488
	GO:0005515	protein binding	GO:0005488
001A06	GO:0016717	oxidoreductase activity, acting on paired donors, with oxidation of a pair of donors resulting in the reduction of molecular oxygen to two molecules of water	GO:0003824
001B06	GO:0005215	transporter activity	GO:0005215
	GO:0005509	calcium ion binding	GO:0005488
001B08	GO:0005215	transporter activity	GO:0005215
001C02	GO:0015020	glucuronosyltransferase activity	GO:0003824
001D12	GO:0015075	ion transporter activity	GO:0005215
001F09	GO:0003756	protein disulfide isomerase activity	GO:0003824
	GO:0003810	protein-glutamine gamma-glutamyltransferase activity	GO:0003824
002B12	GO:0000287	magnesium ion binding	GO:0005488
	GO:0004478	methionine adenosyltransferase activity	GO:0003824
	GO:0005524	ATP binding	GO:0005488
	GO:0030955	potassium ion binding	GO:0005488
	GO:0050897	cobalt ion binding	GO:0005488
002C05	GO:0015078	hydrogen ion transporter activity	GO:0005215
	GO:0016820	hydrolase activity, acting on acid anhydrides, catalyzing	GO:0003824

		transmembrane movement of substances	
002C06	GO:0004332	fructose-bisphosphate aldolase activity	GO:0003824
	GO:0042802	protein self binding	GO:0005488
002C10	GO:0004872	receptor activity	GO:0060089
	GO:0005529	sugar binding	GO:0005488
002D06	GO:0005507	copper ion binding	GO:0005488
	GO:0016531	copper chaperone activity	GO:0016530 and GO:0005488
002F08	GO:0005198	structural molecule activity	GO:0005198
	GO:0005515	protein binding	GO:0005488
	GO:0005524	ATP binding	GO:0005488
002F11	GO:0003676	nucleic acid binding	GO:0005488
	GO:0008270	zinc ion binding	GO:0005488
	GO:0016779	nucleotidyltransferase activity	GO:0003824
002G08	GO:0003924	GTPase activity	GO:0003824
	GO:0005198	structural molecule activity	GO:0005198
	GO:0005525	GTP binding	GO:0005488
002H08	GO:0003746	translation elongation factor activity	GO:0045182 and GO:0005488
	GO:0003924	GTPase activity	GO:0003824
	GO:0005525	GTP binding	GO:0005488
003A10	GO:0005198	structural molecule activity	GO:0005198
	GO:0005515	protein binding	GO:0005488
	GO:0005524	ATP binding	GO:0005488
003B01	GO:0004691	cAMP-dependent protein kinase activity	GO:0003824
	GO:0005524	ATP binding	GO:0005488
003C04	GO:0016853	isomerase activity	GO:0003824

003C05	GO:0003735	structural constituent of ribosome	GO:0005198
	GO:0005515	protein binding	GO:0005488
003E03	GO:0005509	calcium ion binding	GO:0005488
003H11	GO:0042302	structural constituent of cuticle	GO:0005198
004A01	GO:0003723	RNA binding	GO:0005488
	GO:0003735	structural constituent of ribosome	GO:0005198
	GO:0005515	protein binding	GO:0005488
	GO:0030528	transcription regulator activity	GO:0030528
004A08	GO:0003924	GTPase activity	GO:0003824
	GO:0005198	structural molecule activity	GO:0005198
	GO:0005525	GTP binding	GO:0005488
004B03	GO:0000158	protein phosphatase type 2A activity	GO:0003824
	GO:0005509	calcium ion binding	GO:0005488
	GO:0005515	protein binding	GO:0005488
	GO:0008601	protein phosphatase type 2A regulator activity	GO:0030234
004E05	GO:0016788	hydrolase activity, acting on ester bonds	GO:0003824
004E10	GO:0005525	GTP binding	GO:0005488
004E12	GO:0004411	homogentisate 1,2-dioxygenase activity	GO:0003824
	GO:0005506	iron ion binding	GO:0005488
	GO:0042802	protein self binding	GO:0005488
004G04	GO:0003700	transcription factor activity	GO:0005488 and GO:0030528
	GO:0005515	protein binding	GO:0005488
	GO:0008270	zinc ion binding	GO:0005488
004H04	GO:0005215	transporter activity	GO:0005215
	GO:0005509	calcium ion binding	GO:0005488

004H11	GO:0005509	calcium ion binding	GO:0005488
15A01	GO:0003700	transcription factor activity	GO:0005488 and GO:0030528
	GO:0003702	RNA polymerase II transcription factor activity	GO:0005488 and GO:0030528
	GO:0008134	transcription factor binding	GO:0005488
	GO:0046983	protein dimerization activity	GO:0005488
15D01	GO:0005515	protein binding	GO:0005488
	GO:0008565	protein transporter activity	GO:0005215
15D11	GO:0005198	structural molecule activity	GO:0005198
	GO:0005515	protein binding	GO:0005488
	GO:0005524	ATP binding	GO:0005488
15E07	GO:0005198	structural molecule activity	GO:0005198
	GO:0005515	protein binding	GO:0005488
	GO:0008565	protein transporter activity	GO:0005215
15E08	GO:0003868	4-hydroxyphenylpyruvate dioxygenase activity	GO:0003824
	GO:0005506	iron ion binding	GO:0005488
15E10	GO:0003735	structural constituent of ribosome	GO:0005198
	GO:0005515	protein binding	GO:0005488
15F05	GO:0008137	NADH dehydrogenase (ubiquinone) activity	GO:0003824
15G10	GO:0004747	ribokinase activity	GO:0003824
15G12	GO:0004194	pepsin A activity	GO:0003824
	GO:0005515	protein binding	GO:0005488
16A06	GO:0005515	protein binding	GO:0005488
16B10	GO:0004192	cathepsin D activity	GO:0003824
	GO:0004194	pepsin A activity	GO:0003824
16C07	GO:0005515	protein binding	GO:0005488

	GO:0008565	protein transporter activity	GO:0005215
16E03	GO:0005488	binding	GO:0005488
16E06	GO:0005125	cytokine activity	GO:0005488
16F10	GO:0005515	protein binding	GO:0005488
	GO:0008270	zinc ion binding	GO:0005488
16G08	GO:0003674	molecular_function	GO:0003674
Total (185)	Total (348)	Total (135)	

Additional file 4-5. Classification of 378 gene clusters based on biological process

Clone NO.	Subcategory ID.	Definition of Subcategory term	Main category
00010A05	GO:0007517	muscle development	GO:0032501 and GO:0032502
00010A08	GO:0006446	regulation of translational initiation	GO:0009987 and GO:0065007 and GO:0008152 and GO:0043993 and GO:0050789
00010A09	GO:0006508	proteolysis and peptidolysis	GO:0009987 and GO:0008152
	GO:0008219	cell death	GO:0009987 and GO:0032502
00010A12	GO:0006412	protein biosynthesis	GO:0009987 and GO:0008152
00010B02	GO:0006468	protein amino acid phosphorylation	GO:0009987 and GO:0008152
	GO:0007254	JNK cascade	GO:0009987 and GO:0065007 and GO:0050789
00010B03	GO:0006468	protein amino acid phosphorylation	GO:0009987 and GO:0008152
	GO:0007094	mitotic spindle checkpoint	GO:0065007 and GO:0009987 and GO:0050789
	GO:0007103	spindle pole body duplication in nuclear envelope	GO:0009987
	GO:0051225	spindle assembly	GO:0009987
00010B05	GO:0006518	peptide metabolism	GO:0009987 and GO:0008152
00010B06	GO:0000578	embryonic axis specification	GO:0032502 and GO:0032501
	GO:0002119	larval development (sensu Nematoda)	GO:0032502 and GO:0032501
	GO:0009792	embryonic development (sensu Metazoa)	GO:0032501 and GO:0032502 and GO:0000003 and GO:0022414
	GO:0035046	pronuclear migration	GO:0009987 and GO:0051234 and GO:0051179 and GO:0000003 and GO:0022414

	GO:0040010	positive regulation of growth rate	GO:0065007 and GO:0040007 and GO:0048518 and GO:0050789
	GO:0040018	positive regulation of body size	GO:0065007 and GO:0040007 and GO:0032501 and GO:0048518 and GO:0050789
00010B06	GO:0040032	post-embryonic body morphogenesis	GO:0032501 and GO:0032502
	GO:0051016	barbed-end actin filament capping	GO:0065007 and GO:0009987 and GO:0043933 and GO:0008152
00010B08	GO:0006120	mitochondrial electron transport, NADH to ubiquinone	GO:0009987 and GO:0008152
00010B11	GO:0006810	transport	GO:0051234 and GO:0051179
00010B12	GO:0006118	electron transport	GO:0051234 and GO:0051179
	GO:0006810	transport	GO:0051234 and GO:0051179
00010C11	GO:0006118	electron transport	GO:0051234 and GO:0051179
	GO:0006810	transport	GO:0051234 and GO:0051179
00010D06	GO:0045087	innate immune response	GO:0002376 and GO:0050896
	GO:0051341	regulation of oxidoreductase activity	GO:0065007
00010D11	GO:0000902	cellular morphogenesis	GO:0009987 and GO:0032502
	GO:0007015	actin filament organization	GO:0009987
	GO:0007026	negative regulation of microtubule depolymerization	GO:0065007 and GO:0009877 and GO:0043933 and GO:0008152 and GO:0048519 and GO:0050789
	GO:0035023	regulation of Rho protein signal transduction	GO:0009987 and GO:0065007 and GO:0050789
00010D12	GO:0006817	phosphate transport	GO:0051234 and GO:0051179
	GO:0007626	locomotory behavior	GO:0050896
	GO:0040002	cuticle biosynthesis (sensu Nematoda)	GO:0032502 and GO:0032501

	GO:0040018	positive regulation of body size	GO:0065007 and GO:0040007 and GO:0032501 and GO:0048518 and GO:0050789
00010D12	GO:0040032	post-embryonic body morphogenesis	GO:0032501 and GO:0032502
00010E06	GO:0007049	cell cycle	GO:0009987
	GO:0030174	regulation of DNA replication initiation	GO:0065007 and GO:0009877 and GO:0008152 and GO:0050789
00010E09	GO:0016559	peroxisome division	GO:0009987
00010E12	GO:0015804	neutral amino acid transport	GO:0051234 and GO:0051179
	GO:0015992	proton transport	GO:0051234 and GO:0051179
00010F01	GO:0006826	iron ion transport	GO:0051234 and GO:0051179
	GO:0006879	iron ion homeostasis	GO:0065007 and GO:0009987
00010F04	GO:0006446	regulation of translational initiation	GO:0009987 and GO:0065007 and GO:0008152 and GO:0043993 and GO:0050789
00010F07	GO:0019886	antigen processing, exogenous antigen via MHC class II	GO:0002376
00010G05	GO:0006123	mitochondrial electron transport, cytochrome c to oxygen	GO:0009987 and GO:0008152
	GO:0006810	transport	GO:0051234 and GO:0051179
	GO:0009060	aerobic respiration	GO:0009987 and GO:0008152
00010G09	GO:0002119	larval development (sensu Nematoda)	GO:0032502 and GO:0032501
	GO:0006414	translational elongation	GO:0009987 and GO:0008152
	GO:0007276	gametogenesis	GO:0000003
	GO:0009792	embryonic development (sensu Metazoa)	GO:0032501 and GO:0032502 and GO:0000003 and GO:0022414
	GO:0040007	growth	GO:0040007

00010G12	GO:0006817	phosphate transport	GO:0051234 and GO:0051179
	GO:0007626	locomotory behavior	GO:0050896
	GO:0040002	cuticle biosynthesis (sensu Nematoda)	GO:0032502 and GO:0032501
	GO:0040018	positive regulation of body size	GO:0065007 and GO:0040007 and GO:0032501 and GO:0048518 and GO:0050789
	GO:0040032	post-embryonic body morphogenesis	GO:0032501 and GO:0032502
00010H06	GO:0006817	phosphate transport	GO:0051234 and GO:0051179
00010H10	GO:0016192	vesicle-mediated transport	GO:0051234 and GO:0051179 and GO:0009987
00010H12	GO:0006605	protein targeting	GO:0009987 and GO:0051234 and GO:0051179
	GO:0006897	endocytosis	GO:0051234 and GO:0051179 and GO:0009987
	GO:0007268	synaptic transmission	GO:0009987 and GO:0032501
	GO:0007605	perception of sound	GO:0032501
	GO:0007626	locomotory behavior	GO:0050896
	GO:0016358	dendrite morphogenesis	GO:0009987 and GO:0032502 and GO:0032501
	GO:0042472	inner ear morphogenesis	GO:0032501 and GO:0032502
	GO:0042491	auditory hair cell differentiation	GO:0032501 and GO:0032502 and GO:0009987
	GO:0048167	regulation of synaptic plasticity	GO:0065007 and GO:0050789 and GO:0032501 and GO:0009987
00011A08	GO:0030150	mitochondrial matrix protein import	GO:0009987 and GO:0051234 and GO:0051179
00011A09	GO:0002009	morphogenesis of an epithelium	GO:0032502
	GO:0006817	phosphate transport	GO:0051234 and GO:0051179
	GO:0040018	positive regulation of body size	GO:0065007 and GO:0040007 and GO:0032501 and GO:0048518 and GO:0050789
00011B11	GO:0006817	phosphate transport	GO:0051234 and GO:0051179
00011C01	GO:0031032	actomyosin structure organization and	GO:0009987

		biogenesis	
00011C06	GO:0007021	tubulin folding	GO:0009987 and GO:0043933
00011D09	GO:0002119	larval development (sensu Nematoda)	GO:0032502 and GO:0032501
	GO:0006414	translational elongation	GO:0009987 and GO:0008152
	GO:0009792	embryonic development (sensu Metazoa)	GO:0032501 and GO:0032502 and GO:0000003 and GO:0022414
	GO:0040010	positive regulation of growth rate	GO:0065007 and GO:0040007 and GO:0048518 and GO:0050789
00011D10	GO:0006461	protein complex assembly	GO:0009987 and GO:0043933
	GO:0006890	retrograde transport, Golgi to ER	GO:0051234 and GO:0051179 and GO:0009987
	GO:0006891	intra-Golgi transport	GO:0051234 and GO:0051179 and GO:0009987
	GO:0015031	protein transport	GO:0051234 and GO:0051179
00011E02	GO:0046836	glycolipid transport	GO:0051234 and GO:0051179
00011F11	GO:0000578	embryonic axis specification	GO:0032502 and GO:0032501
	GO:0002119	larval development (sensu Nematoda)	GO:0032502 and GO:0032501
	GO:0009792	embryonic development (sensu Metazoa)	GO:0032501 and GO:0032502 and GO:0000003 and GO:0022414
	GO:0035046	pronuclear migration	GO:0009987 and GO:0051234 and GO:0051179 and GO:0000003 and GO:0022414
	GO:0040010	positive regulation of growth rate	GO:0065007 and GO:0040007 and GO:0048518 and GO:50789
	GO:0040018	positive regulation of body size	GO:0065007 and GO:0040007 and GO:0032501 and GO:0048518 and GO:0050789
	GO:0040032	post-embryonic body morphogenesis	GO:0032501 and GO:0032502
	GO:0051016	barbed-end actin filament capping	GO:0065007 and GO:0009987 and GO:0043933 and GO:0008152

00011H03	GO:0007264	small GTPase mediated signal transduction	GO:0009987 and GO:0065007 and GO:0050789
	GO:0015031	protein transport	GO:0051234 and GO:0051179
00011H07	GO:0006118	electron transport	GO:0051234 and GO:0051179
00011H08	GO:0006355	regulation of transcription, DNA-dependent	GO:0065007 and GO:0009987 and GO:0008152 and GO:0050789
	GO:0006367	transcription initiation from Pol II promoter	GO:0009987 and GO:0008152 and GO:0043933
00011H10	GO:0006414	translational elongation	GO:0009987 and GO:0008152 and
00012A04	GO:0007165	signal transduction	GO:0009987 and GO:0065007 and GO:0050789
00012A05	GO:0002119	larval development (sensu Nematoda)	GO:0032502 and GO:0032501
	GO:0007276	gametogenesis	GO:0000003
	GO:0009792	embryonic development (sensu Metazoa)	GO:0032501 and GO:0032502 and GO:0000003 and GO:0022414
	GO:0040007	growth	GO:0040007
	GO:0006414	translational elongation	GO:0009987 and GO:0008152 and
00012A06	GO:0006836	neurotransmitter transport	GO:0051234 and GO:0051179
	GO:0006886	intracellular protein transport	GO:0051234 and GO:0051179 and GO:0009987
	GO:0016081	synaptic vesicle docking during exocytosis	GO:0051234 and GO:0051179 and GO:0009987 and GO:0065007 and GO:0032501
00012B06	GO:0042254	ribosome biogenesis and assembly	GO:0009987
00012B07	GO:0006950	response to stress	GO:0050896
	GO:0008340	determination of adult life span	GO:0032501 and GO:0032502
00012C03	GO:0045454	cell redox homeostasis	GO:0065007 and GO:0009987 and GO:0050789
00012C04	GO:0006355	regulation of transcription,	GO:0065007 and GO:0009987 and GO:0008152 and

		DNA-dependent	GO:0050789
00012C06	GO:0000003	reproduction	GO:0000003
	GO:0006457	protein folding	GO:0009987 and GO:0008152 and
	GO:0006950	response to stress	GO:0050896
	GO:0006952	defense response	GO:0050896
	GO:0007049	cell cycle	GO:0009987
	GO:0040024	dauer larval development (sensu Nematoda)	GO:0032501 and GO:0032502
00012D12	GO:0006334	nucleosome assembly	GO:0009987 and GO:0043933
00012E06	GO:0000003	reproduction	GO:0000003
	GO:0006457	protein folding	GO:0009987 and GO:0008152 and
	GO:0009792	embryonic development (sensu Metazoa)	GO:0032501 and GO:0032502 and GO:0000003 and GO:0022414
00012E12	GO:0006812	cation transport	GO:0051234 and GO:0051179
	GO:0007067	mitosis	GO:0009987
	GO:0008406	gonad development	GO:0032501 and GO:0032502 and GO:0000003 and GO:0022414
00012F04	GO:0007275	development	GO:0032501 and GO:0032502
00012F09	GO:0006508	proteolysis and peptidolysis	GO:0009987 and GO:0008152 and
00012F12	GO:0006355	regulation of transcription, DNA-dependent	GO:0065007 and GO:0009987 and GO:0008152 and GO:0050789
	GO:0009755	hormone-mediated signaling	GO:0065007 and GO:0009987 and GO:0050789 and GO:0050896
	GO:0019395	fatty acid oxidation	GO:0009987 and GO:0008152
00012G02	GO:0007416	synaptogenesis	GO:0032501 and GO:0032502 and GO:0009987

	GO:0045595	regulation of cell differentiation	GO:0065007 and GO:0009987 and GO:0050789 and GO:0032502
00012G03	GO:0008152	metabolism	GO:0008152
00012G04	GO:0006941	striated muscle contraction	GO:0032501
	GO:0007067	mitosis	GO:0009987
	GO:0030239	myofibril assembly	GO:0009987 and GO:0032502 and GO:0032501
	GO:0046777	autophosphorylation	GO:0009987 and GO:0008152
00012G05	GO:0002119	larval development (sensu Nematoda)	GO:0032502 and GO:0032501
	GO:0006412	protein biosynthesis	GO:0009987 and GO:0008152 and
	GO:0007276	gametogenesis	GO:0000003
	GO:0007626	locomotory behavior	GO:0050896
	GO:0009792	embryonic development (sensu Metazoa)	GO:0032501 and GO:0032502 and GO:0000003 and GO:0022414
	GO:0040010	positive regulation of growth rate	GO:0065007 and GO:0040007 and GO:0048518 and GO:50789
00012G09	GO:0006118	electron transport	GO:0051234 and GO:0051179
00012H04	GO:0006950	response to stress	GO:0050896
00012H06	GO:0006950	response to stress	GO:0050896
00012H06	GO:0006952	defense response	GO:0050896
	GO:0008340	determination of adult life span	GO:0032501 and GO:0032502
0005A02	GO:0040018	positive regulation of body size	GO:0065007 and GO:0040007 and GO:0032501 and GO:0048518 and GO:0050789
	GO:0040032	post-embryonic body morphogenesis	GO:0032501 and GO:0032502
0005A11	GO:0007219	Notch signaling pathway	GO:0009987 and GO:0065007 and GO:0050789
	GO:0007242	intracellular signaling cascade	GO:0009987 and GO:0065007 and GO:0050789

0005B06	GO:0030163	protein catabolism	GO:0008152
0005C03	GO:0006183	GTP biosynthesis	GO:0009987 and GO:0008152 and
	GO:0006228	UTP biosynthesis	GO:0009987 and GO:0008152 and
	GO:0006241	CTP biosynthesis	GO:0009987 and GO:0008152 and
0005C09	GO:0006221	pyrimidine nucleotide biosynthesis	GO:0009987 and GO:0008152 and
	GO:0006541	glutamine metabolism	GO:0009987 and GO:0008152 and
0005E12	GO:0006508	proteolysis and peptidolysis	GO:0009987 and GO:0008152 and
0005F05	GO:0009792	embryonic development (sensu Metazoa)	GO:0032501 and GO:0032502 and GO:0000003 and GO:0022414
0005F12	GO:0006508	proteolysis and peptidolysis	GO:0009987 and GO:0008152 and
	GO:0030154	cell differentiation	GO:0009987 and GO:0032502
0005G12	GO:0042773	ATP synthesis coupled electron transport	GO:0009987 and GO:0008152
0005H08	GO:0006810	transport	GO:0051234 and GO:0051179
0005H10	GO:0006412	protein biosynthesis	GO:0009987 and GO:0008152 and
0005H11	GO:0002119	larval development (sensu Nematoda)	GO:0032502 and GO:0032501
	GO:0006355	regulation of transcription, DNA-dependent	GO:0065007 and GO:0009987 and GO:0008152 and GO:0050789
	GO:0007626	locomotory behavior	GO:0050896
	GO:0009790	embryonic development	GO:0032501 and GO:0032502
	GO:0016337	cell-cell adhesion	GO:0022610 and GO:0009987
	GO:0040018	positive regulation of body size	GO:0065007 and GO:0040007 and GO:0032501 and GO:0048518 and GO:0050789
	GO:0040032	post-embryonic body morphogenesis	GO:0032501 and GO:0032502
0006A10	GO:0006817	phosphate transport	GO:0051234 and GO:0051179
	GO:0040002	cuticle biosynthesis (sensu Nematoda)	GO:0032502 and GO:0032501

0006C03	GO:0006118	electron transport	GO:0051234 and GO:0051179
	GO:0045329	carnitine biosynthesis	GO:0009987 and GO:0008152
0006E12	GO:0018987	osmoregulation	GO:0065007
	GO:0040010	positive regulation of growth rate	GO:0065007 and GO:0040007 and GO:0048518 and GO:50789
0006G04	GO:0006508	proteolysis and peptidolysis	GO:0009987 and GO:0008152 and
	GO:0008219	cell death	GO:0009987 and GO:0032502
0006G05	GO:0006810	transport	GO:0051234 and GO:0051179
0006H06	GO:0007264	small GTPase mediated signal transduction	GO:0009987 and GO:0065007 and GO:0050789
0006H08	GO:0006412	protein biosynthesis	GO:0009987 and GO:0008152 and
0007B01	GO:0006397	mRNA processing	GO:0009987 and GO:0008152 and
	GO:0008380	RNA splicing	GO:0009987 and GO:0008152
0007B08	GO:0006631	fatty acid metabolism	GO:0009987 and GO:0008152 and
0007B10	GO:0000003	reproduction	GO:0000003
	GO:0002119	larval development (sensu Nematoda)	GO:0032502 and GO:0032501
	GO:0006412	protein biosynthesis	GO:0009987 and GO:0008152 and
	GO:0009792	embryonic development (sensu Metazoa)	GO:0032501 and GO:0032502 and GO:0000003 and GO:0022414
	GO:0040007	growth	GO:0040007
0007C01	GO:0046080	dUTP metabolism	GO:0009987 and GO:0008152
0007C04	GO:0006417	regulation of protein biosynthesis	GO:0009987 and GO:0065007 and GO:0008152 and GO:0050789
0007C05	GO:0006457	protein folding	GO:0009987 and GO:0008152 and
0007C06	GO:0000003	reproduction	GO:0000003

	GO:0002119	larval development (sensu Nematoda)	GO:0032502 and GO:0032501
	GO:0007626	locomotory behavior	GO:0050896
	GO:0009792	embryonic development (sensu Metazoa)	GO:0032501 and GO:0032502 and GO:0000003 and GO:0022414
	GO:0015986	ATP synthesis coupled proton transport	GO:0009987 and GO:0051234 and GO:0051179 and GO:0008152
	GO:0040007	growth	GO:0040007
0007C08	GO:0006355	regulation of transcription, DNA-dependent	GO:0065007 and GO:0009987 and GO:0008152 and GO:0050789
0007D03	GO:0006508	proteolysis and peptidolysis	GO:0009987 and GO:0008152 and
0007G01	GO:0006685	sphingomyelin catabolism	GO:0009987 and GO:0008152 and
	GO:0046513	ceramide biosynthesis	GO:0009987 and GO:0008152
0007G03	GO:0006817	phosphate transport	GO:0051234 and GO:0051179
	GO:0007626	locomotory behavior	GO:0050896
	GO:0040002	cuticle biosynthesis (sensu Nematoda)	GO:0032502 and GO:0032501
	GO:0040018	positive regulation of body size	GO:0065007 and GO:0040007 and GO:0032501 and GO:0048518 and GO:0050789
	GO:0040032	post-embryonic body morphogenesis	GO:0032501 and GO:0032502
0007G04	GO:0006412	protein biosynthesis	GO:0009987 and GO:0008152 and
0007H10	GO:0000003	reproduction	GO:0000003
	GO:0002119	larval development (sensu Nematoda)	GO:0032502 and GO:0032501
	GO:0006486	protein amino acid glycosylation	GO:0009987 and GO:0008152 and
	GO:0007626	locomotory behavior	GO:0050896
	GO:0009792	embryonic development (sensu Metazoa)	GO:0032501 and GO:0032502 and GO:0000003 and GO:0022414

	GO:0040015	negative regulation of body size	GO:0065007 and GO:0040007 and GO:0032501 and GO:0048519 and GO:0050789
0008A07	GO:0002119	larval development (sensu Nematoda)	GO:0032502 and GO:0032501
	GO:0006364	rRNA processing	GO:0009987 and GO:0008152 and
	GO:0007276	gametogenesis	GO:0000003
	GO:0031119	tRNA pseudouridine synthesis	GO:0009987 and GO:0008152
	GO:0040010	positive regulation of growth rate	GO:0065007 and GO:0040007 and GO:0048518 and GO:50789
0008B12	GO:0007223	frizzled-2 signaling pathway	GO:0009987 and GO:0065007 and GO:0050789
	GO:0007275	development	GO:0032501 and GO:0032502
0008C06	GO:0006120	mitochondrial electron transport, NADH to ubiquinone	GO:0009987 and GO:0008152 and
0008D02	GO:0006355	regulation of transcription, DNA-dependent	GO:0065007 and GO:0009987 and GO:0008152 and GO:0050789
	GO:0008406	gonad development	GO:0032501 and GO:0032502 and GO:0000003 and GO:0022414
	GO:0009792	embryonic development (sensu Metazoa)	GO:0032501 and GO:0032502 and GO:0000003 and GO:0022414
0008E07	GO:0006464	protein modification	GO:0009987 and GO:0008152 and
0008F03	GO:0006817	phosphate transport	GO:0051234 and GO:0051179
0008H03	GO:0006508	proteolysis and peptidolysis	GO:0009987 and GO:0008152 and
0009A05	GO:0006886	intracellular protein transport	GO:0051234 and GO:0051179 and GO:0009987
	GO:0006915	apoptosis	GO:0009987 and GO:0032502
	GO:0006955	immune response	GO:0050896 and GO:0002376
	GO:0016192	vesicle-mediated transport	GO:0051234 and GO:0051179 and GO:0009987

0009A08	GO:0000209	protein polyubiquitination	GO:0009987 and GO:0008152 and
	GO:0006511	ubiquitin-dependent protein catabolism	GO:0009987 and GO:0008152 and
	GO:0007067	mitosis	GO:0009987
	GO:0051301	cell division	GO:0009987
0009B11	GO:0006464	protein modification	GO:0009987 and GO:0008152 and
0009C09	GO:0008150	biological_process	GO:0008150
0009C10	GO:0008152	metabolism	GO:0008152
0009D02	GO:0000910	cytokinesis	GO:0009987
	GO:0007018	microtubule-based movement	GO:0051234 and GO:0051179 and GO:0009987
	GO:0007051	spindle organization and biogenesis	GO:0009987
	GO:0035046	pronuclear migration	GO:0009987 and GO:0051234 and GO:0051179 and GO:0000003 and GO:0022414
	GO:0040016	embryonic cleavage	GO:0009987 and GO:0032501 and GO:0032502
0009D10	GO:0006508	proteolysis and peptidolysis	GO:0009987 and GO:0008152 and
0009E02	GO:0006468	protein amino acid phosphorylation	GO:0009987 and GO:0008152 and
	GO:0035023	regulation of Rho protein signal transduction	GO:0009987 and GO:0065007 and GO:0050789
0009E05	GO:0006468	protein amino acid phosphorylation	GO:0009987 and GO:0008152 and
0009G02	GO:0008654	phospholipid biosynthesis	GO:0009987 and GO:0008152
	GO:0009058	biosynthesis	GO:0008152
0013A11	GO:0008152	metabolism	GO:0008152
0013A12	GO:0002119	larval development (sensu Nematoda)	GO:0032502 and GO:0032501
	GO:0009792	embryonic development (sensu Metazoa)	GO:0032501 and GO:0032502 and GO:0000003 and GO:0022414
	GO:0040010	positive regulation of growth rate	GO:0065007 and GO:0040007 and GO:0048518 and

			GO:50789
	GO:0040018	positive regulation of body size	GO:0065007 and GO:0040007 and GO:0032501 and GO:0048518 and GO:0050789
	GO:0040032	post-embryonic body morphogenesis	GO:0032501 and GO:0032502
0013C10	GO:0006817	phosphate transport	GO:0051234 and GO:0051179
0013C11	GO:0006508	proteolysis and peptidolysis	GO:0009987 and GO:0008152 and
0013D04	GO:0006457	protein folding	GO:0009987 and GO:0008152 and
	GO:0006950	response to stress	GO:0050896
	GO:0007049	cell cycle	GO:0009987
0013D05	GO:0007186	G-protein coupled receptor protein signaling pathway	GO:0009987 and GO:0065007 and GO:0050789
0013E03	GO:0006915	apoptosis	GO:0009987 and GO:0032502
0013F06	GO:0007265	Ras protein signal transduction	GO:0009987 and GO:0065007 and GO:0050789
0013F08	GO:0000003	reproduction	GO:0000003
	GO:0002009	morphogenesis of an epithelium	GO:0032502
	GO:0006281	DNA repair	GO:0009987 and GO:0008152 and GO:0050896
	GO:0008406	gonad development	GO:0032501 and GO:0032502 and GO:0000003 and GO:0022414
	GO:0009792	embryonic development (sensu Metazoa)	GO:0032501 and GO:0032502 and GO:0000003 and GO:0022414
	GO:0040025	vulval development (sensu Nematoda)	GO:0032501 and GO:0032502
	GO:0040035	hermaphrodite genital morphogenesis	GO:0032501 and GO:0032502 and GO:0000003 and GO:0022414
0014A02	GO:0002119	larval development (sensu Nematoda)	GO:0032502 and GO:0032501
	GO:0006350	transcription	GO:0009987 and GO:0008152 and GO:0050896

	GO:0009792	embryonic development (sensu Metazoa)	GO:0032501 and GO:0032502 and GO:0000003 and GO:0022414
0014A02	GO:0040007	growth	GO:0040007
	GO:0040035	hermaphrodite genital morphogenesis	GO:0032501 and GO:0032502 and GO:0000003 and GO:0022414
0014A08	GO:0006355	regulation of transcription, DNA-dependent	GO:0065007 and GO:0009987 and GO:0008152 and GO:0050789
0014B10	GO:0006817	phosphate transport	GO:0051234 and GO:0051179
	GO:0040002	cuticle biosynthesis (sensu Nematoda)	GO:0032502 and GO:0032501
0014C05	GO:0002119	larval development (sensu Nematoda)	GO:0032502 and GO:0032501
	GO:0006414	translational elongation	GO:0009987 and GO:0008152 and
	GO:0007276	gametogenesis	GO:0000003
	GO:0009792	embryonic development (sensu Metazoa)	GO:0032501 and GO:0032502 and GO:0000003 and GO:0022414
	GO:0040007	growth	GO:0040007
0014E04	GO:0006397	mRNA processing	GO:0009987 and GO:0008152 and
	GO:0008380	RNA splicing	GO:0009987 and GO:0008152
	GO:0051028	mRNA transport	GO:0051234 and GO:0051179
0014E09	GO:0000003	reproduction	GO:0000003
	GO:0002119	larval development (sensu Nematoda)	GO:0032502 and GO:0032501
	GO:0040007	growth	GO:0040007
0014F02	GO:0006508	proteolysis and peptidolysis	GO:0009987 and GO:0008152 and
0014H02	GO:0006355	regulation of transcription, DNA-dependent	GO:0065007 and GO:0009987 and GO:0008152 and GO:0050789
0014H04	GO:0006516	glycoprotein catabolism	GO:0009987 and GO:0008152 and

0014H09	GO:0006413	translational initiation	GO:0009987 and GO:0008152 and GO:0043933
	GO:0006417	regulation of protein biosynthesis	GO:0009987 and GO:0065007 and GO:0008152 and GO:0050789
001A06	GO:0006633	fatty acid biosynthesis	GO:0009987 and GO:0008152 and
001A08	GO:0006950	response to stress	GO:0050896
	GO:0006952	defense response	GO:0050896
	GO:0008340	determination of adult life span	GO:0032501 and GO:0032502
001B06	GO:0006810	transport	GO:0051234 and GO:0051179
001B08	GO:0006810	transport	GO:0051234 and GO:0051179
001C02	GO:0008152	metabolism	GO:0008152
001D08	GO:0043066	negative regulation of apoptosis	GO:0065007 and GO:0009987 and GO:0032502 and GO:0048519 and GO:0050789
001D12	GO:0006811	ion transport	GO:0051234 and GO:0051179
001F09	GO:0045454	cell redox homeostasis	GO:0065007 and GO:0009987 and GO:0050789
002B12	GO:0006730	one-carbon compound metabolism	GO:0009987 and GO:0008152 and
002C05	GO:0015992	proton transport	GO:0051234 and GO:0051179
002C06	GO:0006096	glycolysis	GO:0009987 and GO:0008152 and
002C10	GO:0006897	endocytosis	GO:0051234 and GO:0051179 and GO:0009987
002D06	GO:0006825	copper ion transport	GO:0051234 and GO:0051179
	GO:0006878	copper ion homeostasis	GO:0065007 and GO:0009987
002H08	GO:0007018	microtubule-based movement	GO:0051234 and GO:0051179 and GO:0009987
	GO:0051258	protein polymerization	GO:0009987 and GO:0043933
	GO:0000003	reproduction	GO:0000003
	GO:0002119	larval development (sensu Nematoda)	GO:0032502 and GO:0032501
	GO:0006412	protein biosynthesis	GO:0009987 and GO:0008152 and

	GO:0009792	embryonic development (sensu Metazoa)	GO:0032501 and GO:0032502 and GO:0000003 and GO:0022414
	GO:0040007	growth	GO:0040007
	GO:0040035	hermaphrodite genital morphogenesis	GO:0032501 and GO:0032502 and GO:0000003 and GO:0022414
003A10	GO:0000003	reproduction	GO:0000003
	GO:0000281	cytokinesis after mitosis	GO:0009987
	GO:0002119	larval development (sensu Nematoda)	GO:0032502 and GO:0032501
	GO:0007517	muscle development	GO:0032501 and GO:0032502
	GO:0030036	actin cytoskeleton organization and biogenesis	GO:0009987
	GO:0040007	growth	GO:0040007
	GO:0040016	embryonic cleavage	GO:0009987 and GO:0032501 and GO:0032502
	GO:0040035	hermaphrodite genital morphogenesis	GO:0032501 and GO:0032502 and GO:0000003 and GO:0022414
003B01	GO:0006468	protein amino acid phosphorylation	GO:0009987 and GO:0008152 and
003B03	GO:0006334	nucleosome assembly	GO:0009987 and GO:0043933
003C04	GO:0008033	tRNA processing	GO:0009987 and GO:0008152
003C05	GO:0006281	DNA repair	GO:0009987 and GO:0008152 and GO:0050896
	GO:0006412	protein biosynthesis	GO:0009987 and GO:0008152 and
	GO:0016567	protein ubiquitination	GO:0009987 and GO:0008152
	GO:0042254	ribosome biogenesis and assembly	GO:0009987
003H11	GO:0006817	phosphate transport	GO:0051234 and GO:0051179
	GO:0009792	embryonic development (sensu Metazoa)	GO:0032501 and GO:0032502 and GO:0000003 and GO:0022414

	GO:0040002	cuticle biosynthesis (sensu Nematoda)	GO:0032502 and GO:0032501
	GO:0040018	positive regulation of body size	GO:0065007 and GO:0040007 and GO:0032501 and GO:0048518 and GO:0050789
004A01	GO:0000003	reproduction	GO:0000003
	GO:0002119	larval development (sensu Nematoda)	GO:0032502 and GO:0032501
	GO:0006412	protein biosynthesis	GO:0009987 and GO:0008152 and
	GO:0009792	embryonic development (sensu Metazoa)	GO:0032501 and GO:0032502 and GO:0000003 and GO:0022414
	GO:0040010	positive regulation of growth rate	GO:0065007 and GO:0040007 and GO:0048518 and GO:50789
004A08	GO:0000132	establishment of mitotic spindle orientation	GO:0009987 and GO:0051179 and
	GO:0000910	cytokinesis	GO:0009987
	GO:0002119	larval development (sensu Nematoda)	GO:0032502 and GO:0032501
	GO:0007018	microtubule-based movement	GO:0051234 and GO:0051179 and GO:0009987
	GO:0007051	spindle organization and biogenesis	GO:0009987
	GO:0008104	protein localization	GO:0051179
	GO:0035046	pronuclear migration	GO:0009987 and GO:0051234 and GO:0051179 and GO:0000003 and GO:0022414
004A08	GO:0040007	growth	GO:0040007
	GO:0040016	embryonic cleavage	GO:0009987 and GO:0032501 and GO:0032502
	GO:0051258	protein polymerization	GO:0009987 and GO:0043933
004B03	GO:0006270	DNA replication initiation	GO:0009987 and GO:0008152 and
	GO:0006470	protein amino acid dephosphorylation	GO:0009987 and GO:0008152 and
	GO:0007050	cell cycle arrest	GO:0009987

004E05	GO:0019478	D-amino acid catabolism	GO:0009987 and GO:0008152
004E10	GO:0007264	small GTPase mediated signal transduction	GO:0009987 and GO:0065007 and GO:0050789
004E12	GO:0006559	L-phenylalanine catabolism	GO:0009987 and GO:0008152 and
	GO:0006572	tyrosine catabolism	GO:0009987 and GO:0008152 and
004G04	GO:0006355	regulation of transcription, DNA-dependent	GO:0065007 and GO:0009987 and GO:0008152 and GO:0050789
004H04	GO:0006810	transport	GO:0051234 and GO:0051179
15A01	GO:0006355	regulation of transcription, DNA-dependent	GO:0065007 and GO:0009987 and GO:0008152 and GO:0050789
	GO:0007254	JNK cascade	GO:0009987 and GO:0065007 and GO:0050789
	GO:0007297	follicle cell migration (sensu Insecta)	GO:0000003 and GO:0040011 and GO:0051179 and GO:0032502 and GO:0009987
	GO:0007391	dorsal closure	GO:0032501 and GO:0032502
	GO:0007464	R3 and R4 cell fate commitment	GO:0032501 and GO:0032502 and GO:0009987
	GO:0019730	antimicrobial humoral response	GO:0002376 and GO:0051704 and GO:0050896
	GO:0042060	wound healing	GO:0050896
	GO:0046529	imaginal disc fusion, thorax closure	GO:0032501 and GO:0032502
15D01	GO:0006461	protein complex assembly	GO:0009987 and GO:0043933
	GO:0006886	intracellular protein transport	GO:0051234 and GO:0051179 and GO:0009987
	GO:0016192	vesicle-mediated transport	GO:0051234 and GO:0051179 and GO:0009987
15E07	GO:0006461	protein complex assembly	GO:0009987 and GO:0043933
	GO:0006886	intracellular protein transport	GO:0051234 and GO:0051179 and GO:0009987
	GO:0016192	vesicle-mediated transport	GO:0051234 and GO:0051179 and GO:0009987
15E08	GO:0006559	L-phenylalanine catabolism	GO:0009987 and GO:0008152 and

	GO:0006572	tyrosine catabolism	GO:0009987 and GO:0008152 and
15E10	GO:0006412	protein biosynthesis	GO:0009987 and GO:0008152 and
	GO:0007276	gametogenesis	GO:0000003
	GO:0009792	embryonic development (sensu Metazoa)	GO:0032501 and GO:0032502 and GO:0000003 and GO:0022414
15G10	GO:0006014	D-ribose metabolism	GO:0009987 and GO:0008152 and
15G12	GO:0006508	proteolysis and peptidolysis	GO:0009987 and GO:0008152 and
	GO:0008219	cell death	GO:0009987 and GO:0032502
16A06	GO:0000910	cytokinesis	GO:0009987
	GO:0006512	ubiquitin cycle	/
	GO:0007138	meiotic anaphase II	GO:0009987
	GO:0008054	cyclin catabolism	GO:0009987 and GO:0008152
	GO:0008595	determination of anterior and posterior axis, embryo	GO:0032501 and GO:0032502
	GO:0040016	embryonic cleavage	GO:0009987 and GO:0032501 and GO:0032502
	GO:0040035	hermaphrodite genital morphogenesis	GO:0032501 and GO:0032502 and GO:0000003 and GO:0022414
16B10	GO:0006508	proteolysis and peptidolysis	GO:0009987 and GO:0008152 and
16C07	GO:0000003	reproduction	GO:0000003
	GO:0002119	larval development (sensu Nematoda)	GO:0032502 and GO:0032501
	GO:0006461	protein complex assembly	GO:0009987 and GO:0043933
	GO:0006886	intracellular protein transport	GO:0051234 and GO:0051179 and GO:0009987
	GO:0016192	vesicle-mediated transport	GO:0051234 and GO:0051179 and GO:0009987
16D06	GO:0009792	embryonic development (sensu Metazoa)	GO:0032501 and GO:0032502 and GO:0000003 and GO:0022414

16E03	GO:0006886	intracellular protein transport	GO:0051234 and GO:0051179 and GO:0009987
	GO:0016192	vesicle-mediated transport	GO:0051234 and GO:0051179 and GO:0009987
16G08	GO:0002164	larval development	GO:0032502 and GO:0032501
	GO:0007444	imaginal disc development	GO:0032501 and GO:0032502
	GO:0030154	cell differentiation	GO:0009987 and GO:0032502
	GO:0048477	oogenesis	GO:0000003
Total (171)	Total (408)	Total (172), not including repeat descriptions	

Additional file 4-6. GO classification of 378 gene clusters based on cellular component

Clone NO.	Subcategory ID.	Definition of Subcategory term	Main category
00010A08	GO:0005851	eukaryotic translation initiation factor 2B complex	GO:0005623 and GO:0044464 and GO:0032991
00010A12	GO:0005843	cytosolic small ribosomal subunit (sensu Eukaryota)	GO:0005623 and GO:0044464 and GO:0044424 and GO:0032991 and GO:0043226 and GO:0044422
00010B03	GO:0000778	condensed nuclear chromosome kinetochore	GO:0005623 and GO:0044464 and GO:0032991 and GO:0043226
	GO:0005816	spindle pole body	GO:0005623 and GO:0044464 and GO:0043226 and GO:0044422
00010B05	GO:0016020	membrane	GO:0005623 and GO:0044464
00010B06	GO:0005737	cytoplasm	GO:0005623 and GO:0044464
	GO:0008290	F-actin capping protein complex	GO:0005623 and GO:0044464 and GO:0032991 and GO:0043226 and GO:0044422
00010B08	GO:0005739	mitochondrion	GO:0005623 and GO:0044464 and GO:0043226
	GO:0016021	integral to membrane	GO:0005623 and GO:0044464
00010B11	GO:0005743	mitochondrial inner membrane	GO:0005623 and GO:0044464 and GO:0031975 and GO:0043226 and GO:0044422
	GO:0016021	integral to membrane	GO:0005623 and GO:0044464
00010B12	GO:0005746	mitochondrial electron transport chain	GO:0005623 and GO:0044464
	GO:0016021	integral to membrane	GO:0005623 and GO:0044464
00010C01	GO:0005788	endoplasmic reticulum lumen	GO:0005623 and GO:0044464 and GO:0031974 and GO:0043226 and GO:0044422
00010C11	GO:0005746	mitochondrial electron transport chain	GO:0005623 and GO:0044464
	GO:0016021	integral to membrane	GO:0005623 and GO:0044464

00010D02	GO:0005739	mitochondrion	GO:0005623 and GO:0044464 and GO:0043226
	GO:0016021	integral to membrane	GO:0005623 and GO:0044464
00010D06	GO:0005634	nucleus	GO:0005623 and GO:0044464 and GO:0043226
	GO:0005739	mitochondrion	GO:0005623 and GO:0044464 and GO:0043226
00010D11	GO:0005737	cytoplasm	GO:0005623 and GO:0044464
	GO:0005874	microtubule	GO:0005623 and GO:0044464 and GO:0043226 and GO:0044422
	GO:0043025	cell soma	GO:0005623 and GO:0044464
	GO:0043198	dendritic shaft	GO:0005623 and GO:0044464
00010D12	GO:0005576	extracellular region	GO:0005576
	GO:0005737	cytoplasm	GO:0005623 and GO:0044464
00010E06	GO:0000785	chromatin	GO:0005623 and GO:0044464 and GO:0043226 and GO:0044422
	GO:0042555	MCM complex	GO:0005623 and GO:0044464 and GO:0032991 and GO:0031974 and GO:0043226
00010E08	GO:0016459	myosin	GO:0005623 and GO:0044464 and GO:0032991 and GO:0043226 and GO:0044422
00010E09	GO:0005778	peroxisomal membrane	GO:0005623 and GO:0044464 and GO:0043226 and GO:0044422
	GO:0016021	integral to membrane	GO:0005623 and GO:0044464
00010E12	GO:0005764	lysosome	GO:0005623 and GO:0044464 and GO:0043226 and
	GO:0005886	plasma membrane	GO:0005623 and GO:0044464
	GO:0016021	integral to membrane	GO:0005623 and GO:0044464
00010F01	GO:0005737	cytoplasm	GO:0005623 and GO:0044464
00010F04	GO:0005739	mitochondrion	GO:0005623 and GO:0044464 and GO:0043226
00010F05	GO:0005634	nucleus	GO:0005623 and GO:0044464 and GO:0043226
00010F07	GO:0005576	extracellular region	GO:0005576
	GO:0005764	lysosome	GO:0005623 and GO:0044464 and GO:0043226 and

00010G05	GO:0005746	mitochondrial electron transport chain	GO:0005623 and GO:0044464
	GO:0016021	integral to membrane	GO:0005623 and GO:0044464
00010G09	GO:0005737	cytoplasm	GO:0005623 and GO:0044464
00010G12	GO:0005576	extracellular region	GO:0005576
	GO:0005737	cytoplasm	GO:0005623 and GO:0044464
00010H06	GO:0005737	cytoplasm	GO:0005623 and GO:0044464
00010H10	GO:0005783	endoplasmic reticulum	GO:0005623 and GO:0044464 and GO:0043226
	GO:0005794	Golgi apparatus	GO:0005623 and GO:0044464 and GO:0043226
00010H12	GO:0005634	nucleus	GO:0005623 and GO:0044464 and GO:0043226
	GO:0005794	Golgi apparatus	GO:0005623 and GO:0044464 and GO:0043226
	GO:0005905	coated pit	GO:0005623 and GO:0044464
	GO:0012506	vesicle membrane	GO:0005623 and GO:0044464 and GO:0043226 and GO:0044422
	GO:0016459	myosin	GO:0005623 and GO:0044464 and GO:0032991 and GO:0043226 and GO:0044422
	GO:0030424	axon	GO:0005623 and GO:0044464
	GO:0043025	cell soma	GO:0005623 and GO:0044464
00011A08	GO:0031314	extrinsic to mitochondrial inner membrane	GO:0005623 and GO:0044464
00011A09	GO:0005737	cytoplasm	GO:0005623 and GO:0044464
00011B11	GO:0005737	cytoplasm	GO:0005623 and GO:0044464
00011C06	GO:0005737	cytoplasm	GO:0005623 and GO:0044464
	GO:0005874	microtubule	GO:0005623 and GO:0044464 and GO:0043226 and GO:0044422
00011D09	GO:0005853	eukaryotic translation elongation factor 1 complex	GO:0005623 and GO:0044464 and GO:0044424 and GO:0032991 and GO:0043226 and GO:0044422
00011D10	GO:0005783	endoplasmic reticulum	GO:0005623 and GO:0044464 and GO:0043226

	GO:0005794	Golgi apparatus	GO:0005623 and GO:0044464 and GO:0043226
	GO:0030126	COPI vesicle coat	GO:0005623
00011E02	GO:0005737	cytoplasm	GO:0005623 and GO:0044464
00011F11	GO:0005737	cytoplasm	GO:0005623 and GO:0044464
	GO:0008290	F-actin capping protein complex	GO:0005623 and GO:0044464 and GO:0032991 and GO:0043226 and GO:0044422
00011G04	GO:0005739	mitochondrion	GO:0005623 and GO:0044464 and GO:0043226
00011H03	GO:0005575	cellular_component	GO:0005575
00011H07	GO:0005739	mitochondrion	GO:0005623 and GO:0044464 and GO:0043226
00011H08	GO:0005672	transcription factor TFIIA complex	GO:0005623 and GO:0044464 and GO:0032991 and GO:0031974 and GO:0043226 and GO:0044422
00011H10	GO:0005853	eukaryotic translation elongation factor 1 complex	GO:0005623 and GO:0044464 and GO:0044424 and GO:0032991 and GO:0043226 and GO:0044422
00012A04	GO:0005737	cytoplasm	GO:0005623 and GO:0044464
	GO:0016020	membrane	GO:0005623 and GO:0044464
00012A05	GO:0005737	cytoplasm	GO:0005623 and GO:0044464
00012B06	GO:0016021	integral to membrane	GO:0005623 and GO:0044464
	GO:0005634	nucleus	GO:0005623 and GO:0044464 and GO:0043226
00012B07	GO:0005737	cytoplasm	GO:0005623 and GO:0044464
00012C03	GO:0005783	endoplasmic reticulum	GO:0005623 and GO:0044464 and GO:0043226
00012C04	GO:0005615	extracellular space	GO:0005576 and GO:0044421
	GO:0005634	nucleus	GO:0005623 and GO:0044464 and GO:0043226
00012C06	GO:0048471	perinuclear region	GO:0005623 and GO:0044464
00012D12	GO:0000786	nucleosome	GO:0005623 and GO:0044464 and GO:0032991 and GO:0043226 and GO:0044422

	GO:0005634	nucleus	GO:0005623 and GO:0044464 and GO:0043226
00012E06	GO:0005739	mitochondrion	GO:0005623 and GO:0044464 and GO:0043226
00012E12	GO:0016021	integral to membrane	GO:0005623 and GO:0044464
00012F04	GO:0005634	nucleus	GO:0005623 and GO:0044464 and GO:0043226
00012F12	GO:0016021	integral to membrane	GO:0005623 and GO:0044464
00012G02	GO:0005578	extracellular matrix (sensu Metazoa)	GO:0005576 and GO:0044421
00012G03	GO:0016021	integral to membrane	GO:0005623 and GO:0044464
00012G04	GO:0000794	condensed nuclear chromosome	GO:0005623 and GO:0044464 and GO:0043226 and GO:0044422
	GO:0030018	Z disc	GO:0005623 and GO:0044464
00012G05	GO:0005840	ribosome	GO:0005623 and GO:0044464 and GO:0032991 and GO:0043226
00012G06	GO:0005634	nucleus	GO:0005623 and GO:0044464 and GO:0043226
00012G09	GO:0005739	mitochondrion	GO:0005623 and GO:0044464 and GO:0043226
	GO:0016021	integral to membrane	GO:0005623 and GO:0044464
00012H04	GO:0005783	endoplasmic reticulum	GO:0005623 and GO:0044464 and GO:0043226
00012H05	GO:0005622	intracellular	GO:0005623 and GO:0044464
0005A11	GO:0016021	integral to membrane	GO:0005623 and GO:0044464
0005B06	GO:0005634	nucleus	GO:0005623 and GO:0044464 and GO:0043226
	GO:0005829	cytosol	GO:0005623 and GO:0044464
	GO:0043234	protein complex	GO:0032991
0005C03	GO:0005737	cytoplasm	GO:0005623 and GO:0044464
0005E02	GO:0005622	intracellular	GO:0005623 and GO:0044464
0005F01	GO:0016021	integral to membrane	GO:0005623 and GO:0044464
0005F12	GO:0005764	lysosome	GO:0005623 and GO:0044464 and GO:0043226 and
0005G12	GO:0005739	mitochondrion	GO:0005623 and GO:0044464 and GO:0043226
	GO:0016021	integral to membrane	GO:0005623 and GO:0044464

0005H10	GO:0005739	mitochondrion	GO:0005623 and GO:0044464 and GO:0043226
	GO:0005840	ribosome	GO:0005623 and GO:0044464 and GO:0032991 and GO:0043226
0005H11	GO:0005634	nucleus	GO:0005623 and GO:0044464 and GO:0043226
	GO:0005737	cytoplasm	GO:0005623 and GO:0044464
0006A10	GO:0005576	extracellular region	GO:0005576
	GO:0005737	cytoplasm	GO:0005623 and GO:0044464
0006D06	GO:0016020	membrane	GO:0005623 and GO:0044464
0006G05	GO:0016021	integral to membrane	GO:0005623 and GO:0044464
0006H06	GO:0005622	intracellular	GO:0005623 and GO:0044464
0006H08	GO:0005730	nucleolus	GO:0005623 and GO:0044464 and GO:0031974 and GO:0043226 and GO:0044422
	GO:0005829	cytosol	GO:0005623 and GO:0044464
	GO:0005840	ribosome	GO:0005623 and GO:0044464 and GO:0032991 and GO:0043226
0007B01	GO:0005681	spliceosome complex	GO:0005623 and GO:0044464 and GO:0032991 and GO:0043226 and GO:0044422
0007B02	GO:0005622	intracellular	GO:0005623 and GO:0044464
0007B06	GO:0005737	cytoplasm	GO:0005623 and GO:0044464
0007B08	GO:0005737	cytoplasm	GO:0005623 and GO:0044464
0007B10	GO:0005840	ribosome	GO:0005623 and GO:0044464 and GO:0032991 and GO:0043226
0007B12	GO:0005576	extracellular region	GO:0005576
0007C01	GO:0005634	nucleus	GO:0005623 and GO:0044464 and GO:0043226
	GO:0005737	cytoplasm	GO:0005623 and GO:0044464
0007C05	GO:0005739	mitochondrion	GO:0005623 and GO:0044464 and GO:0043226
0007C06	GO:0016469	proton-transporting two-sector ATPase complex	GO:0005623 and GO:0044464 and GO:0032991

0007C08	GO:0005634	nucleus	GO:0005623 and GO:0044464 and GO:0043226
0007G01	GO:0005576	extracellular region	GO:0005576
0007G03	GO:0005576	extracellular region	GO:0005576
	GO:0005737	cytoplasm	GO:0005623 and GO:0044464
0007G04	GO:0005840	ribosome	GO:0005623 and GO:0044464 and GO:0032991 and GO:0043226
0007H10	GO:0016021	integral to membrane	GO:0005623 and GO:0044464
0008A07	GO:0005634	nucleus	GO:0005623 and GO:0044464 and GO:0043226
	GO:0030529	ribonucleoprotein complex	GO:0005623 and GO:0044464 and GO:0032991
0008B12	GO:0005578	extracellular matrix (sensu Metazoa)	GO:0005576 and GO:0044421
0008C01	GO:0016021	integral to membrane	GO:0005623 and GO:0044464
0008C06	GO:0005739	mitochondrion	GO:0005623 and GO:0044464 and GO:0043226
0008C10	GO:0016021	integral to membrane	GO:0005623 and GO:0044464
0008D02	GO:0005634	nucleus	GO:0005623 and GO:0044464 and GO:0043226
0008F03	GO:0005737	cytoplasm	GO:0005623 and GO:0044464
0009A05	GO:0000139	Golgi membrane	GO:0005623 and GO:0044464 and GO:0043226 and GO:0044422
	GO:0005783	endoplasmic reticulum	GO:0005623 and GO:0044464 and GO:0043226
	GO:0005887	integral to plasma membrane	GO:0005623 and GO:0044464
0009A08	GO:0005634	nucleus	GO:0005623 and GO:0044464 and GO:0043226
0009B11	GO:0005634	nucleus	GO:0005623 and GO:0044464 and GO:0043226
	GO:0005737	cytoplasm	GO:0005623 and GO:0044464
0009C09	GO:0005575	cellular_component	GO:0005575
0009C10	GO:0016021	integral to membrane	GO:0005623 and GO:0044464
0009D02	GO:0005635	nuclear membrane	GO:0005623 and GO:0044422 and GO:0043226 and GO:0031975 and GO:0044464
	GO:0005819	spindle	GO:0005623 and GO:0044464 and GO:0043226 and GO:0044422

	GO:0005874	microtubule	GO:0005623 and GO:0044464 and GO:0043226 and GO:0044422
	GO:0005938	cell cortex	GO:0005623 and GO:0044464
	GO:0030286	dynein complex	GO:0005623 and GO:0044464 and GO:0032991 and GO:0043226 and GO:0044422
0009E02	GO:0005622	intracellular	GO:0005623 and GO:0044464
0009E05	GO:0005737	cytoplasm	GO:0005623 and GO:0044464
0013A11	GO:0016021	integral to membrane	GO:0005623 and GO:0044464
0013C10	GO:0005737	cytoplasm	GO:0005623 and GO:0044464
0013C11	GO:0005622	intracellular	GO:0005623 and GO:0044464
0013D04	GO:0005737	cytoplasm	GO:0005623 and GO:0044464
0013D05	GO:0016021	integral to membrane	GO:0005623 and GO:0044464
0013F08	GO:0005634	nucleus	GO:0005623 and GO:0044464 and GO:0043226
0014A02	GO:0005634	nucleus	GO:0005623 and GO:0044464 and GO:0043226
0014A08	GO:0005634	nucleus	GO:0005623 and GO:0044464 and GO:0043226
0014B10	GO:0005576	extracellular region	GO:0005576
	GO:0005737	cytoplasm	GO:0005623 and GO:0044464
0014C05	GO:0005737	cytoplasm	GO:0005623 and GO:0044464
0014C11	GO:0000299	integral to membrane of membrane fraction	GO:0005623 and GO:0044464
	GO:0005625	soluble fraction	GO:0005623 and GO:0044464
	GO:0005794	Golgi apparatus	GO:0005623 and GO:0044464 and GO:0043226
	GO:0016020	membrane	GO:0005623 and GO:0044464
0014E04	GO:0005634	nucleus	GO:0005623 and GO:0044464 and GO:0043226
0014E09	GO:0030529	ribonucleoprotein complex	GO:0005623 and GO:0044464 and GO:0032991
0014G11	GO:0005739	mitochondrion	GO:0005623 and GO:0044464 and GO:0043226

0014H02	GO:0005634	nucleus	GO:0005623 and GO:0044464 and GO:0043226
0014H04	GO:0005764	lysosome	GO:0005623 and GO:0044464 and GO:0043226 and
0014H05	GO:0005737	cytoplasm	GO:0005623 and GO:0044464
	GO:0005856	cytoskeleton	GO:0005623 and GO:0044464 and GO:0043226
	GO:0019717	synaptosome	GO:0005623 and GO:0044464
	GO:0030054	cell junction	GO:0005623 and GO:0044464
	GO:0030426	growth cone	GO:0005623 and GO:0044464
	GO:0042734	presynaptic membrane	GO:0005623 and GO:0044464 and GO:0045202 and GO:0044456
0014H06	GO:0005634	nucleus	GO:0005623 and GO:0044464 and GO:0043226
	GO:0005737	cytoplasm	GO:0005623 and GO:0044464
0014H09	GO:0005737	cytoplasm	GO:0005623 and GO:0044464
001A06	GO:0005783	endoplasmic reticulum	GO:0005623 and GO:0044464 and GO:0043226
	GO:0016021	integral to membrane	GO:0005623 and GO:0044464
001B06	GO:0005887	integral to plasma membrane	GO:0005623 and GO:0044464
	GO:0008021	synaptic vesicle	GO:0005623 and GO:0044464 and GO:0043226
	GO:0030054	cell junction	GO:0005623 and GO:0044464
	GO:0045202	synapse	GO:0045202
001B08	GO:0016021	integral to membrane	GO:0005623 and GO:0044464
001C02	GO:0016021	integral to membrane	GO:0005623 and GO:0044464
001D08	GO:0016021	integral to membrane	GO:0005623 and GO:0044464
001D12	GO:0016021	integral to membrane	GO:0005623 and GO:0044464
001F09	GO:0005783	endoplasmic reticulum	GO:0005623 and GO:0044464 and GO:0043226
002C05	GO:0005739	mitochondrion	GO:0005623 and GO:0044464 and GO:0043226
	GO:0016469	proton-transporting two-sector ATPase complex	GO:0005623 and GO:0044464 and GO:0032991

002C05	GO:0045263	proton-transporting ATP synthase complex, coupling factor F(o)	GO:0005623 and GO:0044464 and GO:0032991
002C10	GO:0016021	integral to membrane	GO:0005623 and GO:0044464
002F08	GO:0005737	cytoplasm	GO:0005623 and GO:0044464
	GO:0005856	cytoskeleton	GO:0005623 and GO:0044464 and GO:0043226
002F11	GO:0005622	intracellular	GO:0005623 and GO:0044464
002G08	GO:0005737	cytoplasm	GO:0005623 and GO:0044464
	GO:0005874	microtubule	GO:0005623 and GO:0044464 and GO:0043226 and GO:0044422
	GO:0043234	protein complex	GO:0032991
002H08	GO:0005737	cytoplasm	GO:0005623 and GO:0044464
003A10	GO:0005737	cytoplasm	GO:0005623 and GO:0044464
	GO:0005856	cytoskeleton	GO:0005623 and GO:0044464 and GO:0043226
003B03	GO:0005634	nucleus	GO:0005623 and GO:0044464 and GO:0043226
003C05	GO:0005634	nucleus	GO:0005623 and GO:0044464 and GO:0043226
	GO:0005739	mitochondrion	GO:0005623 and GO:0044464 and GO:0043226
	GO:0005842	cytosolic large ribosomal subunit (sensu Eukaryota)	GO:0005623 and GO:0044464 and GO:0032991 and GO:0043226 and GO:0044422
003E06	GO:0005829	cytosol	GO:0005623 and GO:0044464
	GO:0043234	protein complex	GO:0032991
003G10	GO:0016021	integral to membrane	GO:0005623 and GO:0044464
003H11	GO:0005737	cytoplasm	GO:0005623 and GO:0044464
004A01	GO:0015934	large ribosomal subunit	GO:0005623 and GO:0044464 and GO:0032991 and GO:0043226 and GO:0044422
004A08	GO:0005874	microtubule	GO:0005623 and GO:0044464 and GO:0043226 and GO:0044422
	GO:0043234	protein complex	GO:0032991

004B03	GO:0000159	protein phosphatase type 2A complex	GO:0005623 and GO:0044464 and GO:0032991
	GO:0005634	nucleus	GO:0005623 and GO:0044464 and GO:0043226
004E05	GO:0005737	cytoplasm	GO:0005623 and GO:0044464
004E10	GO:0005622	intracellular	GO:0005623 and GO:0044464
004G04	GO:0005634	nucleus	GO:0005623 and GO:0044464 and GO:0043226
004G07	GO:0005737	cytoplasm	GO:0005623 and GO:0044464
	GO:0005856	cytoskeleton	GO:0005623 and GO:0044464 and GO:0043226
004H04	GO:0008021	synaptic vesicle	GO:0005623 and GO:0044464 and GO:0043226
	GO:0016021	integral to membrane	GO:0005623 and GO:0044464
	GO:0030054	cell junction	GO:0005623 and GO:0044464
	GO:0045202	synapse	GO:0045202
15A01	GO:0005634	nucleus	GO:0005623 and GO:0044464 and GO:0043226
	GO:0005737	cytoplasm	GO:0005623 and GO:0044464
15D01	GO:0005794	Golgi apparatus	GO:0005623 and GO:0044464 and GO:0043226
	GO:0030131	clathrin adaptor complex	GO:0005623 and GO:0044464 and GO:0032991
15D11	GO:0005737	cytoplasm	GO:0005623 and GO:0044464
	GO:0005856	cytoskeleton	GO:0005623 and GO:0044464 and GO:0043226
15E05	GO:0016021	integral to membrane	GO:0005623 and GO:0044464
15E07	GO:0005783	endoplasmic reticulum	GO:0005623 and GO:0044464 and GO:0043226
	GO:0005794	Golgi apparatus	GO:0005623 and GO:0044464 and GO:0043226
	GO:0030126	COPI vesicle coat	GO:0005623
15E10	GO:0005840	ribosome	GO:0005623 and GO:0044464 and GO:0032991 and GO:0043226
15F05	GO:0005739	mitochondrion	GO:0005623 and GO:0044464 and GO:0043226
16B10	GO:0005764	lysosome	GO:0005623 and GO:0044464 and GO:0043226 and
16C07	GO:0005783	endoplasmic reticulum	GO:0005623 and GO:0044464 and GO:0043226

	GO:0005794	Golgi apparatus	GO:0005623 and GO:0044464 and GO:0043226
	GO:0030131	clathrin adaptor complex	GO:0005623 and GO:0044464 and GO:0032991
16E03	GO:0005783	endoplasmic reticulum	GO:0005623 and GO:0044464 and GO:0043226
	GO:0005794	Golgi apparatus	GO:0005623 and GO:0044464 and GO:0043226
16E03	GO:0016020	membrane	GO:0005623 and GO:0044464
16E06	GO:0005615	extracellular space	GO:0005576 and GO:0044421
16G08	GO:0005575	cellular_component	GO:0005575
Total (162)	Total (254)	Total (69), not including repeat descriptions	
