# Supplementary material for: Preliminary molecular characterization of the human pathogen Angiostrongylus cantonensis
Source: BMC Mol Biol. 2009 Oct 25;10:97. doi: 10.1186/1471-2199-10-97 (PMC2774698; doi:10.1186/1471-2199-10-97)
Supplement: Additional file 5 — cDNA with catalytic activity was involved in metabolism. The data provided represent the statistical analysis of catalytic activity of putative protein productes of cDNA involved in metabolism. N indicates copies of cDNA in the first batch sequencing. * indicates more than two items base on Gene ontology, and the other terms could be found in table S4. \ indicates there was no item description for cDNA based on Gene ontology. Some cDNAs factually could not be predicted, but with some others owing to not full-length of cDNA sequence. ?, Not sure what metabolism pathway related. [file 1471-2199-10-97-S5.PDF]

Additional file 5. Gene with catalytic activity involved in metabolism

Clone No.	N	Homologous gene	Gene ontology			Metabolism pathway
			Molecular function	Biology process	Cellular component	
00010A09	3	ASpartyl Protease family member (asp-2) [*Caenorhabditis elegans*]	pepsin A activity	proteolysis and peptidolysis; cell death	\	Protein metabolism
00012F09	10	cathePsin Z family member (cpz-1) [*Caenorhabditis elegans*]	cysteine-type endopeptidase activity	proteolysis and peptidolysis	\	Protein metabolism
0005E12	3	cathepsin A [*Monodelphis domestica*]	proteolysis and peptidolysis	proteolysis and peptidolysis	\	Protein metabolism
0005F12	4	cathepsin L 1 [*Dictyocaulus viviparus*]	cathepsin L activity; serine carboxypeptidase activity	proteolysis and peptidolysis; cell differentiation	lysosome	Protein metabolism
0007D03	1	cathepsin B-like cysteine protease 2 [*Parelaphostrongylus tenuis*]	cysteine-type endopeptidase activity	proteolysis and peptidolysis	\	Protein metabolism
0008H03	1	Hypothetical protein T06A4.3a [*Caenorhabditis elegans*]	carboxypeptidase A activity	proteolysis and peptidolysis	\	Protein metabolism
0009D10	1	Prolyl Carboxy Peptidase like family member (pcp-1)[*Caenorhabditis elegans*]	serine-type endopeptidase activity	proteolysis and peptidolysis	\	Protein metabolism
0014F02	2	cathepsin B-like cysteine protease 1 [*Parelaphostrongylus tenuis*]	cysteine-type endopeptidase activity	proteolysis and peptidolysis	\	Protein metabolism
0013C11	1	CaLPain family member (clp-1) [*Caenorhabditis elegans*]	calpain activity	proteolysis and peptidolysis	intracellular	Protein metabolism
15G12	1	ASpartyl Protease family member (asp-1)	pepsin A activity	proteolysis and	\	Protein

		[*Caenorhabditis elegans*]		peptidolysis; cell death		metabolism
16B10	14	cathepsin D-like aspartic protease [*Ancylostoma ceylanicum*]	cathepsin D activity	proteolysis and peptidolysis	lysosome	Protein metabolism
002B12	1	S-adenosylmethionine synthetase C06E7.1 (Methionine adenosyltransferase) (AdoMet synthetase) [*Caenorhabditis elegans*]	methionine adenosyltransferase activity	one-carbon compound metabolism	\	Protein metabolism
0007D04	1	saccharopine dehydrogenase (putative) [*Gallus gallus*]	saccharopine dehydrogenase activity	\	\	Protein metabolism
00010D03	2	pterin-4-alpha-carbinolamine dehydratase [*Diaphorina citri*]	4-alpha-hydroxytetrah ydrobiopterin dehydratase activity	\	\	Protein metabolism
00010B05	1	peptidyl-glycine alpha-amidating monooxygenase precursor (EC 1.14.17.3)[*Rattus norvegicus*]	peptidylglycine monooxygenase activity	peptide metabolism	membrane	Protein metabolism
004E12	1	HomoGentisate Oxidase family member (hgo-1) [*Caenorhabditis elegans*]	homogentisate 1,2-dioxygenase activity	L-phenylalanine catabolism; tyrosine catabolism	\	Protein metabolism
15E08	1	4-HydroxyPhenylpyruvate Dioxygenase family member (hpd-1)[*Caenorhabditis elegans*]	4-hydroxyphenylpyruv ate dioxygenase activity	L-phenylalanine catabolism; tyrosine catabolism	\	Protein metabolism
00012F02	1	Hypothetical protein ZK1128.1 [*Caenorhabditis elegans*]	methyltransferase activity	\	\	Protein metabolism
0014H04	1	Glycosylasparaginase N(4)-(beta-N-acetylglucosaminyl)-L-asparaginase [*Caenorhabditis elegans*]	N4-(beta-N-acetylgluc osaminyl)-L-asparagin ase activity	glycoprotein catabolism	lysosome	Protein metabolism
0007F05	1	Methyltransferase-like protein [*Brugia malayi*]	methyltransferase	\	\	Protein

			activity			metabolism
0005B06	1	26S proteasome regulatory chain 4[*Brugia malayi*]	nucleoside-triphosphatase activity	protein catabolism	nucleus; cytosol; &	Protein metabolism
00012C03	8	protein disulfide isomerase [*Ancylostoma caninum*]	protein disulfide isomerase activity	cell redox homeostasis	endoplasmic reticulum	Protein metabolism
004G07	2	NADH Ubiquinone Oxidoreductase family member (nuo-3) [*Caenorhabditis elegans*]	oxidoreductase activity	\	cytoplasm; cytoskeleton	Energy metabolism
00010B08	14	NADH dehydrogenase subunit 1 [*Ancylostoma duodenale*]	NADH dehydrogenase (ubiquinone) activity	mitochondrial electron transport, NADH to ubiquinone	mitochondrion; integral to membrane	Energy metabolism
00010D02	3	NADH dehydrogenase subunit 2 [*Ancylostoma duodenale*]	NADH dehydrogenase (ubiquinone) activity	\	mitochondrion; integral to membrane	Energy metabolism
00010C11	1	cytochrome b [*Necator americanus*]	oxidoreductase activity	electron transport; transport	mitochondrial electron transport chain; integral to membrane	Energy metabolism
00010G05	31	cytochrome c oxidase subunit I [*Cooperia oncophora*]	cytochrome-c oxidase activity	aerobic respiration; transport; *	mitochondrial electron transport; integral to membrane	Energy metabolism
00012G09	5	cytochrome c oxidase subunit 3 [*Haemonchus contortus*]	cytochrome-c oxidase activity	electron transport	mitochondrion; integral to membrane	Energy metabolism

0005G12	1	NADH dehydrogenase subunit 5 [*Necator americanus*]	NADH dehydrogenase (ubiquinone) activity	ATP synthesis coupled electron transport	mitochondrion; integral to membrane	Energy metabolism
0008C06	1	NADH dehydrogenase subunit 3 [*Heterorhabditis bacteriophora*]	NADH dehydrogenase (ubiquinone) activity	mitochondrial electron transport, NADH to ubiquinone	mitochondrion	Energy metabolism
15F05	1	NADH dehydrogenase subunit 6 [*Ancylostoma duodenale*]	NADH dehydrogenase (ubiquinone) activity	\	mitochondrion	Energy metabolism
00010B12	3	cytochrome c oxidase subunit II [*Haemonchus contortus*]	cytochrome-c oxidase activity	electron transport; transport	mitochondrial electron transport chain; integral to membrane	Energy metabolism
002C05	1	ATPase subunit 6 [*Necator americanus*]	hydrolase activity	proton transport	mitochondrion; proton-transporting ATP synthase complex, coupling factor F(o); &	Energy metabolism
001A06	1	FATty acid desaturase family member (fat-2) [*Caenorhabditis elegans*]	oxidoreductase activity	fatty acid biosynthesis	endoplasmic reticulum; integral to membrane	Lipid metabolism
003B08	1	AMine oXidase family member (amx-3)[*Caenorhabditis elegans*]	oxidoreductase activity	\	\	Lipid metabolism

0006C03	1	gamma-butyrobetaine,2-oxoglutarate dioxygenase; GAMMA-BBH,[*Angiostrongylus cantonensis*]	gamma-butyrobetaine dioxygenase activity	electron transport; carnitine biosynthesis	\	Lipid metabolism
00011H07	1	acyl-coa dehydrogenase [*Aedes aegypti*]	isovaleryl-CoA dehydrogenase activity	electron transport	mitochondrion	Lipid metabolism
0013A11	2	Hypothetical protein CBG00644 [*Caenorhabditis briggsae AF16*]	C-4 methylsterol oxidase activity	metabolism	integral to membrane	Lipid metabolism
0009G02	1	choline-phosphate cytidylyltransferase [*Caenorhabditis elegans*]	choline-phosphate cytidylyltransferase activity	phospholipid biosynthesis; biosynthesis	\	Lipid metabolism
0007G01	1	Acid SphingoMyelinase family member (asm-3) [*Caenorhabditis elegans*]	sphingomyelin phosphodiesterase activity	sphingomyelin catabolism; ceramide biosynthesis	extracellular region	Lipid metabolism
0007B08	1	Phospholipase/Carboxylesterase family protein [*Brugia malayi*]	hydrolase activity	fatty acid metabolism	cytoplasm	Lipid metabolism
00010B02	1	kinase JNK-1 [*Ancylostoma caninum*]	MAP kinase activity	protein amino acid phosphorylation; JNK cascade	\	Signal tranduction
00010B03	1	protein kinase [*Candida albicans SC5314*]	protein serine and threonine kinase activity	protein amino acid phosphorylation; spindle assembly; *	condensed nuclear chromosome kinetochore; spindle pole body	Signal tranduction
003B01	1	cyclic AMP-dependent protein kinase [*Caenorhabditis elegans*]	cAMP-dependent protein kinase activity	protein amino acid phosphorylation	\	Signal tranduction
0005C03	1	Nucleoside diphosphate kinase [*Brugia malayi*]	nucleoside-diphosphat	GTP biosynthesis; UTP	cytoplasm	Signal

			e kinase activity	biosynthesis; *		tranduction and transcription regulation
00010F04	1	Elongation factor Tu GTP binding domain containing protein [*Brugia malayi*]	GTPase activity	regulation of translational initiation	mitochondrion	Transcription regulation
00010G09	5	elongation factor 1 alpha [*Dictyocaulus viviparus*]	GTPase activity	larval development; translational elongation; *	cytoplasm	Transcription regulation
002H08	1	Elongation FacTor family member (eft-2) [*Caenorhabditis elegans*]	GTPase activity	microtubule-based movement; protein polymerization; *	cytoplasm	Transcription regulation
0009D02	1	Dynein Heavy Chain family member (dhc-1)[*Caenorhabditis elegans*]	ATPase activity	cytokinesis; pronuclear migration; *	nuclear membrane; spindle; &	?
002G08	1	beta-tubulin [*Dictyocaulus viviparus*]	GTPase activity	\	cytoplasm; microtubule; &	?
004A08	1	TuBulin, Alpha family member (tba-4) [*Caenorhabditis elegans*]	GTPase activity	cytokinesis; pronuclear migration; *	microtubule; protein complex	?
00010E06	2	yeast MCM (licensing factor) related family member (mcm-7)[*Caenorhabditis elegans*]	DNA-dependent ATPase activity	regulation of DNA replication initiation; cell cycle	chromatin; MCM complex	DNA replication
0013F08	1	SNF2 family N-terminal domain containing protein [*Brugia malayi*]	helicase activity	DNA repair; gonad development; *	nucleus	DNA replication

004B03	1	EF hand family protein [*Brugia malayi*]	protein phosphatase type 2A activity	DNA replication initiation; cell cycle arrest; *	nucleus; protein phosphatase type 2A complex	DNA replication
0008A07	1	H/ACA ribonucleoprotein complex subunit 4 [*Caenorhabditis elegans*]	tRNA-pseudouridine synthase activity	rRNA processing; gametogenesis; *	nucleus; ribonucleoprotein complex	DNA replication
0014A02	1	Rna polymerase ii (b) subunit protein 8 [*Caenorhabditis elegans*]	DNA-directed RNA polymerase activity	transcription; larval development; *	nucleus	DNA replication
00012G03	1	DeHydrogenases, Short chain family member (dhs-5) [*Caenorhabditis elegans*]	oxidoreductase activity	metabolism	integral to membrane	?
00011F08	1	L-lactate dehydrogenase (LDH) [Caenorhabditis elegans]	L-lactic acid dehydrogenase activity	oxidoreductase process; modifed Embden-Meyerhof pathway	\	Glycolysis
002C06	1	Fructose-bisphosphate aldolase 2 (Aldolase CE-2) [*Caenorhabditis elegans*]	fructose-bisphosphate aldolase activity	glycolysis	\	Glycolysis
0005C09	1	UTP--ammonia ligase [*Brugia malayi*]	CTP synthase activity	pyrimidine nucleotide biosynthesis; glutamine metabolism	\	Nucleotides metabolism
0007C01	2	DeoxyUTPase family member (dut-1) [*Caenorhabditis elegans*]	dUTP diphosphatase activity	dUTP metabolism	nucleus; cytoplasm	Nucleotides metabolism
00010F01	5	Ferritin protein 2 [*Caenorhabditis elegans*]	ferroxidase activity	iron ion transport; iron ion homeostasis	cytoplasm	iron ion transport
0006E12	1	Hypothetical protein CBG09375 [*Caenorhabditis*	aminoacyl-tRNA	osmoregulation; positive	\	Translation

		briggsae AF16]	hydrolase activity	regulation of growth rate		associated
003C04	1	TruB pseudouridine (psi) synthase homolog 2 [*Xenopus tropicalis*]	isomerase activity	tRNA processing	\	RNA modification
002F11	1	PAP/25A associated domain containing protein [*Brugia malayi*]	nucleotidyltransferase activity	\	intracellular	RNA tailing
0007H10	1	Oligosaccharyl transferase subunit STT3 homolog [*Caenorhabditis briggsae*]	oligosaccharyl transferase activity	reproduction; larval development; *	integral to membrane	Protein modification
001C02	1	UDP-GlucuronosylTransferase family member (ugt-61) [*Caenorhabditis elegans*]	glucuronosyltransferase activity	metabolism	integral to membrane	Post Translation Modification
0006D06	1	ABC transporter family protein [*Brugia malayi*]	ATPase activity	\	membrane	transporter
0007C06	1	Vacuolar H ATPase family member (vha-9) [*Caenorhabditis elegans*]	hydrogen-transporting ATP synthase activity	reproduction; larval development; *	proton-transporting two-sector ATPase complex	Hydrogen-transporting
0009E02	1	2 (Zwei) IG-domain protein family member (zig-1)[*Caenorhabditis elegans*]	vascular endothelial growth factor receptor activity	protein amino acid phosphorylation; regulation of Rho protein signal transduction	intracellular	?
0005A11	1	PREDICTED: similar to Presenilin CG18803-PB, isoform B [Apis mellifera]	peptidase activity	Notch signaling pathway; intracellular signaling cascade	integral to membrane	?
0009E05	1	Hypothetical protein CBG19720 [*Caenorhabditis briggsae AF16*]	protein serine and threonine kinase activity	protein amino acid phosphorylation	cytoplasm	?

15G10	1	ribokinase [Desulfatibacillum alkenivorans AK-01]	ribokinase activity	D-ribose metabolism	\	?
00012G04	2	Immunoglobulin I-set domain containing protein [*Brugia malayi*]	protein serine and threonine kinase activity	striated muscle contraction; mitosis; *	condensed nuclear chromosome; Z disc	?
00010D06	1	PREDICTED: similar to ecsit (evolutionarily conserved signaling intermediate in toll pathways) [*Nasonia vitripennis*]	oxidoreductase activity, acting on NADH and NADPH	innate immune response; regulation of oxidoreductase activity	nucleus; mitochondrion	?
004E05	1	Prion-like-(Q/N-rich)-domain-bearing protein family member (pqn-68) [*Caenorhabditis elegans*]	hydrolase activity, acting on ester bonds	D-amino acid catabolism	cytoplasm	?
00010F07	2	Prion-like-(Q/N-rich)-domain-bearing protein family member (pqn-48) [*Caenorhabditis elegans*]	oxidoreductase activity	antigen processing; exogenous antigen via MHC class II	extracellular region; lysosome	?
0009C10	1	PREDICTED: similar to epidermal retinal dehydrogenase 2 isoform 2 [*Macaca mulatta*]	oxidoreductase activity	metabolism	integral to membrane	?
0014G11	1	small fragment nuclease [*Danio rerio*]	exonuclease activity	\	mitochondrion	?
0009A08	1	protein binding / zinc ion binding [*Arabidopsis thaliana*]	ubiquitin-protein ligase activity	protein polyubiquitination; mitosis; *	nucleus	?
0008C10	1	ZDHHC14 protein [*Homo sapiens*]	acyltransferase activity	\	integral to membrane	?

N indicates copies of cDNA in the first batch sequencing. * indicates more than two items base on Gene ontology, and the other terms could be found in table S4. \ indicates there was no item description for cDNA based on Gene ontology. Some cDNAs factually could not be predicted, but with some others owing to not full-length of cDNA sequence. ?, Not sure what metabolism pathway related.
