# Supplementary material for: Preliminary molecular characterization of the human pathogen Angiostrongylus cantonensis
Source: BMC Mol Biol. 2009 Oct 25;10:97. doi: 10.1186/1471-2199-10-97 (PMC2774698; doi:10.1186/1471-2199-10-97)
Supplement: Additional file 6 — Sequence alignment and conserved feature or sites of four gene products. The data provided represent the analysis of sequence alignment and conserved feature or sites of four gene products, including Aspartic Protease, cystatin, Intermediate filaments (IFs) and Lactic acid dehydrogenase (LDH). [file 1471-2199-10-97-S6.PDF]

Additional file 6. Sequence alignment and conserved feature or sites of four gene products

1. Aspartic Protease

Graphic Summary

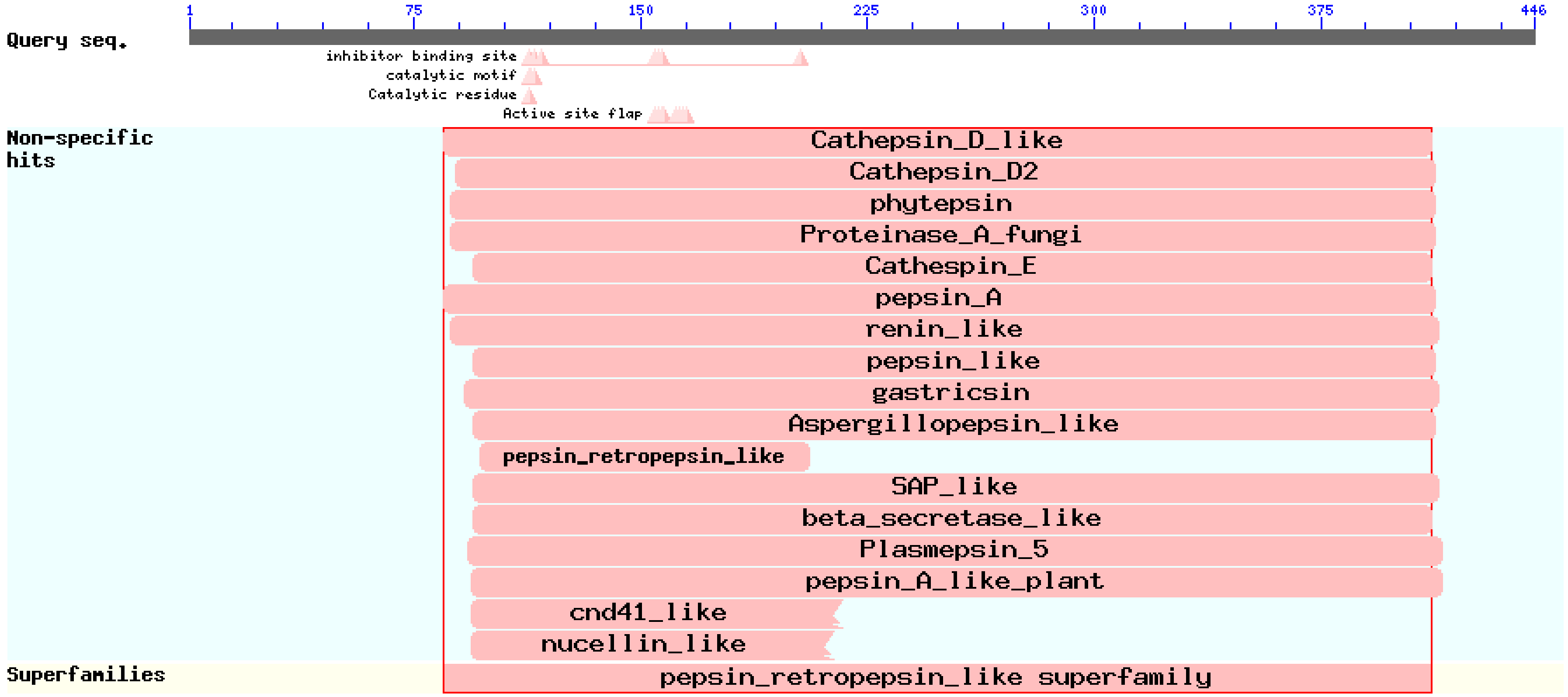

Sequence Alignment

Double underline indicates signal peptide of Aspartic Protease of *A. cantonensis*

```
                              10        20        30        40        50        60        70        80
                         ....|....|....|....|....|....|....|....|....|....|....|....|....|....|....|....|
Ac_Asp              1    ----MVRLLLILAFCTISLSHLHGQKPNHLTNNMMSVPLSRQPTLRERLYETGNWNSYERQR--GNYQRKLLAK--YAAN
Na_necepsin_2       1    ----MARLVFLLVLCTLAAASVHRRLFHQARRHVTSVSLSRQPTLRERLIASGSWEDYQKQR--YHYRKKILAK--YAAN
Ce_Asp_4            1    ----MNRCILLLLGALLLVQGLH---VHKRQQKLRTVSLKKQPTLRETLLQAGSFETFAKHR--HGYKKYLKTNGNHHFD
Bm_Asp_2            1    MLTAFATALLSIHLLIITLS----TVTATQQNHFTRIPLRKQNSLRKHLIETDSWEAYSKLINFQIQRKKIQQKMGLNLH
Ov_Asp              1    MIKTFATTLLSI-LTIITLCNEQITGIAAEENHFTRIALHKQDSIHSHLLKAGSWEAYSELVNFQIQRKRIQHKY--EFG
Clustal Consensus   1        :   :: :    :              .:.  :.* :* :::. *  :..:: : .       :: :  :

                              90       100       110       120       130       140       150       160
                         ....|....|....|....|....|....|....|....|....|....|....|....|....|....|....|
Ac_Asp              73   QAGRLRSTSEIDEFLRNYMDAQYFGTIQIGTPAQNFTVIFDTGSSNLWVPSKKCPFYDIACMLHHRYNSAASSTYVADGR
Na_necepsin_2       73   KASKLQSANEIDELLRNYMDAQYYGVIQIGTPAQNFTVIFDTGSSNLWVPSRKCPFYDIACMLHHRYDSGASSTYKEDGR
Ce_Asp_4            72   KYQALNVEGEIDELLRNYMDAQYFGTISIGTPAQNFTVIFDTGSSNLWIPSKKCPFYDIACMLHHRYDSKSSSTYKEDGR
Bm_Asp_2            77   FTSEPNAISETDEVLKNYMDAQYYGEISIGTPPQNFSVVFDTGSSNLWVPSVKCPFLDIACLFHNKYKGTKSTTYKPDGR
Ov_Asp              78   SRSGKSIAGETDEVLKNYMDAQYYGEISIGTPPQNFSVIFDTGSSNLWIPSIKCPFLDIACLLHNKYKGTESKTYKSDGR
Clustal Consensus   22           .* **.*:*******:* *.****.***:*:*********:** **** ****::*::*..  *.**  ***

                             170       180       190       200       210       220       230       240
                         ....|....|....|....|....|....|....|....|....|....|....|....|....|....|....|
Ac_Asp              153  KMGIQYGTGSMKGFISKDDVCIATICAEGQAFAEATSEPGIAFVVAKFDGILGMGFQEISVLGITPVFQTFIDQKKVPNP
Na_necepsin_2       153  KMAIQYGTGSMKGFISKDIVCIAGICAEEQPFAEATSEPGLTFIAAKFDGILGMAFPEIAVLGVTPVFHTFIEQKKVPSP
Ce_Asp_4            152  KMAIQYGTGSMKGFISKDSVCVAGVCAEDQPFAEATSEPGITFVAAKFDGILGMAYPEIAVLGVQPVFNTLFEQKKVPSN
Bm_Asp_2            157  KIQIQYGTGSMEGFISLDTVCIANICVTGQPFAEATSEPGATFVMAKFDGILGMAFPEISVLGLNPVFHTMISQKVVHQP
Ov_Asp              158  KIEIQYGRGSMKGFVSMDTVCIADVCVTDQPFAEATSEPGVTFIMAKFDGILGMAFPEIAVLGLSPVFNTMISQKVLQQP
Clustal Consensus   80   *: **** ***:**:* * **:* :*.   *.********* :*: *********.: **:***: ***:*::.** : .

                             250       260       270       280       290       300       310       320
                         ....|....|....|....|....|....|....|....|....|....|....|....|....|....|....|
Ac_Asp              233  MFAFWLNRNPYSELGGEITLGGMDSRRYFEPIIWTDVTHREYWQFKMDKIKGGSSIVACPNGCQAIADTGTSLIAGPKVQ
Na_necepsin_2       233  VFAFWLNRNPESEIGGEITFGGVDTRRYVEPITWTPVTRRGYWQFKMDMVQGGSSSIACPNGCQAIADTGTSLIAGPKAQ
Ce_Asp_4            232  LFSFWLNRNPDSEIGGEITFGGIDSRRYVEPITYVPVTRKGYWQFKMDKVVG-SGVLGCSNGCQAIADTGTSLIAGPKAQ
Bm_Asp_2            237  VFAFWLDRNPSDKIGGEITFGGIDANRFVSPITYTPVSRHGYWQFKMDRVLGRGKAIGCGNGCQAIADTGTSLIAGPKSQ
Ov_Asp              238  VFAFWLDRNPSDEVGGEITLGGIDTNRFVSPITYTPVSRHGYWQFKMDSIQGKDEAIGCANGCQAIADTGTSLIAGQKVK
Clustal Consensus   141  :*:***:*** .::*****:**:*:.*:..** :. *::: ******* : * .  :.* *************** * :
```

Box indicates catalytic residue(Feature 1)

Box and italic indicates Active site (Feature 2)

Posttranslational cleavage site is located between the two highlighted regions (Feature 3)

Box indicates catalytic residue(Feature 1)

```
                            330       340       350       360       370       380       390       400
                        ....|....|....|....|....|....|....|....|....|....|....|....|....|....|....|....|
Ac_Asp              313 VEAIQKYIGAEPLMRGEYMIPCDKVPSLPDISFVIENNEFVLKGVDYVLNVTTAGKSICLSGFMGIDLPEKIGELWILGD
Na_necepsin_2       313 VEAIQKYIGAEPLMKGEYMIPCDKVPSLPDVSFIIDGKTFTLKGEDYVLTVKAAGKSICLSGFMGMDFPEKIGELWILGD
Ce_Asp_4            311 IEAIQNFIGAEPLIKGEYMISCDKVPTLPPVSFVIGGQEFSLKGEDYVLKVSQGGKTICLSGFMGIDLPERVGELWILGD
Bm_Asp_2            317 IDKIQEYIGAEHVYAGEYIIPCYKVPSLPEITFVIAGKSYTLKGSDYVLNVTSKGATICLSGFMGIDLPKRVGELWILGD
Ov_Asp              318 LIKFSNILVLNMCMPGEYIIPCYKVSSLPEITFVIAGKSYTLKGSDYVFECNNKGKSICLSGSMGIDLPERLGELWILGD
Clustal Consensus   202 :  :.: :  :    ***:*.* **.:** ::*:* .: : *** ***:  .  * :***** **:*:*::********

                            410       420       430       440       450
                        ....|....|....|....|....|....|....|....|....|....|.
Ac_Asp              393 VFIGRYYTVFDVGQTRLGFAQAKDEHGRAVPPSVQKFQATEFPGNKSDDEDVFD--
Na_necepsin_2       393 VFIGKYYTVFDVGQARVGFAQAKSEDGFPVGTPVRTFRQLQEDSD-SDEDDVFTF-
Ce_Asp_4            391 VFIGRYYSVFDFDQNRVGFAQAKTADGRPVDPAPRPFRSVFDNESEESMEQDDE--
Bm_Asp_2            397 VFIGRYYTVFDVGNSQIGFAQARDSAGKPIGKRVRTLVSEEEQQISHESLASTFND
Ov_Asp              398 VFIGRYYTVFDVGNSQIGFAQARDT-------------------------------
Clustal Consensus   256 ****:**:***..: ::*****:
```

Abreviation

Ac: *Angiostrongylus cantonensis*
Na: *Necator americanus*
Ce: *Caenorhabditis elegans*
Bm: *Brugia malayi*
Ov: *Onchocerca volvulus*

Feature 1: catalytic residue
Evidence:

- **Citation:**PMID 10531052, Science. 1999 Oct 22;286(5440):735-41
- **Comment:**The two ASPs at the active site plays key catalytic roles in the pepsin family and conserved for all family members.
- **Comment:**Predicted based on homologous proteins woth structures.

Feature 2: Active site flap
Evidence:

- **Citation:**PMID 339694, Adv Exp Med Biol. 1977;95:61-81
- **Comment:**an extended loop projecting over the cleft to form an 11-residue flap, which encloses substrates or inhibitors within the active site. It also comtribute three residues for substrate specificity.
- **Comment:**Predicted based on homologous proteins with structures.

Feature 3: posttranlational cleavage site
Evidence:

- **Comment:**Posttranslational cleavage site is located between the two highlighted blocks. Cleavage occurs in a processing region that appears as an insertion that vary in length and amino acid composition in different species.
- **Citation:**PMID 1522590, J Mol Biol. 1992, 5;227(1):265-70

Feature 4: inhibitor binding sites were not shown because of unavailable in database at present

2. cystatin

Graphic Summary

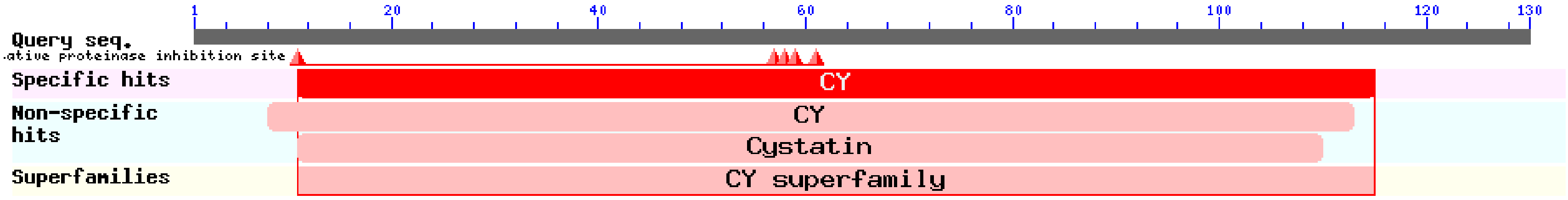

Sequence Alignment

```
                              10        20        30        40        50        60        70        80
                     ....|....|....|....|....|....|....|....|....|....|....|....|....|....|....|....|
Ac_cystatin        1 -----------------------??????????????AFATANTG-MVGGRTQQDASDPAYMERAWKAAKGINDDASNAG
Nb_nippocystatin   1 -----------------------MPSAFVLRIALASVVVTSTVSS-MVGGFTPQDVSDPEYMTRAWKAAKGINDDASNEG
Ce_CPI_2           1 -------------------------MKAILVFALIAISIISVNAGMMTGGSVEQDASQKEYSDKAWKAVKGINDQASNNG
Hc_cystatin_1      1 ---------------------------------------------MLAGGLTDQSTDDPEFMEQAWKAATKVNEEA-NDG
Bm_CPI_2           1 MMSTMSIKEGLLVILLSLFLFDTTALIHRREIPHMESKGQMQRGQVLLGGWQERSPEDNEILELLPSVLTKVNQQS--ND
Clustal Consensus  1                                               : **   :. .:        .. . :*:::   .

                              90       100       110       120       130       140       150       160
                     ....|....|....|....|....|....|....|....|....|....|....|....|....|....|....|....|
Ac_cystatin       43 PYHMMPIKVLKATSQVVAGVKYTFEALYGESTCKKGDFLAADLNASNCQLKSGGRRALYEVELWEKPWENFEQFNVKKLR
Nb_nippocystatin  57 PYHMIPVKILNAKTQVVAGVNHVFEVLFGESSCKKGDLSASELTATNCQLKEGGRKVIYEVHLWEKPWENFEQFNVKKVR
Ce_CPI_2          56 PYYYAPIKVTKASTQVVAGISTKLEVLVGESNCKKGELQAHEITSSNCQIKDGGSRALYQVTIWEKPWENFEQFTVEKIR
Hc_cystatin_1     35 DYYMIPTKVLSAKTQVVSGVMFTSKVLFEESFCKKGDVPVDQLKASNCAPKEGGKRVIYEISVLLQPWVKSEQVGVKVLR
Bm_CPI_2          79 EYHLMPIKLLKVSSQVVAGVKYKMEVQVARSECKKS--ASEQVNLKTCKKLEGHPDQVMTLEVWEKPWEDFLQVNILETK
Clustal Consensus 10  *:  * *: ...:***:*:    :.   .* ***.     ::. ..*   .*    :  : :  :** .  *. :   :
```

open brace indicates probably signal peptide domain of Ac_cystatin (5' terminal of signal peptide was incomplete at present)

Box indicates proteinase inhibition site(Feature 1)

Wave underlines indicates Cystatin domain

Box indicates proteinase inhibition site (Feature 1)

Wave underlines indicates Cystatin domain of Ac_cystatin

```
                        ....|...
Ac_cystatin         123 NVAADEEL
Nb_nippocystatin    137 TLAPGEQV
Ce_CPI_2            136 DVTADEQF
Hc_cystatin_1       115 VFDPGEQV
Bm_CPI_2            157 VLSSV---
Clustal Consensus   40   . .
```

Abreviation
Ac: *Angiostrongylus cantonensis*
Nb: *Nippostrongylus brasiliensis*
Ce: *Caenorhabditis elegans*
Bm: *Brugia malayi*
Hc: *Haemonchus contortus*

Feature 1: putative proteinase inhibition site
Evidence:

- **Comment:**n-terminal Gly is essential for binding to proteinase target
- **Citation:**PMID 2347312 (EMBO J. 1990 Jun;9(6):1939-47)
- **Structure:**1STF1, bound to papain

3. Intermediate filaments (IFs)

Graphic Summary

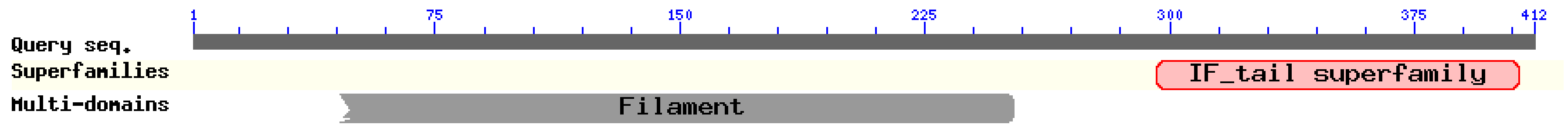

Sequence Alignment

```
                                   10        20        30        40        50        60        70        80
                           ....|....|....|....|....|....|....|....|....|....|....|....|....|....|....|....|
Bm_IFs               1     -------------------------------------------------------MSEYRSTISARPGYNRISSTTSSST
OV_IFs               1     --------------------------------------------------------------------------------
Al_IFs               1     MKANEKAFAAAALAFVREAVKLNDEIAPDNFNIRVQYVVAGSDRTNRRLTAAAAAMSDYRSTISARPGFNRTITSNASNA
Ac_IFs               1     --------------------------------------------------------------------------------
Ce_IFa_1             1     -------------------------------------------------------MSFTSTTPSARAPLG-------GRT
Clustal Consensus    1

                                   90       100       110       120       130       140       150       160
                           ....|....|....|....|....|....|....|....|....|....|....|....|....|....|....|....|
Bm_IFs               26    FGGLPSGNRVVKVVTEMSSSSLTSGMSPFGQNAASTIRDAREREKKEMSDLNDRLADYIEKVRFLEAQNRKLNADLDMLR
OV_IFs               1     ----------------MSSSSLTSGMSPFGQNAASTIRDAREREKKEMSDLNDRLADYIEKVRFLEAQNRKLNADLDMLR
Al_IFs               81    FGSLPSGNRVVKIVTEMSSSSMTSGMSPFGQNAASTIRDAREREKKEMSDLNDRLADYIEKVRFLEAQNRKLAADLDMLR
Ac_IFs               1     --------------------------------------------------------------------------------
Ce_IFa_1             19    TGNVTSQDRMLKIVTEMRSGAG-SGLSPFGQNAASTIRDSREREKKEMSDLNDRLASYIEKVRFLEAQNRKLAADLDALR
Clustal Consensus    1
```

Wave underlines indicates Filament domain of Ac_Ifs.
Here, amino acid sequences not known because of not full-length cDNA

```
                                  170       180       190       200       210       220       230       240
                           ....|....|....|....|....|....|....|....|....|....|....|....|....|....|....|....|
Bm_IFs               106   GRWGKDTSSIKIMFEGELSEAQKVISDTGKQRDELEKQIKKMQEELAEYRRKY-----ARLSDRDKIDELLVALSNIEAE
OV_IFs               65    GRMGKDTSSIKIMFEGELSEAKKIIGDTGKQRDELEKQIKKMQEEVAEYRRKFDEAKKARLTDRDQIDELLVALSNIEAE
Al_IFs               161   GRWGKDTSSIKIMYEGELSEARKVINDTAKQRDDLEKEIKKLQDELAEYRRKFDEATKARLVDREKIDELLVALSNIEAE
Ac_IFs               1     -------------------------------------------------------------EDREKIDELLVKLSNIEAE
Ce_IFa_1             98    SKWGKDTHNIRNMYEGELVDAQKLIDETNKQRKDMEGQLKKMQDELAEMRRKLEDATKGREQDRAKIDALLVTLSNLEAE
Clustal Consensus    1                                                                    ** :** *** ***:***
```

Wave underlines indicates Filament domain of Ac_Ifs.
Here, amino acid sequences not known because of not full-length cDNA

```
                            250       260       270       280       290       300       310       320
                    ....|....|....|....|....|....|....|....|....|....|....|....|....|....|....|....|
Bm_IFs             181 INLLRRRIALLEEEISRIKKENQRLIGELQRARTDLDQETLNRIDHQNQVQTLQEEIDFIRRVQDQEIRDLQAMAARDTT
OV_IFs             145 INLLRRRIALLEEEISRIKKENQRLVSELQRARTDLDQETLNRIDYQNQVQTLQEEIDFIRRVQDQEIRDLQAMAARDTT
Al_IFs             241 INLLRRRIALLEEEIARIKKENQRLVAELQRARTDLDQETLNRIDYQNQVQTLQEEIDFIRRVQDQEIRDLQAMAARDTT
Ac_IFs              20 INLLRRRIALLEEEISRLKKENMRLIAELQRARTDLDQETLNRIDYQNQVATLLEEIGFINRVHDQEIKELQAMAARDTT
Ce_IFa_1           178 ISLLKRRIAQLEDEVKRIKQENQRLLSELQRARTDLDQETLNRIDYQNQVQTLLEEIDFLRRVHDNEIKELQTLASRDTT
Clustal Consensus   16 *.**:**** **:*: *:*:** **:.*****************:**** ** ***.*:.**:*:**::**::*:****

                            330       340       350       360       370       380       390       400
                    ....|....|....|....|....|....|....|....|....|....|....|....|....|....|....|....|
Bm_IFs             261 SENREFFKNELSSAIRDIRNEYDQMTSANRNDMESWYKLKVQEIQTQSARQTMEQGYQREEVKRLRIQLGDLRGKLADLE
OV_IFs             225 SENREFFKNELSSAIRDIRNEYDQMTNANRNDMESWYKLKVQEIQTQSARQTMEQGYQREEVKRLRVQLGDLRGKLADLE
Al_IFs             321 SENREFFKNELSSAIRDIRNEYDQMTNANRNDMESWYKLKVQEIQTQSARHTMEQGYQKEEVKRLRTQLGDLRGKLADLE
Ac_IFs             100 SENREFFKNELASAIRDIRNEYDQMMNTNRNDMESWYKLKVQEIQTQSARQNMEQGYQKDEVKRLRTQLTDLRGKLADLE
Ce_IFa_1           258 PENREFFKNELSSAIRDIREEYDQVNNVHRNDMESWYRLKVQEIQTQSARQNMEQGYAKEEVKRLRTQLSDLRGKLADLE
Clustal Consensus   86 .**********:*******:****: ..:********:************:.*****  ::****** ** **********

                            410       420       430       440       450       460       470       480
                    ....|....|....|....|....|....|....|....|....|....|....|....|....|....|....|....|
Bm_IFs             341 GRNSLLEKQIQELNFQLEDDQRSYEAALNDRDAQIRKMREECQALMVELQMLLDTKQTLDAEIAIYRKMLEGEGDGPGLK
OV_IFs             305 GRNSLLEKQIQELNFQLEDDQRSYEAALNDRDAQIRKMREECQALMVELQMLLDTKQTLDAEIAIYRKMLEGEGDGPGLK
Al_IFs             401 GRNALLEKQIQELNFQLEDDQRSYEAALNDRDAQIRKMREECQALMVELQMLLDTKQTLDAEIAIYRKMLEGEGDGPGLR
Ac_IFs             180 GRNALLEKQIQELNYQLEDDQRSYEAALNDRDAQIRKMREECQALMVELQMLLDTKQTLDAEIAIYRKMLEGEGDGPGLR
Ce_IFa_1           338 SRNSLLEKQIQELNYQLEDDQRSYEAALNDRDSQIRKMREECQALMVELQMLLDTKQTLDAEIAIYRKMLEGEENRAGLK
Clustal Consensus  158 .**:**********:*****************:*************************************** : .**:

                            490       500       510       520       530       540       550       560
                    ....|....|....|....|....|....|....|....|....|....|....|....|....|....|....|....|
Bm_IFs             421 QLVEQVVRTTGINEVADTETMRVVKGETSSRT-SYTRSAKGNVSIQETSPDGKFIILENTHRSKEEPIGEWKLKRKIDGK
OV_IFs             385 QLVEQVVRTTGINEVADTETMRVVKGETSSQTRSYTRSEKGNVSIQETSPDGKFIVLENTHRSKEELIGEWKLKRKIDGK
Al_IFs             481 QLVEQVVRTTGINEVADTETMRVVKGETSSRT-SYTRSAKGNVSIQETSPDGKYIVLENTHRSKEEPIGEWKLKRKIDGK
Ac_IFs             260 QLVEQVVRTTGINEVADTETMRVVKGETSSRT-SYTRSAKGNVSIHDPSPEGKYVVLENIHRSKEEPIGDWKLKRKIDGK
Ce_IFa_1           418 QLVEQVVKTHAITQETDTETMRVVKGETASRQ-SFQRSAKGNVSIHEASPDGKFIVLQNTHRAKDEAIGEWKLKRRIDGK
Clustal Consensus  235 *******:* .*.: :************:*:  *: ** ******::.**:**:::*:* **:*:* **:*****:****
```

Wave underlines indicates Filament domain of Ac IFs

Wave underlines indicates IF tail superfamily of Ac IFs

Wave underlines indicates IF tail superfamily of Ac IFs

Underlines indicates IF tail superfamily of Ac IFs

```
                           570       580       590       600       610       620       630
                   ....|....|....|....|....|....|....|....|....|....|....|....|....|....|....
Bm_IFs             500 REIVYTLPRDFILKPSKTVKIWSRGQGGVHAPPEQLIFESEESFGVGNNVQTILYNKEGEERATHIQRSSQTVS
OV_IFs             465 REIVYTLPRDFILKPSKTVKIWGRGQGGVHAPPEQLIFEAEESFGMGSNVQTILYNKEGEERATHIQRSSQTIS
Al_IFs             560 KEIVYTLPKDFILKPCKHVKIWARGQGGIHAPPEQLIFEGEDSFGVGSNVQTILYNREGEERATHIQRSSQTSS
Ac_IFs             339 REIVYSIPHDFVLKAGKSVKIWARGQGGVHNPPESLVFDGEDSFGAGSNVQTILYNSEGEERATHIQRQSQSAT
Ce_IFa_1           497 RENVYTLPRDFVLRAGKTLKIFARNQG-VASPPDQLVYDAEDSFGSGNNVQTILFNKEGEERATHIQRQSTA--
Clustal Consensus  304 :* **::*:**:*:. * :**:.*.** :  **:.*:::.*:*** *.******:* ***********.* :
```

Underlines indicates IF tail superfamily of Ac IFs

Abreviation

Ac: *Angiostrongylus cantonensis*
Ce: *Caenorhabditis elegans*
Bm: *Brugia malayi*
Al: *Ascaris lumbricoides*
Ov: *Onchocerca volvulus*

PubMed References: Intermediate filament proteins, Protein Profile 1995; 2(8):795-952

4. Lactic acid dehydrogenase (LDH).

Graphic Summary

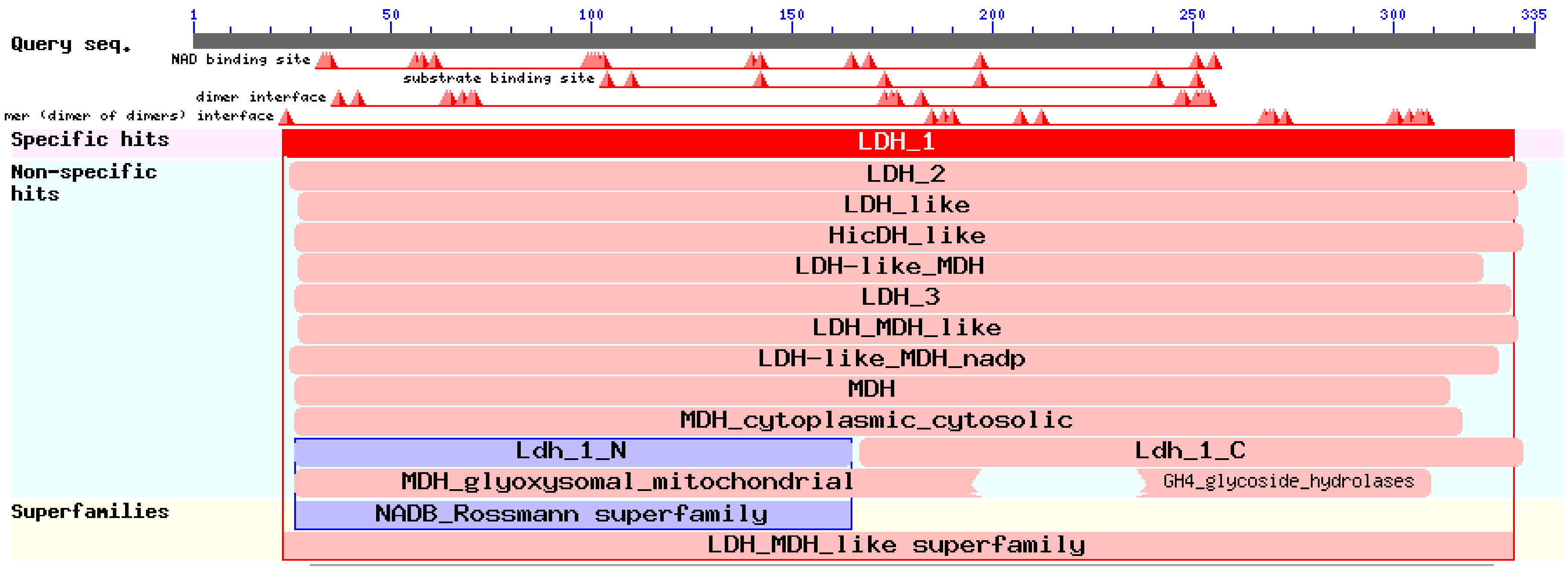

Sequence Alignment

```
                              10        20        30        40        50        60        70        80
                      ....|....|....|....|....|....|....|....|....|....|....|....|....|....|....|....|
Mm_LDH_b           1  ------------------MATLKEKLIASVADDEAAVPNNKITVVGVGQVGMACAISILGKSLADELALVDVLEDKLKGE
Rn_LDH_b           1  ------------------MATLKEKLIAPVADDETAVPNNKITVVGVGQVGMACAISILGKSLADELALVDVLEDKLKGE
Hs_LDH_b           1  ------------------MATLKEKLIAPVAEEEATVPNNKITVVGVGQVGMACAISILGKSLADELALVDVLEDKLKGE
Ce_ldh_1           1  ------------------MASTIKEVFAEIAAPVENS-HGKVTVVGVGQVGMACAYSILQQNLANELCLVDVVADKLKGE
Bm_LDH             1  MFAFLGRLEFIRQLVTVRMSETTAALFKEIVPAHRTP-HSKVTVVGVGQVGMACAVSIMQKNIVSELCLVDVVADKLKGE
Ac_ldh_1           1  --------------MNCETAKMVEALMEQVTPAAART-TSKVTIVGVGQVGMACAYSMLQQNIAGELCMVDVVADKLKGE
Clustal Consensus  1                     :     ::  :.        .*:*:*********** *:: :.:..**.:***: ******
```

Box indicates NAD binding site (Fiture 1)

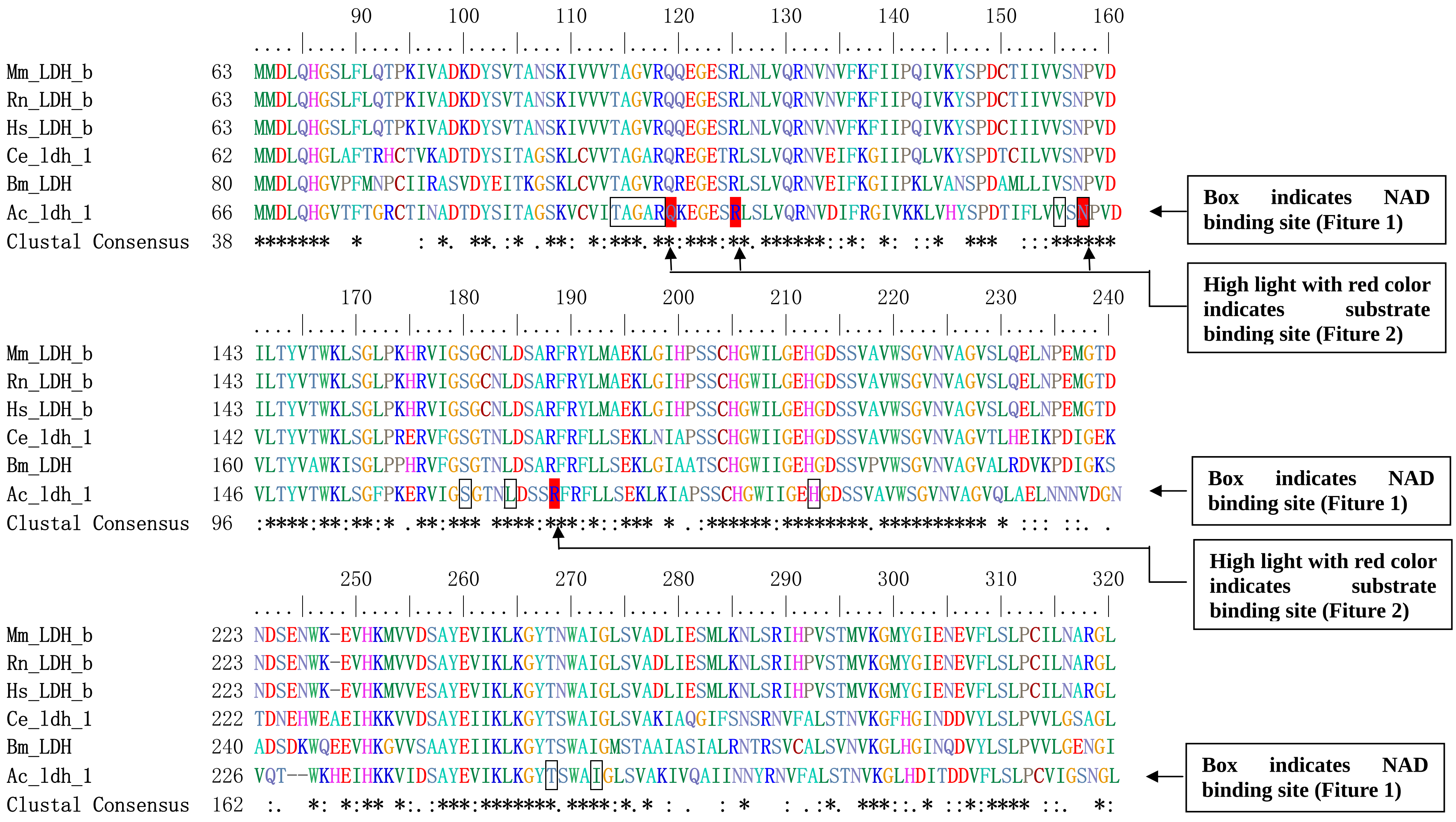

Mm_LDH_b
Rn_LDH_b
Hs_LDH_b
Ce_ldh_1
Bm_LDH
Ac_ldh_1
Clustal Consensus
Box indicates NAD binding site (Fiture 1)
High light with red color indicates substrate binding site (Fiture 2)
Box indicates NAD binding site (Fiture 1)
High light with red color indicates substrate binding site (Fiture 2)
Box indicates NAD binding site (Fiture 1)

```
                           330       340       350
                   ....|....|....|....|....|....|...
Mm_LDH_b           302 TSVINQKLKDDEVAQLRKSADTLWDIQKDLKDL
Rn_LDH_b           302 TSVINQKLKDDEVAQLRKSADTLWDIQKDLKDL
Hs_LDH_b           302 TSVINQKLKDDEVAQLKKSADTLWDIQKDLKDL
Ce_ldh_1           302 THVVKQQLTEAEVQKLHNSAKALLEVQNGIVM-
Bm_LDH             320 THIVKQNLNETEIKQLQKSAAQLYEVQCGIKGL
Ac_ldh_1           304 THIIKQHLNKDELSTLHKSWKTLVEVQNKLQL-
Clustal Consensus  215 * :::*:*.. *:  *::*   * ::*  :
```

Abreviation

Ac: *Angiostrongylus cantonensis*

Ce: *Caenorhabditis elegans*

Bm: *Brugia malayi*

Mm: *Mus musculus*

Rn: *Rattus norvegicus*

Hs: *Homo sapiens*
