# Supplementary material for: Preliminary molecular characterization of the human pathogen Angiostrongylus cantonensis
Source: BMC Mol Biol. 2009 Oct 25;10:97. doi: 10.1186/1471-2199-10-97 (PMC2774698; doi:10.1186/1471-2199-10-97)
Supplement: Additional file 7 — Sensitivities and specificities of crude antigen, ES antigen and four candidate recombinant proteins. The data provided represent the diagnostic analysis of four recombinant proteins. Data were derived from table 2 in text. Sensitivities and specificities analysis were referred to Intapan. TP: True Positive; TN: True Negative; FP: False Positive; FN: False Negative. Sensitivity = No. of TP/(No. of TP+No. of FN);Specificity = No. of TN/(No. of TN+No. of FP). [file 1471-2199-10-97-S7.PDF]

Additional file 7. Sensitivities and specificities of crude antigen, ES antigen and four candidate recombinant proteins.

Crude antigen	Proven human angiostrongyliasis	Suspected human angiostrongyliasis	Other parasitic diseases	Healthy control	
Positive	4 (TP)	6	2	0	2 (FP)
Negative	0 (FN)	5	20	4	24 (TN)
Total	4	11	22	4	

Sensitivity = 4/(4+0)=100% ; Specificity = 24/(24+2)=92.3%

ES	Proven human angiostrongyliasis	Suspected human angiostrongyliasis	Other parasitic diseases	Healthy control	
Positive	4 (TP)	8	5	0	5 (FP)
Negative	0 (FN)	3	17	4	21 (TN)
Total	4	11	22	4	

Sensitivity = 4/(4+0)=100% ; Specificity = 21/(21+5)=80.8%

Recombinant aspartic protease	Proven human angiostrongyliasis	Suspected human angiostrongyliasis	Other parasitic diseases	Healthy control	
Positive	3 (TP)	2	0	0	0 (FP)
Negative	1 (FN)	9	22	4	26 (TN)
Total	4	11	22	4	

Sensitivity = 3/(3+1)=75% ; Specificity = 26/(26+0)=100%

Recombinant cystatin	Proven human angiostrongyliasis	Suspected human angiostrongyliasis	Other parasitic diseases	Healthy control	
Positive	3 (TP)	2	0	0	0 (FP)
Negative	1 (FN)	9	22	4	26 (TN)
Total	4	11	22	4	

Sensitivity = 3/(3+1)=75% ; Specificity = 26/(26+0)=100%

Recombinant IFs	Proven human angiostrongyliasis	Suspected human angiostrongyliasis	Other parasitic diseases	Healthy control	
Positive	2 (TP)	3	0	0	0 (FP)
Negative	2 (FN)	8	22	4	26 (TN)
Total	4	11	22	4	

Sensitivity = 2/(2+2)=50% ; Specificity = 26/(26+0)=100%

Recombinant LDH	Proven human angiostrongyliasis	Suspected human angiostrongyliasis	Other parasitic diseases	Healthy control	
Positive	2 (TP)	2	0	0	0 (FP)
Negative	2 (FN)	9	22	4	26 (TN)
Total	4	11	22	4	

Sensitivity = 2/(2+2)=50% ; Specificity = 26/(26+0)=100%

Data were derived from table 2 in text. Sensitivities and Specificities analysis were referred to Intapan. TP: True Positive; TN: True Negative; FP: False Positive; FN: False Negative. Sensitivity=No. of TP/(No. of TP＋No. of FN);Specificity=No. of TN/(No. of TN＋No. of FP).
