# Supplementary figures and images for: Preliminary molecular characterization of the human pathogen Angiostrongylus cantonensis
Source: BMC Mol Biol. 2009 Oct 25;10:97. doi: 10.1186/1471-2199-10-97 (PMC2774698; doi:10.1186/1471-2199-10-97)

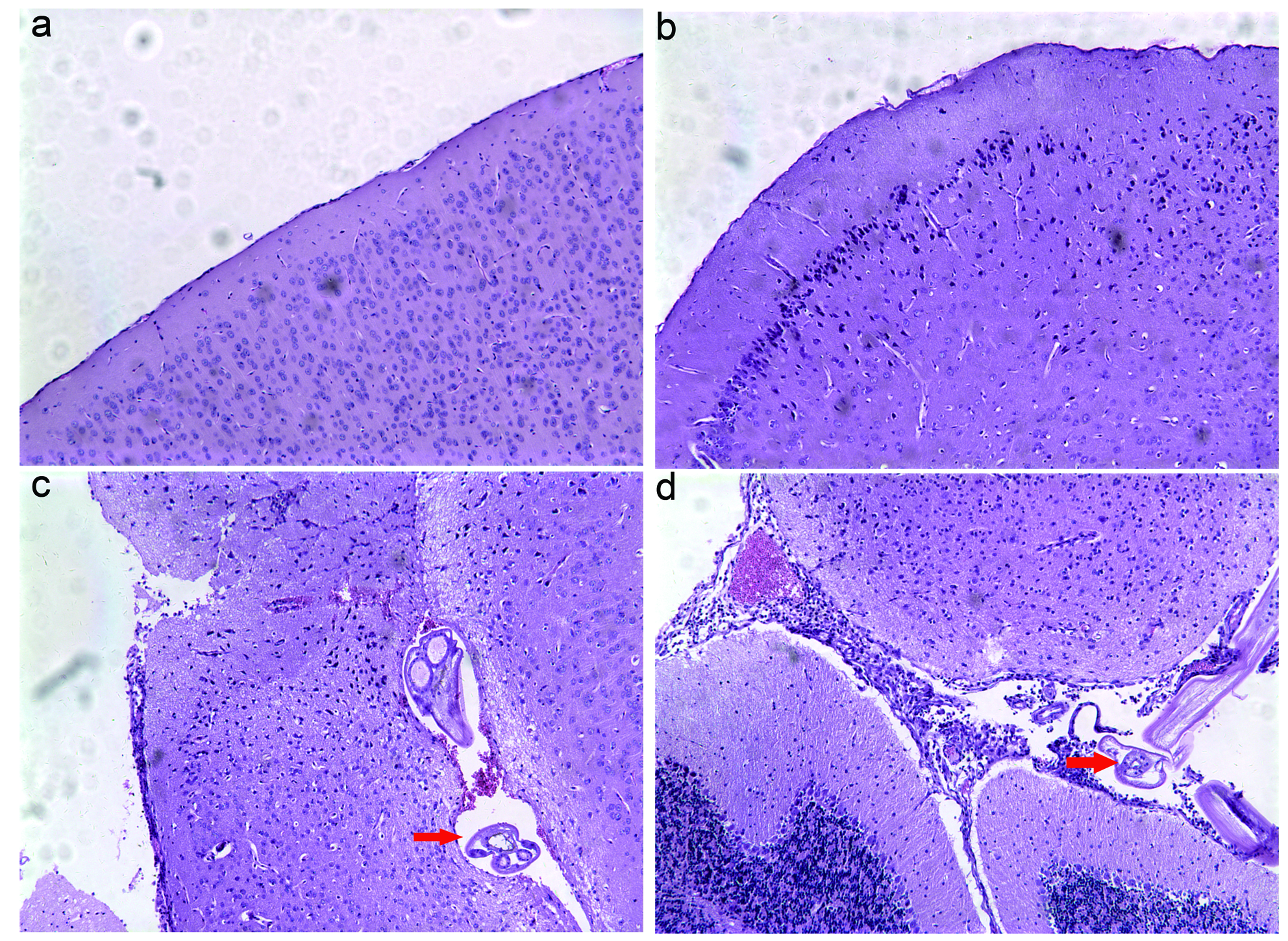

Supplement: Additional file 8 — Pathological changes in the brains of mice experimentally infected with A. cantonensis. The data provided represent the vaccine potential analysis of recombinant cystatin. Pathological changes in the brains of mouse experimentally infected with A. cantonensis (haematoxylin and eosin staining; 100×, day 21). (a) Healthy group; (b) Group vaccinated with protein of cystatin. These two groups were not infected larvae; (c) Group only vaccinated with Freund's adjuvant; (d) vaccinated with cystatin. These two groups were infected with L3 larvae after vaccinated. Red arrows signal cutting plane of larvae which were surrounded by eosinophils, inflammatory and glial cells. [file 1471-2199-10-97-S8.TIFF]
